# The variational modulus density theory explains mechanical responses of cell membranes and membrane crosslinkers

**DOI:** 10.1038/s41598-025-17573-2

**Published:** 2025-10-16

**Authors:** Jichul Kim

**Affiliations:** Department of Biomedical Engineering, Dongguk University, Seoul, 04620 Republic of Korea

**Keywords:** Membrane-crosslinker interaction, Confined Brownian motion, Quantum wavefunction, Lipid nanodomain, Adhesion molecular machinery, Finite element method, Membrane biophysics, Mechanical engineering, Computational science, Biological physics, Applied physics

## Abstract

Both classical mechanics and quantum mechanics explain Brownian motion. However, it remains unclear whether they are compatible with each other, as the physical and mathematical identity of the wavefunction in quantum mechanics has been elusive. In this work, a continuum theory using grammars in classical mechanics modeling, but potentially compatible with the quantum wavefunction, is introduced. The theory explains the confined Brownian motion of cell membrane inclusions interacting with extracellular matrices or cytoskeletons via elastic molecular crosslinkers. This crosslinker theory is integrated into the Canham-Helfrich-Evans model for fluid membranes. Calculations, based on a finite element method for the combined theory, reproduced measured data from adhesion molecular machineries and cell membranes. Overall, by providing physical and mathematical interpretations of the quantum wavefunction, the presented theoretical model provides improved capabilities for the realistic simulation of cell membranes and membrane linker proteins.

## Introduction

Understanding interactions between cell membranes and crosslinkers that connect the membrane to extracellular matrices (ECMs) or cytoskeletons is crucial for studying cellular signaling across cell membranes. A variety of adhesion receptors and cytoplasmic adaptors that act as crosslinkers have been identified. For example, activated integrins inserted into the membrane mechanically interact with ECMs to facilitate migration, gene expression, and homeostasis^1^. The talin adaptor, which interacts with membrane-inserted integrins, forms physical connections between membranes and cytoskeletons to support various physiological processes^2,3^. Similarly, CD44 membrane receptors are linked to underlying cytoskeletons to mediate cellular signaling^4,5^. Despite rapid advances in experimental technologies and the accumulation of a vast amount of measured data on membrane-crosslinker interaction, current theoretical continuum models do not fully incorporate these experimental developments.

The membrane-crosslinker interaction is complex. One end of the crosslinker is linked to a membrane-inclusion molecule (or domain) inserted into the membrane characterized by a certain lipid environment, while the other end is fixed on ECMs or cytoskeletons. Therefore, one end of the crosslinker exhibits confined Brownian motion on the surface of a deforming fluid membrane. At the same time, the spring-like crosslinker can be stretched and relaxed due to applied forces. In addition to these physical complexities, there exists a viewpoint that considers the Brownian motion as a quantum phenomenon^6–8^, while cell membranes have been explained by classical continuum mechanics^9–11^. A unified theoretical and mathematical framework for classical mechanics and quantum mechanics is thus required for realistic calculations of the membrane-crosslinker complex.

**Fig. 1:**
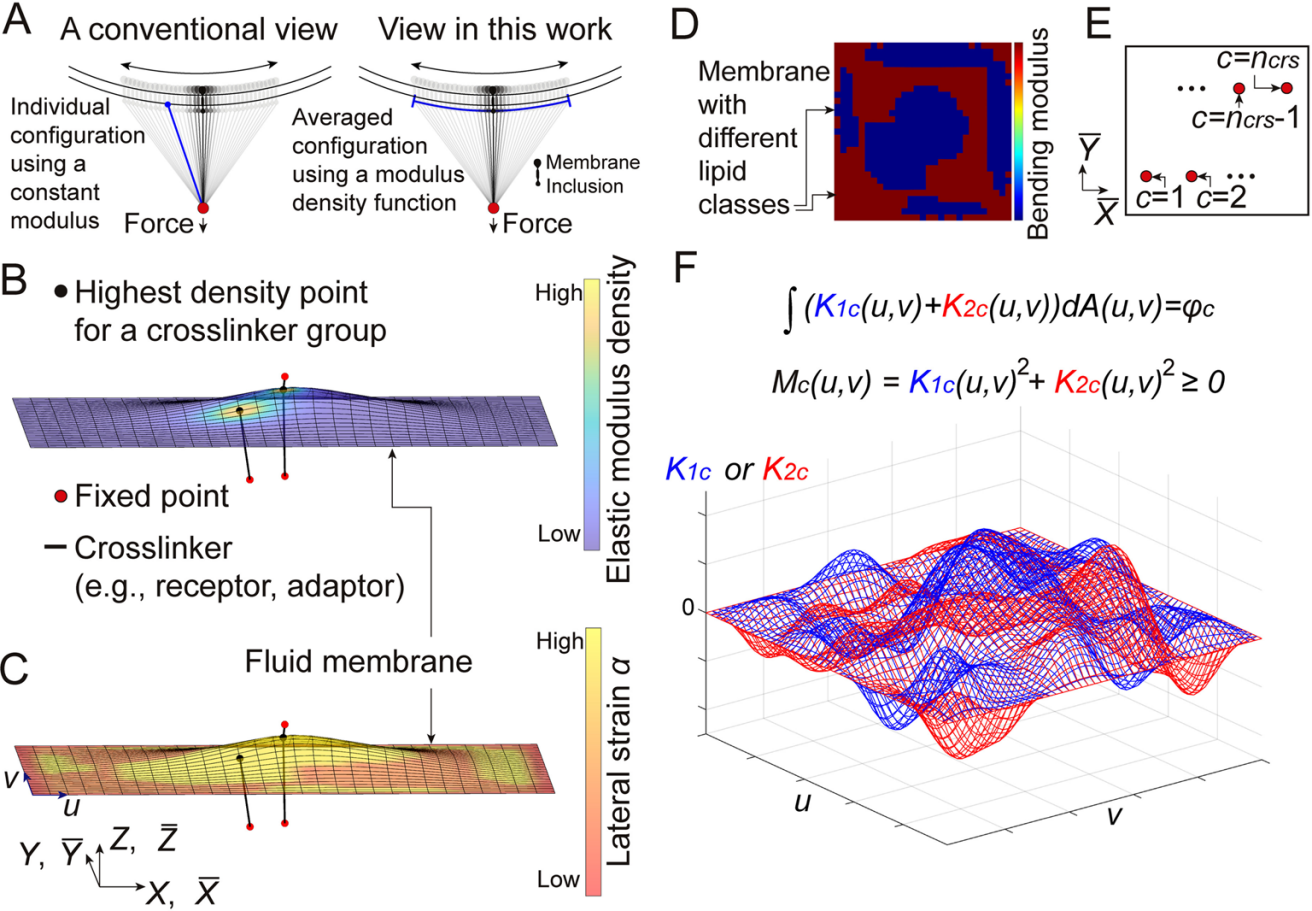
Descriptions of the membrane-crosslinker model. (**A**) Two different views in mathematical modeling for the confined Brownian motion of crosslinkers based on conventional elasticity (left) and the modulus density theory proposed in this work (right). (**A, left**) The conventional viewpoint. Similar to an elastic spring on the solid membrane, the crosslinker force at a fixed time point is calculated using a constant elastic modulus. (**A, right**) The alternative viewpoint (in this work). As a modeling treatment, the average distribution of the mobile crosslinker was considered to calculate the crosslinker force. (**B‒D**) An example configuration calculated using the finite element model of membrane-crosslinker complexes. (**B**) The elastic modulus density is shown in the surface map. (**C**) Lateral lipid number strain of the membrane is shown in the surface map. (**D**) The bending moduli of different lipid classes in the membrane are shown in the surface map. (**E**) Description of how to index the group of crosslinkers in this model. (**F**) Description of how the constant ${\varphi }_{c}$ can be split into real numbers assigned over a parametric surface using two degrees of freedom (DOFs) at each point. The functions ${K}_{1c}$ and ${K}_{2c}$ determine values for the two DOFs. With two DOFs, both “positive” and “negative” values can be independently considered at each point during the decomposition of ${\varphi }_{c}$. Such a mathematical strategy may maximize the variability of the shape of the modulus density function ${M}_{c}$ made from the given ${\varphi }_{c}$ value. The illustration here depicts the region where the modulus density is non-zero (except the boundary).

In this work, two simple yet novel principles are proposed to incorporate all these complexities and discrepancies into a single variational framework. First, the average of multiple configurations of the crosslinker under confined diffusion can be considered to calculate the crosslinker force (Fig. 1A, right). It is assumed that, during confined diffusion, forces at the fixed end of the crosslinker are constant if all the other boundaries in the membrane-crosslinker complex are also fixed (even though the mobile end is under confined diffusion). This assumption aligns with the notion that membrane curvature change and lateral stretching are negligible during the confined diffusion, because a mechanical change of the membrane can alter the crosslinker force. In this case, the constant crosslinker force can be calculated by finding the average configuration of the crosslinker under the confined diffusion, because the single constant force representing all individual crosslinker configurations is the same as the averaged force, even though the new configuration after the averaging process may differ from those individual configurations. In continuum theory, finding the average configuration of the mobile crosslinker is equivalent to finding the modulus density profile on the membrane that is used to determine the averaged distribution of internal elastic energies from the individual configurations of the crosslinker under the confined diffusion (Fig. 1B). The approach here is, therefore, statistical and independent of the time scale of confined diffusion, even though confined diffusion itself is a time-dependent process.

Second, another assumption is made that any confined Brownian motion of a mobile membrane inclusion linked to a crosslinker and embedded within a certain lipid environment (i.e., all possible elastic modulus density profiles) is a physical output that depends on a certain intrinsic physical constant of the inclusion-crosslinker complex. Therefore, a combined mathematical framework is introduced to generate various functions with values greater than or equal to zero (i.e., various configurations for the modulus density profile) on a two-dimensional surface as many as possible, by using a single constant value. This is achieved by defining the modulus density value at each point with two degrees of freedom (DOFs) (using functions ${K}_{1c}$ and ${K}_{2c}$, see Fig. 1F). The sum of squares of these two ${K}_{1c}$ and ${K}_{2c}$ values is the modulus density at each point (${M}_{c}={{K}_{1c}}^{2}+{{K}_{2c}}^{2}$, see Fig. 1F and Eq. 6 in Results). To correlate the two-dimensional functions ${K}_{1c}$ and ${K}_{2c}$ with the single constant value, it is necessary to employ the integral operator. The area integral of the functions serves as the intrinsic constant for the inclusion-crosslinker complex embedded in a certain lipid environment ($\int \left({K}_{1c}+{K}_{2c}\right)dA={\varphi }_{c}$, see Fig. 1F, the relation is implemented in Eq. 1).

There must be many configurations for the function ${M}_{c}$ that can yield the same constant ${\varphi }_{c}$ when the function ${K}_{1c}$ (without ${K}_{2c}$) is integrated over the surface. However, “plus” and “minus” signs are two fundamental operators in integration. To enhance the variability of the shape of the function ${M}_{c}$ made from the ${\varphi }_{c}$ constant, therefore, two DOFs using ${K}_{1c}$ and ${K}_{2c}$ are required so that both the “plus” and “minus” operations can be independently considered at each point on the surface in the integration process leading to the constant ${\varphi }_{c}$. In other words, the two DOFs framework allows the most dynamic decomposition of the single constant ${\varphi }_{c}$ into real numbers distributed over the continuous membrane surface. Having both the “positive” and “negative” components at each point still guarantees the modulus density value that is greater than or equal to zero. Additional DOFs beyond these two at each point might be redundant in considering the two operations, and thus may not affect the final results. With the foregoing treatments using ${K}_{1c}$, ${K}_{2c}$, and ${\varphi }_{c}$, therefore, the maximum variability of the shape of the modulus density function ${M}_{c}$, with respect to the given constant ${\varphi }_{c}$, might be achieved. Finally, we can generalize that all physically possible confined diffusion configurations (i.e., all physically possible ${M}_{c}$ functions) for a certain membrane inclusion, linked to a certain crosslinker and interacting with a certain lipid environment, might be generated using this mathematical framework. Among the infinitely many possibilities, however, the principle of stationary total potential energy provides one unique ${M}_{c}$ profile.

This crosslinker theory is coupled with continuum models for lipid membranes^11–15^. Calculations, performed using a finite element method for the combined model (Fig. 1B‒D), directly reproduced numerous experimental data on adhesion molecular machineries and cell membranes. Furthermore, the model provides biological insight into the following: how cell membranes can be compartmentalized with a minimal number of membrane-cytoskeleton crosslinkers; how the presence of different lipid classes and their sorting affect the size of lipid reservoirs during mechanical responses of membranes; and how the new theory offers a more general framework for explaining one-dimensional crosslinker protein compared to conventional elasticity. Overall, the presented theoretical model provides invaluable biological insights into cell membranes and their interactions with crosslinkers.

## Results

### A combined continuum theory for cell membranes and membrane crosslinkers

The total functional ${\Pi }^\mathrm{tot}$, which describes mechanical responses of a cell membrane and elastic crosslinkers connected to mobile membrane inclusions, is defined in Eq. 1.

1 $${\Pi }^\mathrm{tot}=\int {E}^\mathrm{curv}dA+\int {E}^\mathrm{st}dA+{\sum }_{c=1}^{{n}_\mathrm{crs}}\int {E}_{c}^\mathrm{crs}dA+{\uplambda }^\mathrm{lipid}\left(\int \frac{{\phi }_\mathrm{0}}{\alpha +1}dA-{n}_\mathrm{lipid}\right)+{\sum }_{c=1}^{{n}_\mathrm{crs}}{\uplambda }_{c}^\mathrm{crs}\left(\int \left({K}_{1c}+{K}_{2c}\right)dA-{\varphi }_{c}\right)$$

where

2 $${E}^\mathrm{curv}=2{\kappa}{H}^{2}+{k}_\mathrm{g}G\quad\text{ for }\quad0\le \alpha \le {\alpha }_\mathrm{cross}$$

3 $${E}^\mathrm{st}=\frac{{\upsigma }_{0}}{{c}_{1}}\mathrm{exp}\left({c}_{1}\alpha \right)-\frac{{\upsigma }_{0}}{{c}_{1}}\quad\text{ for }\quad0\le \alpha \le {\alpha }_\mathrm{cross}$$

4 $${E}^\mathrm{st}=\frac{{K}_\mathrm{app}{\alpha }^{2}}{2}-{K}_\mathrm{app}{\alpha }_\mathrm{cut}\alpha +{c}_{2}\quad\text{ for }\quad\alpha>{\alpha }_\mathrm{cross}$$

5 $${E}_{c}^\mathrm{crs}=\frac{1}{3}{M}_{c}{\sqrt{{\left(X-{\overline{X} }_{c}\right)}^{2}+{\left(Y-{\overline{Y} }_{c}\right)}^{2}+{\left(Z-{\overline{Z} }_{c}\right)}^{2}}}^{3}+{\sum }_{p=1}^{{n}_{c}^\mathrm{crs-add}}\frac{1}{3}{{G}_{cp}^\mathrm{M}M}_{c}{\sqrt{{\left(X-{\overline{X} }_{cp}\right)}^{2}+{\left(Y-{\overline{Y} }_{cp}\right)}^{2}+{\left(Z-{\overline{Z} }_{cp}\right)}^{2}}}^{3}$$

and

6 $${M}_{c}={{K}_{1c}}^{2}+{{K}_{2c}}^{2}$$

In Eq. 1, the first term represents the membrane energy generated from the mean curvature $H$ and the Gaussian curvature $G$ that can be expressed with displacement functions $X$, $Y$, and $Z$ in parametric coordinates $u$ and $v$^12,13,16^ (Fig. 1C and Fig. S1 in Supplementary Material (SM)). The second term accounts for energy due to the lateral strain $\alpha$ of membranes^11,14,15^ (Fig. 1C). Details on membrane modeling are described in the APPENDIX and SM. Briefly, ${\alpha }_\mathrm{cross}$ and ${\alpha }_\mathrm{cut}$ are the crossover and cut-off strains, respectively. ${\kappa}$ and ${k}_\mathrm{g}$ are the bending modulus and the Gaussian curvature modulus. ${K}_\mathrm{app}$ is the apparent area stretching modulus. ${c}_{1}$ and ${c}_{2}$ are constants (see APPENDIX). $dA$ is the area element. Throughout this article, all calculations were performed in the range $0\le \alpha \le {\alpha }_\mathrm{cross}$. The Gaussian curvature energy contribution in Eq. 2 was omitted in this work based on the Gauss-Bonnet theorem. A summary of the membrane parameters used in this work is provided in Table S1 in SM.

The expression in the third term of Eq. 1 denotes elastic energies from the crosslinkers that are mobile with the membrane inclusion at one end and fixed in the three-dimensional space at the other end (Fig. 1B and C). The energy density for a group of crosslinkers is written as given in Eq. 5. It gives tensile forces in a quadratic form when both ends of a single crosslinker are fixed (see Eq. 7). $c$ indexes the group of crosslinkers that share the membrane-targeting point i.e., the membrane inclusion (Fig. 1E). $c$ also globally indexes the first crosslinker in each group. It is possible that the group is composed of a single crosslinker (Fig. 1B and C). $p$ indexes additional crosslinkers within the group indexed by $c$. ${n}_\mathrm{crs}$ is the number of crosslinker groups (Fig. 1E). ${n}_{c}^\mathrm{crs-add}$ is the number of additional crosslinkers in the group indexed with $c$. ${\overline{X} }_{c}$, ${\overline{Y} }_{c}$, ${\overline{Z} }_{c}$, ${\overline{X} }_{cp}$, ${\overline{Y} }_{cp}$, and ${\overline{Z} }_{cp}$ are fixed point values for the crosslinkers (red beads in Fig. 1B and C). ${M}_{c}$ and ${{G}_{cp}^\mathrm{M}M}_{c}$ in Eq. 5 are the modulus density functions used to determine the averaged internal elastic energy distribution of the mobile crosslinkers indexed by $c$ and $\left(c,p\right)$, respectively. ${G}_{cp}^\mathrm{M}$ is defined as a unitless gain in this work (see APPENDIX). ${K}_{1c}$ and ${K}_{2c}$ are functions to provide two orthogonal DOFs per each point on the membrane surface. As illustrated in Fig. 1F, by introducing ${K}_{1c}$ and ${K}_{2c}$, the function ${M}_{c}$ (${M}_{c}={{K}_{1c}}^{2}+{{K}_{2c}}^{2}$) along the membrane surface can be defined. Using two ${K}_{1c}$ and ${K}_{2c}$ values as an orthogonal basis, ${{K}_{1c}}^{2}+{{K}_{2c}}^{2}$ defines their intrinsic value greater thanor equal to zero (i.e., squared length). The ${M}_{c}$ value at a certain point on the membrane may provide a combined quantity for the relative residence frequency and the elastic property of the mobile crosslinker. Here, it is not clear whether the elastic properties at different diffusion points are the same or different from each other. ${M}_{c}$ has the unit of Newton per fourth power of meter i.e., $\mathrm{N/{m}^{4}}$. Note that for all calculated solutions in this article, ${K}_{1c}$ was almost equal to ${K}_{2c}$ i.e., ${K}_{1c}\approx {K}_{2c}$. Finally, it is important to note that, in principle, the membrane area with a defined lipid environment needs to be large enough to fully cover the region with modulus density values greater than zero, because discontinuation of the lipid environment contradicts the second principle of this model.

The fourth term of Eq. 1 defines a constraint to preserve the total number of lipids ${n}_\mathrm{lipid}$ by introducing a Lagrange multiplier ${\lambda }^\mathrm{lipid}$. Another Lagrange multiplier ${\lambda }_{c}^\mathrm{crs}$ in the fifth term of Eq. 1 constrains the total value of ${K}_{1c}$ and ${K}_{2c}$ i.e., $\int \left({K}_{1c}+{K}_{2c}\right)dA$, to be an intrinsic constant ${\varphi }_{c}$ (unit: $\sqrt{\mathrm{Newton}}$). ${\varphi }_{c}$ determines the distribution and magnitude of the elastic modulus density, which reflect the confined diffusion area, residence frequency, and elastic property of the mobile crosslinker. Therefore, ${\varphi }_{c}$ is the intrinsic constant that collectively characterizes the elastic property of the crosslinker, as well as the interfacial viscosity between the fluid membrane characterized by a certain lipid environment and the membrane inclusion connected to the elastic crosslinker. Function values for ${K}_{1c}$ and ${K}_{2c}$ can be any real number. Infinitely many possibilities for the shape of the ${M}_{c}$ function with values greater than or equal to zero on the membrane can be made by satisfying Eq. 6 and the condition $\int \left({K}_{1c}+{K}_{2c}\right)dA={\varphi }_{c}$. Among the possibilities, however, the variational analysis gives us one solution profile satisfying the stationary condition of the total energy functional ${\Pi }^\mathrm{tot}$.

It is worth noting that the expressions for the crosslinker are similar to quantum mechanical descriptions. Since the modulus density in this model and the probability density in quantum mechanics are conceptually similar, scaled versions of ${K}_{1c}$, ${K}_{2c}$, and ${M}_{c}$ i.e., $\frac{{K}_{1c}}{\sqrt{{G}_\mathrm{scaling}}}$, $\frac{{K}_{2c}}{\sqrt{{G}_\mathrm{scaling}}}$, and $\frac{{M}_{c}}{{G}_\mathrm{scaling}}$ where ${G}_\mathrm{scaling}$ is the positive scaling factor with the unit of $\mathrm{N/{m}^{2}}$, may correspond to the real part of the wavefunction $\Psi$ ($\mathrm{Re}\left[\Psi \right]$) and the imaginary part of $\Psi$ ($\mathrm{Im}\left[\Psi \right]$), and the probability density i.e., the square of the modulus of $\Psi$ (${\left|\Psi \right|}^{2}$), respectively. This mathematical analogy may confirm our initial modeling objective to account for quantum mechanics for confined Brownian motion. According to the given notions, $\left|\Psi \right|=\sqrt{\frac{{M}_{c}}{{G}_\mathrm{scaling}}}$ can be held.

In this model paradigm, therefore, the wavefunction in quantum mechanics might be alternatively determined by asking the modulus density profile ${M}_{c}$ and the corresponding ${K}_{1c}$ and ${K}_{2c}$ functions. Here, the calculated ${M}_{c}$ defines the combined quantity at each point for the relative residence frequency of a mobile object (the mobile end of a crosslinker in this work) and its energetic attraction property with respect to its conjugate (the fixed end of the crosslinker in this work) with given physical constraints (the membrane deformability in this work) and intrinsic physical constants. Overall, it is required to apply the theory to other quantum mechanical systems such as the particle-in-a-box problem^17,18^ and atomic orbitals to demonstrate its general applicability (see Discussion). It is also worth investigating how solutions of the Schrödinger equation and the presented variational theory of the modulus density are similar to or different from each other. Finally, we might conclude as follows regarding Brownian motion―there is no requirement for the energetic modulus to be a constant value, as in classical mechanics, according to this potentially quantum-compatible modulus density theory. Therefore, Brownian motion in classical mechanics and quantum mechanics are not fully compatible.

Using Eqs. 1‒6, a finite element method was developed, with a full description provided in the APPENDIX and SM. As a quasi-static problem, other time-dependent phenomena beyond confined diffusion—such as membrane viscoelasticity due to prescribed boundaries—are not considered in this model. An analysis of strain gradient generation around membrane-inserted proteins is presented in Fig. S2 (also see SM for discussion on this analysis). Finally, the method was used to investigate: (1) lipid sorting in the deformed membrane; (2) the interaction between the cell membrane and the adhesion proteins integrin and talin; (3) nanomechanical responses of cell membranes; and (4) the correlation of the variational modulus density theory to current theories in classical elasticity.

### Lipid sorting and the formation of lipid nanodomains

**Fig. 2:**
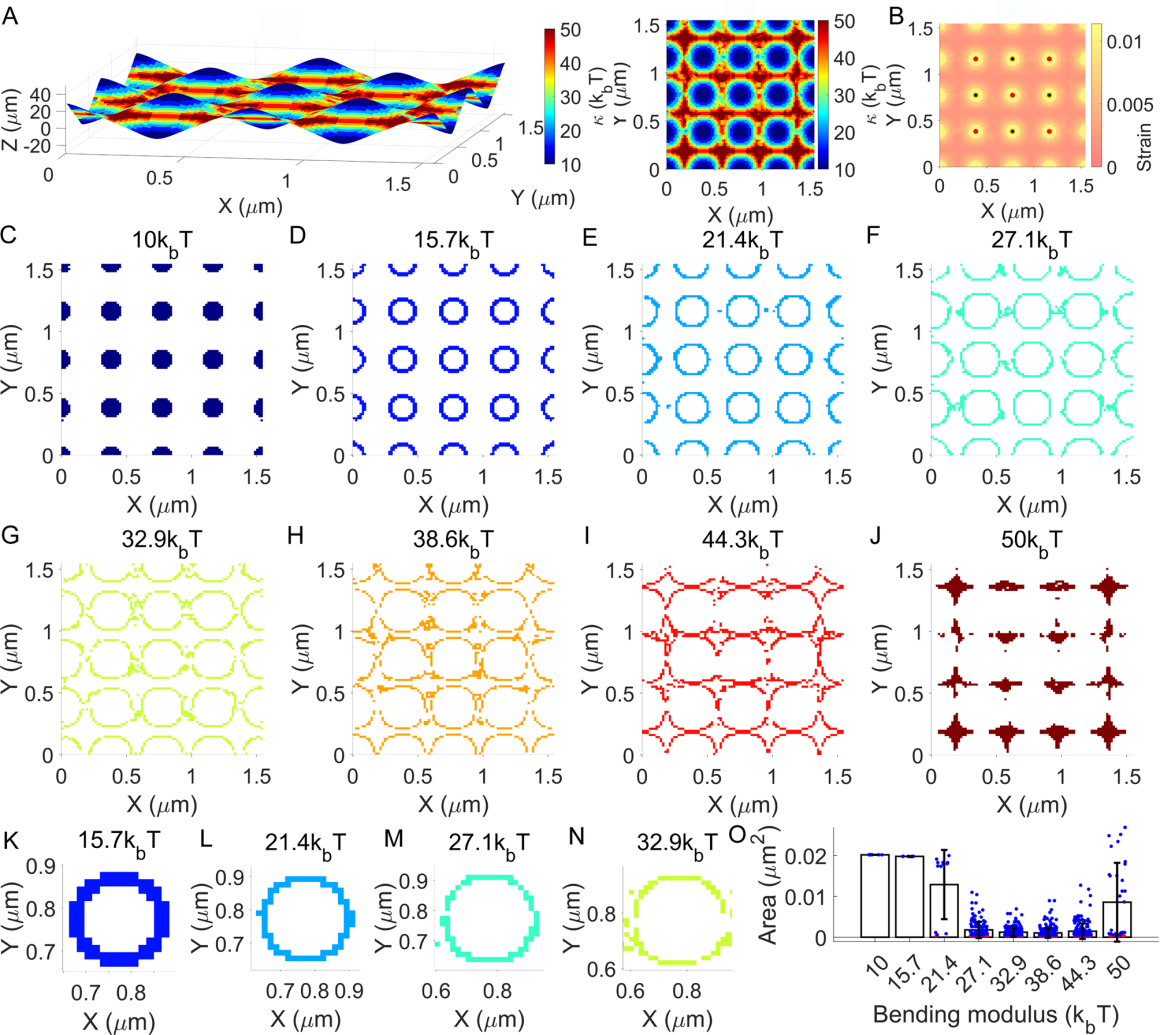
Lipid sorting and formation of lipid nanodomains. (**A**) Three-dimensional deformation of a planar membrane with the surface map for sorting of eight different lipid classes with varying bending moduli. Crosslinkers are not shown. Left: Angled view. Right: Planar view. (**B**) Lipid number strain α profile for the membrane in (A). Crosslinkers are shown here. (**C‒J**) The pattern of lipid nanodomains, shown separately for each lipid class. (**K‒N**) Expanded views of patterns in (D‒G). (**O**) Plots of the mean and standard deviation (SD) of the area of the nanodomains. Mean values are 20,183 ${\mathrm{nm}}^{2}$ (${10k}_\mathrm{b}T$, SD: 11 ${\mathrm{nm}}^{2}$, n: 9), 19,785 ${\mathrm{nm}}^{2}$ (15.7${k}_\mathrm{b}T$, SD: 39 ${\mathrm{nm}}^{2}$, n: 9), 12,897 ${\mathrm{nm}}^{2}$ (21.4${k}_\mathrm{b}T$, SD: 8476 ${\mathrm{nm}}^{2}$, n: 13), 1786 ${\mathrm{nm}}^{2}$ (27.1${k}_\mathrm{b}T$, SD: 1975 ${\mathrm{nm}}^{2}$, n: 146), 1209 ${\mathrm{nm}}^{2}$ (32.9${k}_\mathrm{b}T$, SD: 1117 ${\mathrm{nm}}^{2}$, n: 222), 1060 ${\mathrm{nm}}^{2}$ (38.6${k}_\mathrm{b}T$, SD: 1194 ${\mathrm{nm}}^{2}$, n: 262), 1480 ${\mathrm{nm}}^{2}$ (44.3${k}_\mathrm{b}T$, SD: 1886 ${\mathrm{nm}}^{2}$, n: 194), and 8571 ${\mathrm{nm}}^{2}$ (${50k}_\mathrm{b}T$, SD: 9642 ${\mathrm{nm}}^{2}$, n: 35). Individual domain areas are indicated by colored dots. Blue: Multi-element domain. Red: Single-element domain. See Fig. S14 for the degrees of freedom of each element. See Movie S1 for all membrane-crosslinker configurations used in the analyses in this figure.

In Fig. 2, a ${1.547\times 1.547 \; \mathrm{\mu{m}^{2}}}$ planar membrane and nine regularly defined crosslinkers ($\varphi =2\times {10}^{-5}\sqrt{\mathrm{N}}$) were prepared to investigate lipid sorting due to membrane curvature^19^ and stretching (Fig. 2A and B). The distribution of the crosslinkers was determined by simplifying recent experimental data on CD44^4,20^. Eight lipid classes were assumed in the square membrane (see SM, Lipid sorting simulation). For each lipid class, the total number of lipids was the same, while the bending modulus ${\kappa}$ was different. With systematic mechanical inputs, the eight different lipid classes were sorted, and lipid nanodomains were generated as shown in Fig. 2A and Movie S1 (see SM for details on lipid sorting and segmentation algorithms). The calculated membrane configuration is reminiscent of a mosaic made of different lipid nanodomains as described previously^21^. The lateral strain profile shown in Fig. 2B demonstrates that the lipid domain with a lower bending modulus is more stretched. According to Movie S1, regions with nonzero ${M}_{c}$ values belong to the ${\kappa}={10k}_\mathrm{b}T$ membrane domain.

How the lipid sorting calculation can be correlated to measured compartmentalized diffusion of lipids^22–24^ was also investigated. To this end, the lipid domains with different bending moduli are separately plotted in Fig. 2C‒J. Figure 2C shows the disk-shaped domain, which is similar to the area formed by the confined diffusion of lipids (as an example see Fig. 4B in reference^23^). In Fig. 2F and G (and Fig. 2M and N), donut patterns for the lipid nanodomain are demonstrated. Here, multiple lipid nanodomains collectively form a single donut shape. This donut shape may explain measured hop diffusion trajectories in a closed form (see an example in Fig. 4C of reference^23^). The donut shape with a single lipid domain in Fig. 2D and E (and Fig. 2K and L) might be consistent with the orbital trajectory of the lipid observed from supported lipid bilayers^25^ and living cell membranes^26^. The other patterns in Fig. 2H‒J may explain more randomized hop diffusion trajectories reported in numerous previous experiments with an assumption that a certain lipid can only diffuse to energetically favored lipid nanodomains i.e., the domains with similar lipid parameter values. For a more direct comparison, areas of the nanodomains were calculated and plotted in Fig. 2O. These areas in the length scale of 32.6–142 $\mathrm{nm}$ were similar to measured values for the hop diffusion compartment^27^.

### Mechanical responses of talin and integrins and their interaction with cell membranes in the formation of nascent adhesions

**Fig. 3:**
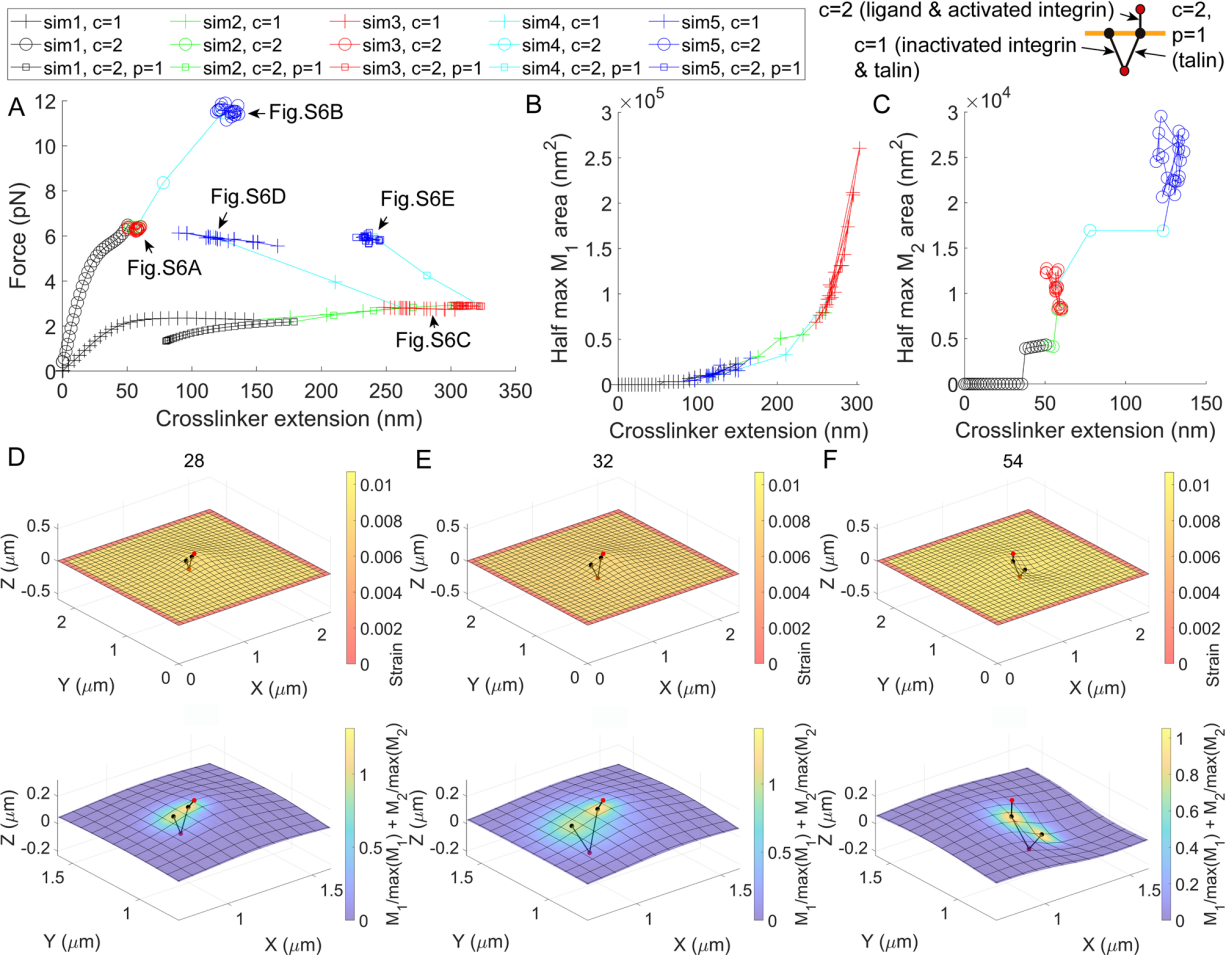
Calculations of the interaction among integrins, talin, and membranes in the formation of nascent adhesions. (**A**) Force vs. extension responses with respect to inputs in Fig. S3. The extension of the crosslinker was defined from its fixed point (red bead) to the maximum ${M}_{c}$ point on the membrane (black bead). Expanded views of indicated regions (arrows) are provided in Fig. S5. (**B, C**) Half-max ${M}_{c}$ area vs. extension responses for the crosslinkers. (**D‒F**) Deformed shapes of the membrane-crosslinker complex. The data point index is indicated at the top. Surface maps indicate the membrane strain $\alpha$ and the sum of normalized elastic modulus densities at the top and bottom, respectively. See Fig. S4 for the membrane-crosslinker complex from a different viewpoint. See Fig. S6 for the surface map of ${M}_{c}$ for $c=1$ and $c=2$. See Fig. S15 for the degrees of freedom. See Movie S2 for all membrane-crosslinker configurations used in the analyses in this figure.

Integrins are focal adhesion receptors that interact with the cytoplasmic adaptor talin^1–3^. To investigate the formation of nascent adhesions mediated by integrins, a $2.32\times 2.32 \; \mathrm{\mu {m}^{2}}$ membrane and two crosslinker groups with a total of three crosslinkers around the center region of the membrane were prepared as a basis complex for adhesion molecular machineries. Here, the crosslinker with $c=1$ represents a talin monomer with an inactivated integrin heterodimer (see inset diagram in Fig. 3, ${\varphi }_{1}=9\times {10}^{-6} \sqrt{\mathrm{N}}$). The crosslinker with $c=2$ represents a complex of a single integrin heterodimer and its extracellular ligand (${\varphi }_{2}=2\times {10}^{-5} \sqrt{\mathrm{N}}$). The crosslinker with $\left(c,p\right)=\left(\mathrm{2,1}\right)$ represents another talin monomer connected to the integrin-ligand complex (${G}_{21}^\mathrm{M}=0.03$). For this additional crosslinker with $\left(c,p\right)=\left(\mathrm{2,1}\right)$, ${\overline{X} }_{cp}={\overline{X} }_{c=1}$, ${\overline{Y} }_{cp}={\overline{Y} }_{c=1}$, and ${\overline{Z} }_{cp}={\overline{Z} }_{c=1}$ were initially defined to simulate a talin dimer whose two monomers share a fixed point on actin cytoskeletons.

Investigations of the integrin-membrane-talin complex consisted of five parts, each with different inputs (Fig. S3 and Fig. 3). The first simulation (sim1, data point index 1‒28), shown with black traces, was performed by pulling the crosslinker with $c=2$. This simulation corresponds to the integrin-ligand interaction during the formation of initial adhesion. The lateral size of membrane tenting was microscopic (Fig. 3D and Fig. S4B). Forces applied to the crosslinker with $c=2$ are plotted in Fig. 3A and Fig. S5A (black circles). Here, the final force, approximately 6.5 pN, was within one significant force population in 5.5–7 pN estimated for the integrin-ligand interaction by using fluorescence resonance energy transfer (FRET) sensors^28^. It is important to note that agreements between FRET force measurements and model calculations here and in the remainder of this paper cannot be used to support the validity of the proposed theory, since the FRET force sensor is based on the conventional mechanics approach (see Discussion). However, the agreements may indicate that molecular systems with fluid membranes exist where both the conventional approach and the new theory work well in estimating applied forces (see Discussion). By using the given ${\varphi }_{1}$, ${\varphi }_{2}$, and ${G}_{21}^\mathrm{M}$ values, forces applied on the talin crosslinkers were 2.3 pN (for $c=1$) and 2.2 pN (for $\left(c,p\right)=\left(\mathrm{2,1}\right)$) at the end of sim1.

The second simulation (sim2, data point index 29‒32), shown with green traces in Fig. S3 and Fig. 3A‒C, was performed by pulling the talin dimer ($c=1$ and $\left(c,p\right)=\left(\mathrm{2,1}\right)$) in the cytoplasmic direction. This pulling of talin dimer during the initial phase of the adhesion may happen with the contraction of actin networks before the formation of stress fibers^29^. Forces applied to talin, ranging from 2.39 to 2.94 pN, were similar to the minimum estimated talin force of about 2.65 pN in a previous FRET experiment^30^. Forces applied to the activated integrin crosslinker remained within the measured FRET force population in 5.5–7 pN (Fig. 3A and Fig. S5A, green circles)^28^.

In the third simulation (sim3, red traces in Fig. S3 and Fig. 3, data point index 33–52), the fixed point for the talin dimer was randomly displaced within an area radially defined by 100 nm from the center. In this part, the average separation between two membrane-targeting ends of the talin dimer was 162.38 nm when projected onto the X–Y plane. This value is consistent with a previous measurement using super-resolution microscopy^31^. The separation was 72.9 nm when the point was randomly displaced within an area defined by 20 nm from the center. The result suggests that the motility of the underlying actin networks is critical for the talin dimer separation. Finally, together with the surface map plots for ${M}_{c}$ (bottom panel of Fig. 3D, E and Fig. S6A, B), the $\int {M}_{c}dA$ plot in Fig. S7 demonstrates that the model finds the solution i.e., the deformed configuration of the membrane-crosslinker complex, by varying both the spatial distribution and the integrated total amount of the elastic modulus density ${M}_{c}$ with given fixed ${\varphi }_{c}$ and ${G}_{cp}^\mathrm{M}$ values.

The fourth simulation (sim4, data point index 53‒54), shown with cyan traces, was performed by increasing ${\varphi }_{1}$ and ${G}_{21}^\mathrm{M}$. Since ${\varphi }_{c}$ is the intrinsic constant, any change in its value may correspond to structural modification of the crosslinker, including its significant refolding, unfolding, and overstretching when the membrane interface remains unchanged. For talin, this can also occur with the change of force-acting sites due to the binding of vinculin along the talin rod^32–34^. The change in ${G}_{cp}^\mathrm{M}$ modifies the relative magnitude of the elastic modulus density of the corresponding crosslinker within its group. By increasing ${\varphi }_{1}$ and ${G}_{21}^\mathrm{M}$, as shown with cyan traces in Fig. S3D and E to mimic the effect of talin fastening with vinculin, the initially tented membrane was contracted under the X–Y plane (Fig. 3F and Fig. S4D).

In the fifth simulation (sim5, data point index 55–74) shown with blue traces, the shared point for the talin dimer was separated, and each monomer was randomly displaced, as shown in Fig. S3A–C. The integrin forces ranged from 11.2 pN to 11.9 pN. The calculations were consistent with previous FRET experimental results—these experiments indicated that vinculin is required for the generation of integrin forces greater than 7 pN^28^. With significant membrane deformation here, the forces on the two talin rods, ranging from 5.5 to 6.2 pN, were also within measured values using the FRET sensors^30,35^. In sim2‒5, the extension values of the two talin monomers were within the experimentally identified range from living cells^36^.

According to a previous study, integrins on living cell surfaces can undergo confined diffusion on the length scale of a hundred or hundreds of nanometers^37^. In the experiment, the radii of the confinement ranged from about 119 nm (inside of the focal adhesion, $4.4488\times {10}^{4} \; \mathrm{{nm}^{2}}$) to 236 nm (outside of the focal adhesion, $1.7497\times {10}^{5} \; \mathrm{{nm}^{2}}$) (see Fig. 1i in the reference paper^37^). Furthermore, a recent experiment directly visualized that the initially freely mobile integrin was confined in a similar length scale with the talin colocalization^38^. This confined diffusion area was about $1.102\times {10}^{5} \; \mathrm{{nm}^{2}}$ (Fig. 2A right in the reference paper^38^; see SM for the area calculation)^38^. These observations support the crosslinker mechanism for the confined integrin diffusion. Remarkably, areas defined by half of the maximum ${M}_{c}$ value in Fig. 3B and C were consistent with the confined integrin diffusion area observed in the experiments^37,38^ (see SM for the calculation of the half-max ${M}_{c}$ area).

Stretching and sliding of crosslinkers with membrane deformation are visualized in Movie S2 for all data in Fig. 3. In Figs. S8–10 and Movie S3, molecular machineries with four adhesion sites are investigated by using the same membrane and parameter values. The results were largely consistent with those from Fig. 3. Overall, the presented analyses demonstrate the remarkable ability of the model to predict biological responses in the formation of nascent adhesion at the single-molecule level.

### The effect of the number and distribution of crosslinkers, as well as the composition of lipids, on nanomechanical responses of membrane-crosslinker complexes

**Fig. 4:**
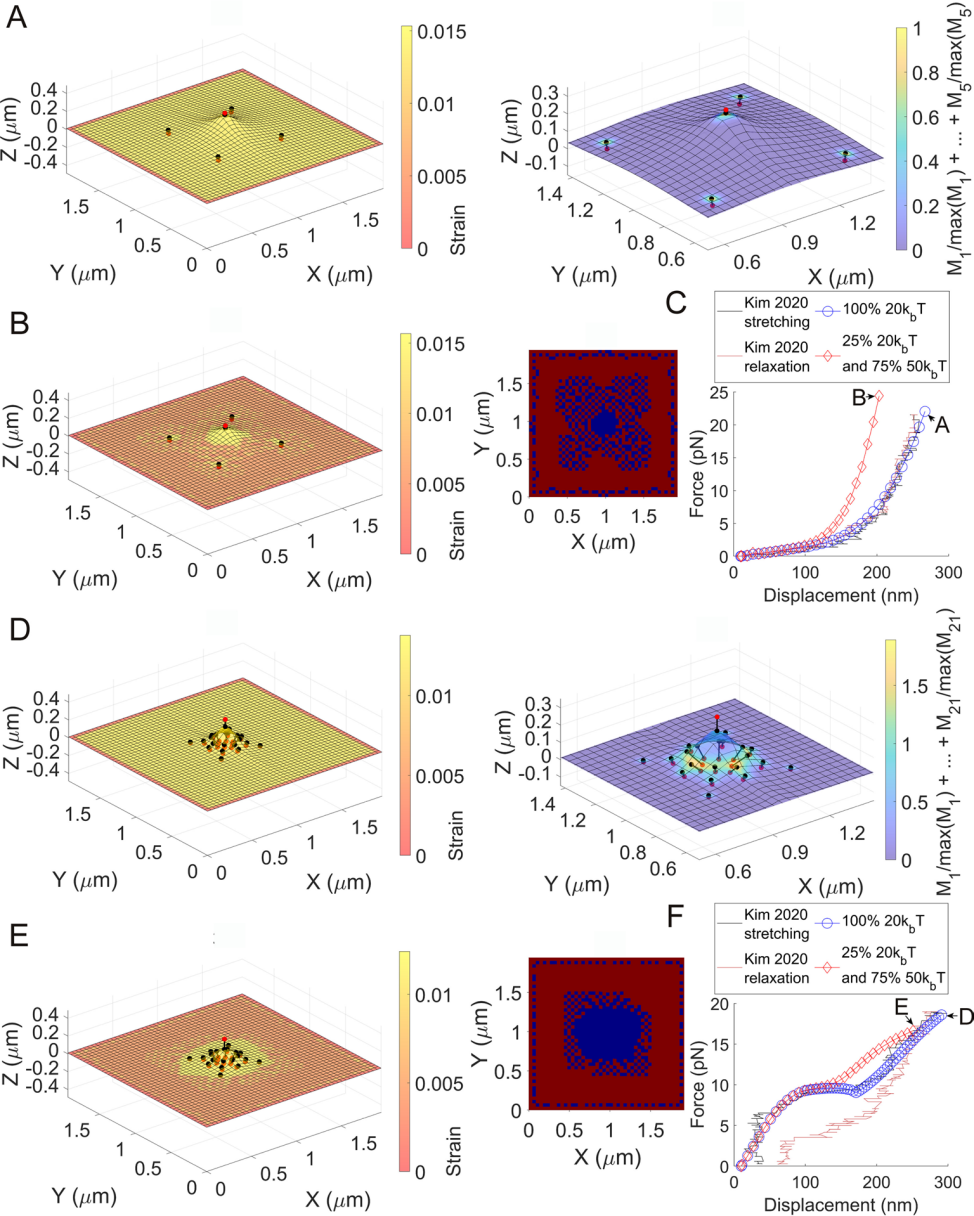
The effect of the number and distribution of crosslinkers and lipid composition on nanomechanical responses of membrane-crosslinker complexes. (**A**) The deformed configuration with the surface strain map for a $1.944\times 1.944 \; \mathrm{\mu {m}^{2}}$ membrane (${\kappa}=20{k}_\mathrm{b}T$) and five crosslinkers when the center crosslinker ($c=3$) was pulled with a 249 nm displacement (left). An expanded view of the membrane-crosslinker complex with the surface map for the sum of normalized ${M}_{c}$ values (right). (**B**) A deformed configuration with the surface strain map for the membrane-crosslinker complex in (A) by considering 75% $50{k}_\mathrm{b}T$ lipids and 25% $20{k}_\mathrm{b}T$ lipids. The corresponding lipid sorting map is shown on the right. (**C**) The force vs. displacement of the red bead at the center ($c=3$) in (A) is shown with blue circles, and compared to experimental data^11^. The force vs. displacement for the center red bead in (B) is shown with red diamonds. (**D**) The deformed configuration with the surface strain map for the $1.944\times 1.944 \; \mathrm{\mu {m}^{2}}$ membrane (${\kappa}=20{k}_\mathrm{b}T$) and twenty-one crosslinkers when the center crosslinker ($c=11$) was pulled with a 281 nm displacement (left). An expanded view of the membrane-crosslinker complex with the surface map for the sum of normalized ${M}_{c}$ values (right). (**E**) A deformed configuration with the surface strain map for the membrane-crosslinker complex in (D) by considering 75% $50{k}_\mathrm{b}T$ lipids and 25% $20{k}_\mathrm{b}T$ lipids. The corresponding lipid sorting map is shown on the right. (**F**) The force vs. displacement of the red bead at the center ($c=11$) in (D) is shown with blue circles and compared to experimental data^11^. The force vs. displacement for the center red bead in (E) is shown with red diamonds. See Figs. S16 and S17 for the degrees of freedom. Movies S4‒S7 visualize all membrane-crosslinker complexes used in the analyses in this figure.

To reproduce recently identified nanomechanical responses of cell membranes^11^, five crosslinkers were defined in a $1.944\times 1.944 \; \mathrm{\mu {m}^{2}}$ membrane where the distance from one at the center to the other four crosslinkers is about 488.8 nm (Fig. 4A, Fig. S11A, and Movie S4). The ${\varphi }_{c}$ value for the crosslinker located at the center ($c=3$) was $2.6\times {10}^{-5}\sqrt{\mathrm{N}}$. ${\varphi }_{c}$ for the other crosslinkers ($\mathrm{c}=1, 2, 4, 5$) that represent the membrane-cytoskeleton linkage was $6.5\times {10}^{-6}\sqrt{\mathrm{N}}$. This value was selected to investigate whether $\varphi$ smaller than that of the full-length talin monomer (with the inactivated integrin) can generate the nanomechanical responses. To calculate the force vs. displacement response, the ${\overline{Z} }_{c}$ value for the center crosslinker ($c=3$) was increased. The force vs. displacement curve shape in Fig. 4C (blue circles) showed good agreement with that of a measured nanomechanical response of cell membranes^11^.

To reproduce another type of nanomechanical response, twenty-one crosslinkers were compactly inserted in a region spanning 305.5 nm from the center of the $1.944\times 1.944 \; \mathrm{{\mu {m}}^{2}}$ membrane (see Fig. 4D, Fig. S11B, and Movie S6). The shortest distance from the center to a crosslinker was 61.1 nm (Fig. S11B). ${\varphi }_{c}=1.6\times {10}^{-5}\sqrt{\mathrm{N}}$ for the centered crosslinker ($\mathrm{c}=11$), while ${\varphi }_{c}=6.5\times {10}^{-6}\sqrt{\mathrm{N}}$ for the others were used. Increasing the number of crosslinkers within a reduced area resulted in a more localized deformation shape of the membrane. The crescent shape of the area for the elastic modulus density ${M}_{c}$ around the sharp membrane curvature ($\mathrm{c}=7, 8, 14, 15$) demonstrates the mechanistic and hydrostatic interaction between the fluid membrane and the crosslinkers connected to mobile membrane inclusions (Fig. S12). With the given twenty-one crosslinkers, the force vs. displacement response showed zero stiffness at an intermediate displacement (Fig. 4F, blue circles). A comparison between the calculated zero stiffness response and experimental data from a previous work also showed good agreement (Fig. 4F, blue circles)^11^. According to Fig. S13 and Movie S8, reducing the distance to the membrane-cytoskeleton linker from the center, without a sufficient increase in the number of crosslinkers, can result in instabilities that break the symmetry of the deformation of the membrane-crosslinker complex.

The effect of lipid composition on the generation of the nanomechanical response was also investigated. The force vs. displacement responses with five and twenty-one crosslinkers were reexamined using two different lipids (${\kappa}=50{k}_\mathrm{b}T$ and ${\kappa}=20{k}_\mathrm{b}T$) in Fig. 4C and F, respectively (red diamonds). For simplicity, the same ${\varphi }_{c}$ values in Fig. 4A and D were used for the five and twenty-one crosslinkers, respectively, in the calculations with the two lipid classes, even though the lipid environment here is not exactly the same as that of Fig. 4A and D. With mechanical pulling of the center protein, two lipid classes were sorted and patterns were generated in the membrane in both cases, as shown in Movies S5 and S7. The central region with high membrane curvatures was filled with the lipid of ${\kappa}=20{k}_\mathrm{b}T$ for both cases. Unlike the sorting investigation in Fig. 2, the collapse of the boundary between two lipid domains was identified as the lateral membrane stretching became significant with larger displacements (Fig. 4B and E; Movies S5 and S7). Notably, for both cases, the response in the high displacement region shifted when the lipid of ${\kappa}=50{k}_\mathrm{b}T$ was considered alongside the $20{k}_\mathrm{b}T$ lipid (Fig. 4C and F). This shifting effect is consistent with the effect of reducing the size of lipid reservoirs in mechanical stretching of membranes, as analyzed in the previous work^11^. According to the presented analysis, therefore, the reduced size of lipid reservoirs in membrane stretching is a result of the presence of different lipid classes with higher bending moduli and sorting of the lipids in the deformed membrane.

### The correlation of the variational modulus density theory to current theories in classical elasticity

**Fig. 5:**
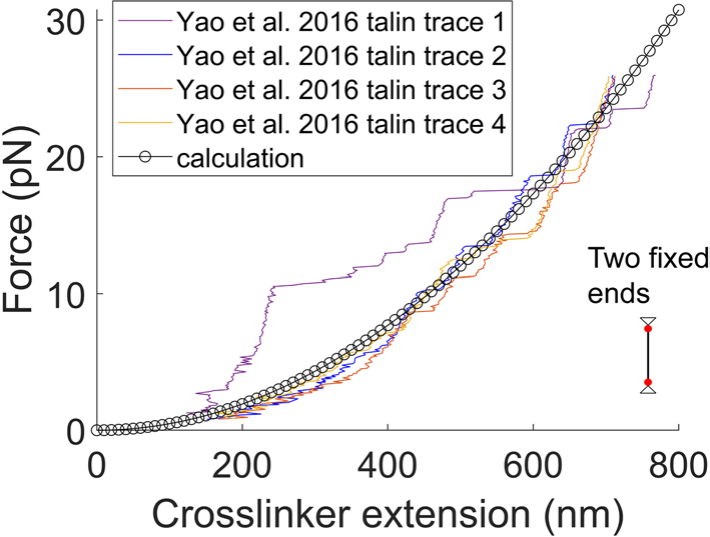
Application of the modulus density theory to the crosslinker with two fixed ends. Calculations using Eq. 7 with ${\varphi }_\mathrm{fixed-talin}=1\times {10}^{-7}\sqrt{\mathrm{N}}$ and ${A}_{\mathrm{talin}}=10.2 \; \mathrm{{nm}^{2}}$ (circles). Both ends of the crosslinker are assumed fixed. Calculations are compared with magnetic tweezer measurements for single full-length talin monomers interacting with vinculin molecules in solution^31^. The calculations and measurements show reasonable agreement.

How the two principles in the modulus density theory can be applied to the crosslinker with two fixed ends was investigated by using previous magnetic tweezer data on a single monomeric talin^31^. In Fig. 5, the experimental tensile force vs. extension data, obtained from single full-length talin monomers fixed at both ends (one at the magnetic bead and the other at the substrate), and interacting with vinculin molecules, were compared to a calculated curve using the following Eq. 7:

7 $${{f}}_\mathrm{fixed-talin}=\frac{{{\varphi }_\mathrm{fixed-talin}}^{2}{{\Delta {L}}_{\mathrm{talin}}}^{2}}{2{{A}}_{\mathrm{talin}}}$$

where ${\Delta L}_\mathrm{talin}$ and ${A}_\mathrm{talin}$ are the talin extension and the coarse-grained cross-section area for talin, respectively. The two principles, described in the Introduction section with respect to the mobile crosslinker, are applied in deriving Eq. 7 as follows. First, the force for talin in a fixed configuration can be calculated by finding the modulus density value used to calculate the internal elastic energy of talin. Here, the statistical description in the first principle for the mobile crosslinker is conceptually simplified for the fixed crosslinker. Second, by assuming that the response of the fixed talin is also governed by its intrinsic physical constant termed ${\varphi }_\mathrm{fixed-talin}$, all possible values for the modulus density are derived from ${\varphi }_\mathrm{fixed-talin}$, where the single modulus density value that generates the minimum energy is selected to calculate the talin force. In mathematics, which is employed to describe natural phenomena, decomposition (or addition) is the most fundamental operation. Accordingly, as the best way, ${\varphi }_\mathrm{fixed-talin}$ is decomposed to generate multiple variations of the modulus density value. As similarly done for the mobile crosslinker, using two variables to decompose ${\varphi }_\mathrm{fixed-talin}$ might be enough to maximize the size of the set of possible values (see APPENDIX for the derivation of Eq. 7). The ${\varphi }_\mathrm{fixed-talin}$ value was $1\times {10}^{-7 } \sqrt{\mathrm{N}}$ when ${A}_{\mathrm{talin}}=10.2 \; \mathrm{{nm}^{2}}$ in an extension range of about 0–800 nm (Fig. 5). It is important to note that the nature of ${\varphi }_\mathrm{fixed-talin}$ and $\varphi$ for the mobile crosslinker are different from each other, as the concept of the interface viscosity is absent in ${\varphi }_\mathrm{fixed-talin}$.

Classical mechanics laws such as Hooke’s law and nonlinear elasticity state that the force on a one-dimensional elastic spring is proportional to its extension (or a function of the extension), through a modulus such as Hooke’s spring constant or Young’s modulus. The formalism of the variational modulus density theory for the two-fixed-ends crosslinker is aligned with the notion of those conventional theories. According to the derivation of Eq. 7, the intrinsic constant ${\varphi }_\mathrm{fixed-talin}$ yields the constant elastic modulus value under the minimum energy assumption (see APPENDIX for the derivation of Eq. 7). Overall, the analysis in Fig. 5 suggests that the principles of the modulus density theory are identically applicable to the elastic linker with two fixed ends.

**Fig. 6:**
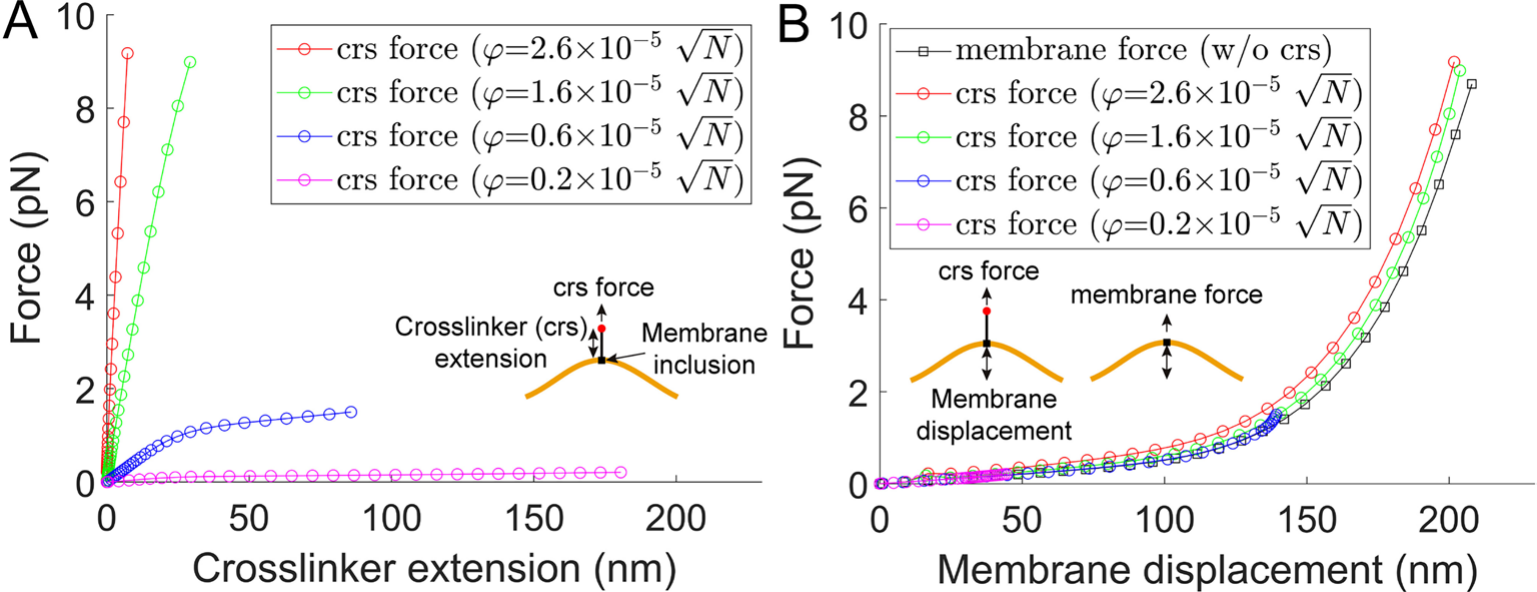
A combined analysis for the force transfer at the membrane-crosslinker interface using the modulus density theory. (**A**) The $1.944\times 1.944 \; \mathrm{{\mu m}^{2}}$ membrane was stimulated by using four different complexes of crosslinkers and membrane inclusions ($\varphi = 2.6\times {10}^{-5}\sqrt{\mathrm{N}}$, $\varphi = 1.6\times {10}^{-5}\sqrt{\mathrm{N}}$, $\varphi = 0.6\times {10}^{-5}\sqrt{\mathrm{N}}$, and $\varphi = 0.2\times {10}^{-5}\sqrt{\mathrm{N}}$). Forces applied to the crosslinkers were plotted against the extension of the crosslinkers. The four responses vary significantly. (**B**) The forces on the crosslinkers in (**A**) were plotted against the displacement of the membrane. Here, the response of pulling the membrane without the center crosslinker was additionally plotted (black empty squares). The responses overlap with small variations. See Fig. S18 for the degrees of freedom.

In Fig. 6, a $1.944\times 1.944 \; \mathrm{{\mu m}^{2}}$ membrane with a single crosslinker at its center was used to further investigate the characteristics of the crosslinker interacting with the fluid membrane. With four different $\varphi$ values, the responses of the crosslinker varied significantly (Fig. 6A). In this model framework, a single $\varphi$ value collectively defines both the elastic property of the crosslinker and the viscosity of the membrane inclusion connected to it. Therefore, such non-negligible variations may be generated among identical crosslinkers if their inclusion properties are significantly different. This suggests that estimating the crosslinker force (or energy) from its extension using conventional elasticity might not always be valid when the crosslinker interacts with the fluid membrane.

According to Fig. 6B, however, the crosslinker force vs. membrane displacement responses overlapped for all $\varphi$ values used. They were also consistent with the membrane force response directly obtained by taking the derivative of the membrane energy without considering the pulling crosslinker (black empty squares in Fig. 6B). While the validity of Hooke’s law for the crosslinker under confined Brownian diffusion is elusive, the results in Fig. 6B suggest that Newton’s third law is still valid in this new paradigm, specifically at the membrane-crosslinker interface. Therefore, the membrane deformation can be used to approximate the force on the crosslinker, regardless of its elastic property and membrane-inserted interface, as similarly conducted in previous works^10,11,39,40^. Small variations among the five curves in Fig. 6B might be generated numerically or due to differences in modulus density profiles that can minimally influence the curvature of membranes at their tips. Overall, the results in Figs. 5 and 6 suggest that the presented variational modulus density theory provides a more general framework for explaining one-dimensional elastic linkers not only interacting with the fluid membrane but also in standalone configurations. Finally, the results emphasize the importance of characterizing the $\varphi$ value for various complexes of crosslinkers and their membrane-inserted molecules.

## Discussion

There is a viewpoint that considers Brownian diffusion as a quantum phenomenon^6–8^, while membrane deformation has been explained by classical continuum mechanics^9–11^. To explain confined Brownian diffusion in the membrane, the main modeling objective in this work was to develop a new theory in which quantum mechanical descriptions can be meaningfully reflected in the classical mechanics framework. To this end, the variational modulus density theory, incorporating two novel principles, was developed. The principles can be summarized as follows: (1) the shared value for a certain physical characteristic across multiple bodies is equal to the value of the characteristic in their average configuration; and (2) all ensemble averages of confined physical motions of an object might be mathematically predictable with an assumption that the motions are governed by an intrinsic physical constant. This new modeling approach, for the confined Brownian motion and mechanical deformation of crosslinkers interacting with deforming cell membranes, establishes a mathematical analogy between the proposed theory and the quantum mechanical wavefunction.

For a crosslinker interacting with a solid membrane, a one-dimensional force based on conventional elasticity can be used to represent the crosslinker force at a specific point on the membrane (corresponding to Fig. 1A, left). However, this approach may not be directly applicable to a fluid membrane, which differs fundamentally from a solid one. As a modeling strategy in this study, the crosslinker force was calculated by averaging over multiple configurations of the mobile crosslinker (Fig. 1A, right). In this new paradigm, it is no longer necessary to assume a constant elastic modulus as in the conventional theory. Instead, the force calculation relies on the averaged configuration of the mobile crosslinker, rather than individual configurations. Assuming that the crosslinker force remains constant during confined diffusion, it is reasonable to equate the force applied by each individual configuration with that of the averaged configuration. To represent this averaged behavior, the concept of elastic modulus density was introduced.

A mathematical framework to maximize the variability of the shape of the modulus density function $M$ made from the intrinsic constant $\varphi$ is also proposed. In this framework, it is necessary to use the integral summation to correlate the constant $\varphi$ with the two-dimensional functions ${K}_{1}$ and ${K}_{2}$. At each point on the membrane surface, two function values from ${K}_{1}$ and ${K}_{2}$ define the modulus density value. By doing so, the “plus” and “minus” operations (i.e., two fundamental operators in summation) can be independently considered at each point in the integration process leading to the constant $\varphi$. In other words, employing the two degrees of freedom (DOFs) allows the most dynamic decomposition of the single constant $\varphi$ into real numbers distributed across a continuous area. Consequently, this maximizes the variability of the resulting modulus density profile derived from the constant $\varphi$. More precisely, all physically possible confined diffusion configurations for a certain membrane inclusion, linked to a certain crosslinker and interacting with a certain lipid environment, might be generated using this mathematical framework, with an assumption that the physical motion of the inclusion-crosslinker complex is governed by its intrinsic constant. The functions ${K}_{1}$ and ${K}_{2}$ may correspond to the real and imaginary parts of the wavefunction in quantum mechanics. How the mathematical identification for the “positive” and “negative” components of the modulus density value at each point can be interpreted in a more physical manner is an additional aspect to be explored in the future.

The consistency of the calculated results with experimental data suggests that the mobile and force-bearing crosslinker may be better explained using this new approach. Most notably, the area defined by half of the maximum $M$ value for the force-bearing talin was similar to the measured area for the integrin under confined diffusion^37,38^. Additionally, single-molecule magnetic tweezer responses for talin were reproduced^31^. The separation distance between two membrane-targeting ends of the talin dimer was consistent with a previous experiment^31^. Also, the model directly reproduced measured nonlinear nanomechanical responses of living cell membranes^11^. These agreements collectively support the validity of the model.

The possibility of generating different force values for the same crosslinker with the same extension but with different membrane interfaces may question the validity of FRET force sensors interacting with the fluid membrane, because the technology parameterizes the extension of sensor molecules to estimate the force, as in the conventional mechanics theory^28,30,35^. In this work, calculated forces for talin were similar to FRET measurements from living cells^30,35^. Calculated forces for integrin-ligand interactions at adhesions were also consistent with experimental FRET data^28^. Assuming that the new theory is reasonable, these agreements suggest that the conventional mechanics approach and the corresponding FRET sensor might be effective in determining forces on various membrane-associated proteins such as talin and the integrin. Nevertheless, it is still unclear whether the conventional approach can generate the same confined diffusion area. Overall, more direct experimental investigations into the validity of the FRET force sensor with respect to many other membrane protein systems and lipid environments might be required.

One particular focus in this work was the sorting of lipids and the generation of lipid nanodomains due to membrane curvatures and stretching. The size and shape of the nanodomains identified in this work were similar to those of membrane compartments identified by tracking the diffusion of lipids in living cell membranes. The picket-fence model suggests that anchored transmembrane proteins serve as physical barriers against the diffusion of lipids^22,41^. However, recently identified CD44 proteins do not fully fence a closed area in the membrane while they are responsible for membrane compartmentalization^4,20^. The result may ask an alternative explanation in which the compartment can be made with few picket proteins. The presented analyses suggest that lipids can diffuse only into energetically favored nanodomains i.e., the domains with the same lipid parameter values, and that might be responsible for the generation of the observed compartmentalized diffusion. In this alternative paradigm, CD44 simply serves as the membrane-cytoskeleton crosslinker to generate membrane curvatures and stretching. The presented interpretation can also explain the hop diffusion observed in the supported lipid bilayer where picket-fence proteins are absent^25,42,43^. The lipid sorting study in this work further explains how the presence of different lipid classes reduces the size of lipid reservoirs in mechanical deformation of cell membranes^11,44–46^. Overall, the work demonstrated that the effect of molecular composition in lipid membranes can be analyzed in detail within the framework of continuum mechanics, as actively investigated recently for various problems including vesicle budding with lipid demixing^47^ and the interaction between amphipathic peptides and lipid membranes^48,49^.

As mentioned earlier, the demonstration of the general applicability of the modulus density theory to other physical systems might be important. In this regard, investigating atomic orbitals using the theory would be intriguing. The electron is mobile in three-dimensional space and is energetically attracted to the nucleus. Additionally, multiple electrons can physically constrain each other’s motion due to mutual repulsion. Moreover, the kinetic energy of electrons can push them away from the nucleus. Therefore, the physical and mathematical characteristics of the atomic orbital may share similarities with those of the membrane-crosslinker complex examined in this work (though the kinetic energy is not considered in this work; see SM for an estimate for the kinetic energy of confined diffusion). The electron cloud in the conventional model could be viewed as a volume characterized by the modulus density value for the electron-nucleus interaction in this model paradigm. Likewise, the presented theory may be applicable to modeling various flow phenomena, such as those involving liquids, information, or currency. Additionally, conventional theories based on spring-like, one-dimensional representations for the interaction between two fluid particles—such as molecular fluid dynamic models—may need to be reformulated based on this work. Finally, investigating planetary trajectories and artificial intelligence through the lens of this theory and its underlying principles could open up fascinating extensions of the presented research.

In summary, the area of the confined Brownian motion for force-bearing crosslinkers was explored by introducing a continuum theory that is potentially compatible with the quantum wavefunction. The variational modulus density theory for crosslinkers was developed and integrated into the continuum model for cell membranes. A novel finite element method for the combined model was developed, which not only reproduced numerous experimental data but also provided valuable predictions regarding the lateral organization of the cell membrane and the membrane-crosslinker interaction. The model also suggested potential answers for the physical and mathematical identity of the quantum wavefunction. The theoretical model presented in this work is expected to provide enhanced capabilities for the realistic simulation of cell membranes and membrane crosslinkers.

### Footnote

All figure data, as well as the initial version of the manuscript and the Supplementary Material, were generated at INTEGRITY Co.,Ltd. (9, Gangnamseo-ro, Giheung-gu, Yongin-si, Gyeonggi-do, Republic of Korea, 16977), which is no longer active.

## Electronic supplementary material

Below is the link to the electronic supplementary material.

Supplementary Information 1.

Supplementary Video 1.

Supplementary Video 2.

Supplementary Video 3.

Supplementary Video 4.

Supplementary Video 5.

Supplementary Video 6.

Supplementary Video 7.

Supplementary Video 8.

## Data Availability

Calculated raw data are available from 10.6084/m9.figshare.28089794

## Appendix

### Descriptions for the membrane model

In Eq. 2 of the Results section, the mean and Gaussian curvatures at a given point on the membrane surface can be expressed as $H=\frac{\left(1+{{Z}_{,X}}^{2}\right){Z}_{,YY}-2{Z}_{,X}{Z}_{,Y}{Z}_{,XY}+\left(1+{{Z}_{,Y}}^{2}\right){Z}_{,XX}}{2{\left(1+{{Z}_{,X}}^{2}+{{Z}_{,Y}}^{2}\right)}^{3/2}}$ and $G=\frac{\left({Z}_{,XX}{Z}_{,YY}-{{Z}_{,XY}}^{2}\right)}{{\left(1+{{Z}_{,X}}^{2}+{{Z}_{,Y}}^{2}\right)}^{2}}$, respectively^16^. Here $X$, $Y$, and $Z$ are displacement functions for the membrane in the three-dimensional space (Fig. 1 and Fig. S1). ${Z}_{,X}$ and ${Z}_{,Y}$ represent derivatives of $Z$ with respect to $X$ and $Y$, respectively. Similarly, ${Z}_{,XX}$, ${Z}_{,XY}$, and ${Z}_{,YY}$ are derivatives of ${Z}_{,X}$, ${Z}_{,X}$, and ${Z}_{,Y}$ with respect to $X$, $Y$, and $Y$, respectively. The area element $dA$ can be expressed as $dA=\Vert \widehat{n}\Vert dudv=\sqrt{{{\widehat{n}}_\mathrm{X}}^{2}+{{\widehat{n}}_\mathrm{Y}}^{2}+{{\widehat{n}}_\mathrm{Z}}^{2}}dudv$, where ${\widehat{n}}_\mathrm{X}={Y}_{,u}{Z}_{,v}-{Z}_{,u}{Y}_{,v}$, ${\widehat{n}}_\mathrm{Y}={Z}_{,u}{X}_{,v}-{X}_{,u}{Z}_{,v}$, and ${\widehat{n}}_\mathrm{Z}={X}_{,u}{Y}_{,v}-{Y}_{,u}{X}_{,v}$. $\widehat{n}=\left({\widehat{n}}_\mathrm{X}, {\widehat{n}}_\mathrm{Y}, {\widehat{n}}_\mathrm{Z}\right)$ is the normal vector at a given point on the membrane surface. $u$ and $v$ are parametric coordinates defined on the membrane surface (Fig. 1C and Fig. S1). Parametric derivatives for $X$, $Y$, and $Z$ used for the expansion of Eq. 1 are summarized in Eqs. S1‒S30 in Supplementary Material (SM).

${E}^\mathrm{st}$ in Eqs. 3, 4 defines the strain energy density of the area $dA$. The equations were derived from two continuous expressions for the surface tension, $\sigma ={\sigma }_{0}\mathrm{exp}\left(\frac{8\pi {\kappa}\alpha }{{k}_\mathrm{b}T}\right)$ for $0\le \alpha \le {\alpha }_\mathrm{cross}$ and ${\sigma =K}_\mathrm{app}\left(\alpha -{\alpha }_\mathrm{cut}\right)$ for $\alpha>{\alpha }_\mathrm{cross}$^11,50^. Here, $\alpha$ represents the lateral strain. The parameters ${\alpha }_\mathrm{cut}$ and ${\alpha }_\mathrm{cross}$ are the cut-off and crossover strains, respectively, determined by enforcing continuity between the two surface tension expressions. ${\sigma }_{0}$ is the surface tension at zero strain. ${K}_\mathrm{app}$ is the apparent area stretching modulus, and ${K}_\mathrm{app}=\left\{0.1675{\left(\frac{{\kappa}}{{k}_\mathrm{b}T}\right)}^{2}-1.972\left(\frac{{\kappa}}{{k}_\mathrm{b}T}\right)+166.1\right\}{10}^{-3}$ was used to define the value^50^. ${k}_\mathrm{b}$ is the Boltzmann constant and $T$ is the temperature in Kelvin. The constants ${c}_{1}$ and ${c}_{2}$ in Eqs. 3 and 4 are ${c}_{1}=\frac{8\pi {\kappa}}{{k}_\mathrm{b}T}$ and ${c}_{2}=\frac{{\sigma }_{0}}{{c}_{1}}\mathrm{exp}\left({c}_{1}{\alpha }_\mathrm{cross}\right)-\frac{{\sigma }_{0}}{{c}_{1}}-\left(\frac{{K}_\mathrm{app}}{2}{\alpha }_\mathrm{cross}^{2}-{K}_\mathrm{app}{\alpha }_\mathrm{cut}{\alpha }_\mathrm{cross}\right)$, respectively^11,50^. ${\kappa}$ is the bending modulus of the membrane used in the range $0\le \alpha \le {\alpha }_\mathrm{cross}$. Throughout this article, all calculations were conducted within this strain range, since the bending energy expression in Eq. 2 may not remain valid for $\alpha>{\alpha }_\mathrm{cross}$^50^.

## Finite element methods for membrane-crosslinker complexes

### Variational equations for finite element modeling

The equilibrium shape of the membrane-crosslinker complex can be determined by setting the variation of the given functional in Eq. 1 as being equal to zero. To achieve this, Eq. 1 can be rewritten with respect to the parametric coordinates $u$ and $v$, as shown in Eqs. 8‒13.

8 $$\begin{aligned} \Pi ^{\mathrm{tot}} = & \int {{\mathcal{F}}^{\mathrm{curv}} } \left( {X_{{,u}} ,X_{{,v}} ,X_{{,uu}} ,X_{{,vv}} ,X_{{,uv}} ,X_{{,vu}} ,Y_{{,u}} ,Y_{{,v}} ,Y_{{,uu}} ,Y_{{,vv}} ,Y_{{,uv}} ,Y_{{,vu}} ,Z_{{,u}} ,Z_{{,v}} ,Z_{{,uu}} ,Z_{{,vv}} ,Z_{{,uv}} ,Z_{{,vu}} } \right)dudv \\ & + \int {{\mathcal{F}}^{\mathrm{st}} } \left( {X_{{,u}} ,X_{{,v}} ,Y_{{,u}} ,Y_{{,v}} ,Z_{{,u}} ,Z_{{,v}} ,\alpha } \right)dudv \\ & + \int {\sum\limits_{{c = 1}}^{{n_{\mathrm{crs}} }} {{\mathcal{F}}_{c}^{\mathrm{crs}} } } \left( {X,X_{{,u}} ,X_{{,v}} ,Y,Y_{{,u}} ,Y_{{,v}} ,Z,Z_{{,u}} ,Z_{{,v}} ,K_{{1c}} ,K_{{2c}} } \right)dudv \\ & + \int {{\mathcal{F}}^{\mathrm{lipid - cstr}} } \left( {X_{{,u}} ,X_{{,v}} ,Y_{{,u}} ,Y_{{,v}} ,Z_{{,u}} ,Z_{{,v}} ,\alpha ,\lambda ^{\mathrm{lipid}} } \right)dudv - \lambda ^{\mathrm{lipid}} n_{\mathrm{lipid}} \\ & + \sum\limits_{{c = 1}}^{{n_{\mathrm{crs}} }} {\left( {\int {{\mathcal{F}}_{c}^{\mathrm{crs-cstr}} } \left( {X_{{,u}} ,X_{{,v}} ,Y_{{,u}} ,Y_{{,v}} ,Z_{{,u}} ,Z_{{,v}} ,K_{{1c}} ,K_{{2c}} ,\lambda _{c}^{\mathrm{crs}} } \right)dudv - \lambda _{c}^{\mathrm{crs}} \varphi _{c} } \right)} \\ \end{aligned}$$

where

9 $${\mathcal{F}}^\mathrm{curv}={E}^\mathrm{curv}\Vert \widehat{n}\Vert$$

10 $${\mathcal{F}}^\mathrm{st}={E}^\mathrm{st}\Vert \widehat{n}\Vert$$

11 $${\mathcal{F}}_{c}^\mathrm{crs}={E}_{c}^\mathrm{crs}\Vert \widehat{n}\Vert$$

12 $${\mathcal{F}}^\mathrm{lipid-cstr }={\lambda }^\mathrm{lipid}\frac{{\phi }_{0}}{\alpha +1}\Vert \widehat{n}\Vert$$

and

13 $${\mathcal{F}}_{c}^\mathrm{crs-cstr}={\lambda }_{c}^\mathrm{crs}\left({K}_{1c}+{K}_{2c}\right)\Vert \widehat{n}\Vert$$

The variation of the functional i.e., ${\delta \Pi }^\mathrm{tot}$, can then be written as follows.

$$\delta {\Pi }^\mathrm{tot}=\int {\left[\begin{array}{c}\partial {\mathcal{F}}^\mathrm{membr-all }/\partial {X}_{,u}\\ \begin{array}{c}\partial {\mathcal{F}}^\mathrm{membr-all }/\partial {X}_{,v}\\ \partial {\mathcal{F}}^\mathrm{curv}/\partial {X}_{,uu}\\ \begin{array}{c}\partial {\mathcal{F}}^\mathrm{curv}/\partial {X}_{,vv}\\ \partial {\mathcal{F}}^\mathrm{curv}/\partial {X}_{,uv}\\ \begin{array}{c}\partial {\mathcal{F}}^\mathrm{curv}/\partial {X}_{,vu}\\ \begin{array}{c}\partial {\mathcal{F}}^\mathrm{membr-all}/\partial {Y}_{,u}\\ \partial {\mathcal{F}}^\mathrm{membr-all}/\partial {Y}_{,v}\\ \begin{array}{c}\partial {\mathcal{F}}^\mathrm{curv}/\partial {Y}_{,uu}\\ \partial {\mathcal{F}}^\mathrm{curv}/\partial {Y}_{,vv}\\ \begin{array}{c}\partial {\mathcal{F}}^\mathrm{curv}/\partial {Y}_{,uv}\\ \partial {\mathcal{F}}^\mathrm{curv}/\partial {Y}_{,vu}\\ \begin{array}{c}\partial {\mathcal{F}}^\mathrm{membr-all}/\partial {Z}_{,u}\\ \begin{array}{c}\partial {\mathcal{F}}^\mathrm{membr-all }/\partial {Z}_{,v}\\ \partial {\mathcal{F}}^\mathrm{curv}/\partial {Z}_{,uu}\\ \begin{array}{c}\partial {\mathcal{F}}^\mathrm{curv}/\partial {Z}_{,vv}\\ \partial {\mathcal{F}}^\mathrm{curv}/\partial {Z}_{,uv}\\ \begin{array}{c}\partial {\mathcal{F}}^\mathrm{curv}/\partial {Z}_{,vu}\\ \partial {\mathcal{F}}^\mathrm{membr-st-cstr}/\partial \alpha \\ \partial {\mathcal{F}}^\mathrm{lipid-cstr }/\partial {\lambda }^\mathrm{lipid}\end{array}\end{array}\end{array}\end{array}\end{array}\end{array}\end{array}\end{array}\end{array}\end{array}\end{array}\right]}^{T}\left[\begin{array}{c}\delta {X}_{,u}\\ \begin{array}{c}\delta {X}_{,v}\\ \delta {X}_{,uu}\\ \begin{array}{c}\delta {X}_{,vv}\\ \delta {X}_{,uv}\\ \begin{array}{c}\delta {X}_{,vu}\\ \begin{array}{c}\delta {Y}_{,u}\\ \delta {Y}_{,v}\\ \begin{array}{c}\delta {Y}_{,uu}\\ \delta {Y}_{,vv}\\ \begin{array}{c}\delta {Y}_{,uv}\\ \delta {Y}_{,vu}\\ \begin{array}{c}\delta {Z}_{,u}\\ \begin{array}{c}\delta {Z}_{,v}\\ \delta {Z}_{,uu}\\ \begin{array}{c}\delta {Z}_{,vv}\\ \delta {Z}_{,uv}\\ \begin{array}{c}\delta {Z}_{,vu}\\ \delta \alpha \\ {\delta \lambda }^\mathrm{lipid}\end{array}\end{array}\end{array}\end{array}\end{array}\end{array}\end{array}\end{array}\end{array}\end{array}\end{array}\right]dudv-\delta {\lambda }^\mathrm{lipid}{n}_\mathrm{lipid}$$

14 $$+\int \sum_{c=1}^{{n}_\mathrm{crs}}{\left[\begin{array}{c}\partial {\mathcal{F}}_{c}^\mathrm{crs}/\partial X\\ \partial {\mathcal{F}}_{c}^\mathrm{crs-all}/\partial {X}_{,u}\\ \begin{array}{c}\partial {\mathcal{F}}_{c}^\mathrm{crs-all}/\partial {X}_{,v}\\ \partial {\mathcal{F}}_{c}^\mathrm{crs}/\partial Y\\ \begin{array}{c}\partial {\mathcal{F}}_{c}^\mathrm{crs-all }/\partial {Y}_{,u}\\ \partial {\mathcal{F}}_{c}^\mathrm{crs-all}/\partial {Y}_{,v}\\ \begin{array}{c}\partial {\mathcal{F}}_{c}^\mathrm{crs}/\partial Z\\ \partial {\mathcal{F}}_{c}^\mathrm{crs-all}/\partial {Z}_{,u}\\ \begin{array}{c}\begin{array}{c}\partial {\mathcal{F}}_{c}^\mathrm{crs-all}/\partial {Z}_{,v}\\ \begin{array}{c}\partial {\mathcal{F}}_{c}^\mathrm{crs-all}/\partial {K}_{1c}\\ \partial {\mathcal{F}}_{c}^\mathrm{crs-all}/\partial {K}_{2c}\end{array}\end{array}\\ \partial {\mathcal{F}}_{c}^\mathrm{crs-cstr}/\partial {\lambda }_{c}^\mathrm{crs}\end{array}\end{array}\end{array}\end{array}\end{array}\right]}^{T}\left[\begin{array}{c}\delta X\\ \delta {X}_{,u}\\ \begin{array}{c}\delta {X}_{,v}\\ \delta Y\\ \begin{array}{c}\delta {Y}_{,u}\\ \delta {Y}_{,v}\\ \begin{array}{c}\delta Z\\ \delta {Z}_{,u}\\ \begin{array}{c}\begin{array}{c}\delta {Z}_{,v}\\ \begin{array}{c}\delta {K}_{1c}\\ \delta {K}_{2c}\end{array}\end{array}\\ \delta {\lambda }_{c}^\mathrm{crs}\end{array}\end{array}\end{array}\end{array}\end{array}\right]dudv-\sum_{c=1}^{{n}_\mathrm{crs}}{\delta \lambda }_{c}^\mathrm{crs}{\varphi }_{c}$$

where ${\mathcal{F}}^\mathrm{membr-all}={\mathcal{F}}^\mathrm{curv}+{\mathcal{F}}^\mathrm{st}+{\mathcal{F}}^\mathrm{lipid-cstr}$; ${\mathcal{F}}^\mathrm{membr-st-cstr}={\mathcal{F}}^\mathrm{st}+{\mathcal{F}}^\mathrm{lipid-cstr}$; and ${{\mathcal{F}}_{c}^\mathrm{crs-all}=\mathcal{F}}_{c}^\mathrm{crs}+{\mathcal{F}}_{c}^\mathrm{crs-cstr}$. In Eq. 14, $\delta X$, $\delta {X}_{,u}$, $\delta {X}_{,v}$, $\delta {X}_{,uu}$, $\delta {X}_{,vv}$, $\delta {X}_{,uv}$, $\delta {X}_{,vu}$ are the variation of $X$, ${X}_{,u}$, ${X}_{,v}$, ${X}_{,uu}$, ${X}_{,vv}$, ${X}_{,uv}$, ${X}_{,vu}$, respectively. The variation of the functions $Y$, $Z$ and their derivatives; the variation of the lipid number strain $\alpha$; the variation of ${K}_{1c}$ and ${K}_{2c}$; the variation of Lagrange multipliers ${\lambda }^\mathrm{lipid}$ and ${\lambda }_{c}^\mathrm{crs}$ are similarly defined in Eq. 14.

### B-spline based parameterization

Within the framework of parametric finite element methods, the shape of functions $X$, $Y$, $Z$, $\alpha$, ${K}_{1c}$, and ${K}_{2c}$ was approximated by using the B-spline function^11^. First, the function $X$ was parameterized as follows.

15 $$X\left(u,v\right)=\sum_{{i}_\mathrm{x}=1}^{{n}_\mathrm{{\Omega }_{x}}}{N}_{{i}_\mathrm{x}}^\mathrm{{\Omega }_{x}}\left(u,v\right){x}_{{i}_\mathrm{x}}^\mathrm{{\Omega }_{x}}+\sum_{{j}_\mathrm{x}=1}^{{n}_\mathrm{{\Gamma }_{x}}}{N}_{{j}_\mathrm{x}}^\mathrm{{\Gamma }_{x}}\left(u,v\right){x}_{{j}_\mathrm{x}}^\mathrm{{\Gamma }_{x}}$$

Here, $\mathrm{{\Omega }_{x}}$ indicates the domain with the unknown ${x}_{{i}_\mathrm{x}}^\mathrm{{\Omega }_{x}}$. ${\Gamma }_\mathrm{x}$ denotes the domain with the fixed boundary value ${x}_{{j}_\mathrm{x}}^{{\Gamma }_\mathrm{x}}$. ${n}_{{\Omega }_\mathrm{x}}$ and ${n}_{{\Gamma }_\mathrm{x}}$ are the total number of elements for ${x}_{{i}_\mathrm{x}}^\mathrm{{\Omega }_{x}}$ and the total number of elements for ${x}_{{j}_\mathrm{x}}^\mathrm{{\Gamma }_{x}}$, respectively. ${i}_\mathrm{x}$ and ${j}_\mathrm{x}$ are indices for ${x}_{{i}_\mathrm{x}}^\mathrm{{\Omega }_{x}}$ and ${x}_{{j}_\mathrm{x}}^\mathrm{{\Gamma }_{x}}$, respectively. $N\left(u,v\right)$ is the two-dimensional B-spline function. See Fig. S1C for nine basis functions used in this work i.e., ${B}_{1}$ to ${B}_{9}$, to define $N\left(u,v\right)$. Individual equations for ${B}_{1}$ to ${B}_{9}$ with respect to the parent domain $\xi \in \left[-\mathrm{1,1}\right]$ and $\eta \in \left[-\mathrm{1,1}\right]$ are listed in Equations S31‒S39 in SM.

The surface-tangential degree of freedom can cause numerical instabilities for lipid membranes^9,11^. To avoid this zero-energy mode, the weight ${x}_{{i}_\mathrm{x}}^\mathrm{{\Omega }_{x}}$ for the corresponding ${N}_{{i}_\mathrm{x}}^\mathrm{{\Omega }_{x}}\left(u,v\right)$ function was determined by projecting the unknown variable ${d}_{{i}_\mathrm{d}}^\mathrm{{\Omega }_{d}}$ onto the X-axis^9,11^. Here, ${d}_{{i}_\mathrm{d}}^\mathrm{{\Omega }_{d}}$ was defined to the normal direction of a reference surface^9,11^. Since the membrane was stimulated in a stepwise fashion, the reference surface was defined either directly or indirectly (after remeshing) from the solution of the previous displacement step. Therefore, Eq. 15 can be rewritten as follows.

16 $$X\left(u,v\right)=\sum_{{i}_\mathrm{d}=1}^{{n}_\mathrm{{\Omega }_{d}}}{N}_{{i}_\mathrm{d}}^\mathrm{{\Omega }_{d}}\left(u,v\right)\left({x}_{{i}_\mathrm{d}}^\mathrm{ref}+{d}_{{i}_\mathrm{d}}^\mathrm{{\Omega }_{d}}\mathrm{cos}\left({\theta }_{{i}_\mathrm{d}}^\mathrm{X}\right)\right)+\sum_{{j}_\mathrm{d}=1}^{{n}_\mathrm{{\Gamma }_{d}}}{N}_{{j}_\mathrm{d}}^\mathrm{{\Gamma }_{d}}\left(u,v\right){x}_{{j}_\mathrm{d}}^\mathrm{{\Gamma }_{d}}$$

$\mathrm{{\Omega }_{d}}$ is the domain with the unknown ${d}_{{i}_\mathrm{d}}^\mathrm{{\Omega }_{d}}$ and $\mathrm{{\Gamma }_{d}}$ is the domain with the fixed boundary value ${x}_{{j}_\mathrm{d}}^\mathrm{{\Gamma }_{d}}$ (see Fig. S1A). ${n}_\mathrm{{\Omega }_{d}}$ is the number of elements for ${d}_{{i}_\mathrm{d}}^\mathrm{{\Omega }_{d}}$, where ${n}_\mathrm{{\Omega }_{d}}={n}_\mathrm{{\Omega }_{x}}$. Similarly, ${n}_\mathrm{{\Gamma }_{d}}$ is the number of elements for ${x}_{{j}_\mathrm{d}}^\mathrm{{\Gamma }_{d}}$, where ${n}_\mathrm{{\Gamma }_{d}}={n}_\mathrm{{\Gamma }_{x}}$. The value $\mathit{cos}\left({\theta }_{{i}_\mathrm{d}}^\mathrm{X}\right)$ was defined as $\mathrm{cos}\left({\theta }_{{i}_\mathrm{d}}^\mathrm{X}\right)=\frac{{\widehat{n}}_\mathrm{X}}{\sqrt{{{\widehat{n}}_\mathrm{X}}^{2}+{{\widehat{n}}_\mathrm{Y}}^{2}+{{\widehat{n}}_\mathrm{Z}}^{2}}}$ evaluated at the parametric domain center of each element in the reference surface. ${x}_{{i}_\mathrm{d}}^\mathrm{ref}$ is the constant value obtained from the reference surface.

Note that the remeshing process in the presented finite element method was limited for some complex geometries due to failure in finding a proper initial guess for the Newton–Raphson’s method (see “Remeshing process for the finite element model” in SM). While future work will aim to address this limitation, the remeshing step may be omitted in such cases. In the absence of remeshing, the value ${x}_{{i}_\mathrm{d}}^\mathrm{ref}+{d}_{{i}_\mathrm{d}}^\mathrm{{\Omega }_{d}}\mathrm{cos}\left({\theta }_{{i}_\mathrm{d}}^\mathrm{X}\right)$ in the final Newton’s iteration of the current displacement step becomes the new ${x}_{{i}_\mathrm{d}}^\mathrm{ref}$ for the next step (see “Remeshing process for the finite element model” in SM for how ${x}_{{i}_\mathrm{d}}^\mathrm{ref}$ is defined when remeshing is applied).

As similarly done for $X\left(u,v\right)$, the functions $Y$ and $Z$ can be written as follows.

17 $$Y\left(u,v\right)=\sum_{{i}_\mathrm{d}=1}^{{n}_\mathrm{{\Omega }_{d}}}{N}_{{i}_\mathrm{d}}^\mathrm{{\Omega }_{d}}\left(u,v\right)\left({y}_{{i}_\mathrm{d}}^\mathrm{ref}+{d}_{{i}_\mathrm{d}}^\mathrm{{\Omega }_{d}}\mathrm{cos}\left({\theta }_{{i}_\mathrm{d}}^\mathrm{Y}\right)\right)+\sum_{{j}_\mathrm{d}=1}^{{n}_\mathrm{{\Gamma }_{d}}}{N}_{{j}_\mathrm{d}}^\mathrm{{\Gamma }_{d}}\left(u,v\right){y}_{{j}_\mathrm{d}}^\mathrm{{\Gamma }_{d}}$$

and

18 $$Z\left(u,v\right)=\sum_{{i}_\mathrm{d}=1}^{{n}_\mathrm{{\Omega }_{d}}}{N}_{{i}_\mathrm{d}}^\mathrm{{\Omega }_{d}}\left(u,v\right)\left({z}_{{i}_\mathrm{d}}^\mathrm{ref}+{d}_{{i}_\mathrm{d}}^\mathrm{{\Omega }_{d}}\mathrm{cos}\left({\theta }_{{i}_\mathrm{d}}^\mathrm{Z}\right)\right)+\sum_{{j}_\mathrm{d}=1}^{{n}_\mathrm{{\Gamma }_{d}}}{N}_{{j}_\mathrm{d}}^\mathrm{{\Gamma }_{d}}\left(u,v\right){z}_{{j}_\mathrm{d}}^\mathrm{{\Gamma }_{d}}$$

where ${y}_{{i}_\mathrm{d}}^\mathrm{ref}$ and ${z}_{{i}_\mathrm{d}}^\mathrm{ref}$ are constant values defined similarly to ${x}_{{i}_\mathrm{d}}^\mathrm{ref}$. The terms ${y}_{{j}_\mathrm{d}}^\mathrm{{\Gamma }_{d}}$ and ${z}_{{j}_\mathrm{d}}^\mathrm{{\Gamma }_{d}}$ are prescribed boundary constants. The directional cosines are defined as $\mathrm{cos}\left({\theta }_{{i}_\mathrm{d}}^\mathrm{Y}\right)=\frac{{\widehat{n}}_\mathrm{Y}}{\sqrt{{{\widehat{n}}_\mathrm{X}}^{2}+{{\widehat{n}}_\mathrm{Y}}^{2}+{{\widehat{n}}_\mathrm{Z}}^{2}}}$ and $\mathrm{cos}\left({\theta }_{{i}_\mathrm{d}}^\mathrm{Z}\right)=\frac{{\widehat{n}}_\mathrm{Z}}{\sqrt{{{\widehat{n}}_\mathrm{X}}^{2}+{{\widehat{n}}_\mathrm{Y}}^{2}+{{\widehat{n}}_\mathrm{Z}}^{2}}}$ evaluated at the parametric center of each element. In Eqs. 16‒18, ${i}_\mathrm{d}$ is the index for ${d}_{{i}_\mathrm{d}}^\mathrm{{\Omega }_{d}}$, where ${i}_\mathrm{d}={i}_\mathrm{x}$. Similarly, ${j}_\mathrm{d}$ is the index for the constants ${x}_{{j}_\mathrm{d}}^\mathrm{{\Gamma }_{d}}$, ${y}_{{j}_\mathrm{d}}^\mathrm{{\Gamma }_{d}}$, and ${z}_{{j}_\mathrm{d}}^\mathrm{{\Gamma }_{d}}$, where ${j}_\mathrm{d}={j}_\mathrm{x}$. Derivatives for the functions $X$, $Y$, $Z$ with respect to $u$ and $v$ can be obtained by taking derivatives of $N\left(u,v\right)$ from Eqs. 16‒18. Finally, the functions $\alpha$, ${K}_{1c}$, and ${K}_{2c}$ are written as follows.

19 $$\alpha \left(u,v\right)=\sum_{{i}_\mathrm{s}=1}^{{n}_\mathrm{{\Omega }_{s}}}{N}_{{i}_\mathrm{s}}^\mathrm{{\Omega }_{s}}\left(u,v\right){s}_{{i}_\mathrm{s}}^\mathrm{{\Omega }_{s}}+\sum_{{j}_\mathrm{s}=1}^{{n}_\mathrm{{\Gamma }_{s}}}{N}_{{j}_\mathrm{s}}^\mathrm{{\Gamma }_{s}}\left(u,v\right){s}_{{j}_\mathrm{s}}^\mathrm{{\Gamma }_{s}}$$

20 $${K}_{1c}\left(u,v\right)=\sum_{{i}_{\mathrm{k}_{1c}}=1}^{{n}_{\mathrm{\Omega }_{\mathrm{k}_{1c}}}}{N}_{{i}_{\mathrm{k}_{1c}}}^{\mathrm{\Omega }_{\mathrm{k}_{1c}}}\left(u,v\right){k}_{1c{i}_{\mathrm{k}_{1c}}}^{\mathrm{\Omega }_{\mathrm{k}_{1c}}}+\sum_{{j}_{\mathrm{k}_{1c}}=1}^{{n}_{\mathrm{\Gamma }_{\mathrm{k}_{1c}}}}{N}_{{j}_{\mathrm{k}_{1c}}}^{\mathrm{\Gamma }_{\mathrm{k}_{1c}}}\left(u,v\right){k}_{1c{j}_{\mathrm{k}_{1c}}}^{\mathrm{\Gamma }_{\mathrm{k}_{1c}}}$$

and

21 $${K}_{2c}\left(u,v\right)=\sum_{{i}_{\mathrm{k}_{2c}}=1}^{{n}_{\mathrm{\Omega }_{\mathrm{k}_{2c}}}}{N}_{{i}_{\mathrm{k}_{2c}}}^{\mathrm{\Omega }_{\mathrm{k}_{2c}}}\left(u,v\right){k}_{2c{i}_{\mathrm{k}_{2c}}}^{\mathrm{\Omega }_{\mathrm{k}_{2c}}}+\sum_{{j}_{\mathrm{k}_{2c}}=1}^{{n}_{\mathrm{\Gamma }_{\mathrm{k}_{2c}}}}{N}_{{j}_{\mathrm{k}_{2c}}}^{\mathrm{\Gamma }_{\mathrm{k}_{2c}}}\left(u,v\right){k}_{2c{j}_{\mathrm{k}_{2c}}}^{\mathrm{\Gamma }_{\mathrm{k}_{2c}}}$$

Here, $c$ is the index for the group of crosslinkers (see the Results section and Fig. 1E). The domains $\mathrm{{\Omega }_{s}}$, $\mathrm{\Omega }_{\mathrm{k}_{1c}}$, $\mathrm{\Omega }_{\mathrm{k}_{2c}}$ contain unknowns variables ${s}_{{i}_\mathrm{s}}^\mathrm{{\Omega }_{s}}$, ${k}_{1c{i}_{\mathrm{k}_{1c}}}^{\mathrm{\Omega }_{\mathrm{k}_{1c}}}$, ${k}_{2c{i}_{\mathrm{k}_{2c}}}^{\mathrm{\Omega }_{\mathrm{k}_{2c}}}$, respectively. On the other hand, the domains $\mathrm{{\Gamma }_{s}}$, $\mathrm{\Gamma }_{\mathrm{k}_{1c}}$, $\mathrm{\Gamma }_{\mathrm{k}_{2c}}$ contain fixed boundary values ${s}_{{j}_\mathrm{s}}^\mathrm{{\Gamma }_{s}}$, ${k}_{1c{j}_{\mathrm{k}_{1c}}}^{\mathrm{\Gamma }_{\mathrm{k}_{1c}}}$, ${k}_{2c{j}_{\mathrm{k}_{2c}}}^{\mathrm{\Gamma }_{\mathrm{k}_{2c}}}$, respectively. ${i}_\mathrm{s}$, ${i}_{\mathrm{k}_{1c}}$, ${i}_{\mathrm{k}_{2c}}$ are indices for the unknowns, while ${j}_\mathrm{s}$, ${j}_{\mathrm{k}_{1c}}$, ${j}_{\mathrm{k}_{2c}}$ are indices for the boundary values. ${n}_\mathrm{{\Omega }_{s}}$, ${n}_{\mathrm{\Omega }_{\mathrm{k}_{1c}}}$, ${n}_{\mathrm{\Omega }_{\mathrm{k}_{2c}}}$ are the number of elements for ${s}_{{i}_\mathrm{s}}^\mathrm{{\Omega }_{s}}$, ${k}_{1c{i}_{\mathrm{k}_{1c}}}^{\mathrm{\Omega }_{\mathrm{k}_{1c}}}$, ${k}_{2c{i}_{\mathrm{k}_{2c}}}^{\mathrm{\Omega }_{\mathrm{k}_{2c}}}$, respectively. ${n}_\mathrm{{\Gamma }_{s}}$, ${n}_{\mathrm{\Gamma }_{\mathrm{k}_{1c}}}$, ${n}_{\mathrm{\Gamma }_{\mathrm{k}_{2c}}}$ are the number of elements for ${s}_{{j}_\mathrm{s}}^\mathrm{{\Gamma }_{s}}$, ${k}_{1c{j}_{\mathrm{k}_{1c}}}^{\mathrm{\Gamma }_{\mathrm{k}_{1c}}}$, ${k}_{2c{j}_{\mathrm{k}_{2c}}}^{\mathrm{\Gamma }_{\mathrm{k}_{2c}}}$, respectively. How the degrees of freedom are defined on the parametric domains can be seen in Figs. S2C, S10, S13‒S18.

The variations of the functions $X$, $Y$, $Z$, $\alpha$, ${K}_{1c}$, ${K}_{2c}$ i.e., $\delta X$, $\delta Y$, $\delta Z$, $\delta \alpha$, $\delta {K}_{1c}$, $\delta {K}_{2c}$, are similarly defined as written in Eqs. 22‒27.

22 $$\delta X\left(u,v\right)=\sum_{{i}_\mathrm{d}=1}^{{n}_\mathrm{{\Omega }_{d}}}{N}_{{i}_\mathrm{d}}^\mathrm{{\Omega }_{d}}\left(u,v\right){\overline{d} }_{{i}_\mathrm{d}}\mathrm{cos}\left({\theta }_{{i}_\mathrm{d}}^\mathrm{X}\right)$$

23 $$\delta Y\left(u,v\right)=\sum_{{i}_\mathrm{d}=1}^{{n}_\mathrm{{\Omega }_{d}}}{N}_{{i}_\mathrm{d}}^\mathrm{{\Omega }_{d}}\left(u,v\right){\overline{d} }_{{i}_\mathrm{d}}\mathrm{cos}\left({\theta }_{{i}_\mathrm{d}}^\mathrm{Y}\right)$$

24 $$\delta Z\left(u,v\right)=\sum_{{i}_\mathrm{d}=1}^{{n}_\mathrm{{\Omega }_{d}}}{N}_{{i}_\mathrm{d}}^\mathrm{{\Omega }_{d}}\left(u,v\right){\overline{d} }_{{i}_\mathrm{d}}\mathrm{cos}\left({\theta }_{{i}_\mathrm{d}}^\mathrm{Z}\right)$$

25 $$\delta \alpha \left(u,v\right)=\sum_{{i}_\mathrm{s}=1}^{{n}_\mathrm{{\Omega }_{s}}}{N}_{{i}_\mathrm{s}}^\mathrm{{\Omega }_{s}}\left(u,v\right){\overline{s} }_{{i}_\mathrm{s}}$$

26 $${\delta K}_{1c}\left(u,v\right)=\sum_{{i}_{\mathrm{k}_{1c}}=1}^{{n}_{\mathrm{\Omega }_{\mathrm{k}_{1c}}}}{N}_{{i}_{\mathrm{k}_{1c}}}^{\mathrm{\Omega }_{\mathrm{k}_{1c}}}\left(u,v\right){\overline{k} }_{1c{i}_{\mathrm{k}_{1c}}}$$

and

27 $${\delta K}_{2c}\left(u,v\right)=\sum_{{i}_{\mathrm{k}_{2c}}=1}^{{n}_{\mathrm{\Omega }_{\mathrm{k}_{2c}}}}{N}_{{i}_{\mathrm{k}_{2c}}}^{\mathrm{\Omega }_{\mathrm{k}_{2c}}}\left(u,v\right){\overline{k} }_{2c{i}_{\mathrm{k}_{2c}}}$$

Here, ${\overline{d} }_{{i}_\mathrm{d}}$, ${\overline{s} }_{{i}_\mathrm{s}}$, ${\overline{k} }_{1c{i}_{\mathrm{k}_{1c}}}$, and ${\overline{k} }_{2c{i}_{\mathrm{k}_{2c}}}$ are unknown values. Derivatives of $\delta X$, $\delta Y$, and $\delta Z$ can be defined by taking derivatives of $N\left(u,v\right)$ with respect to $u$ and $v$ from Eqs. 22‒24.

### The Newton–Raphson method for nonlinear simultaneous equations

The condition to find finite element solutions is that the variation of the functional is equal to zero i.e., $\delta {\Pi }^\mathrm{tot}=0$. To this end, the parameterized functions $X$, $Y$, $Z$ (Eqs. 16‒18); the lipid number strain $\alpha$ (Eq. 19); ${K}_{1c}$ and ${K}_{2c}$ (Eqs. 20, 21); and their variations (Eqs. 22‒27) were substituted into Eq. 14. The condition $\delta {\Pi }^\mathrm{tot}=0$ and the arbitrariness of ${\overline{d} }_{{i}_\mathrm{d}}$, ${\overline{s} }_{{i}_\mathrm{s}}$, ${\overline{k} }_{1c{i}_{\mathrm{k}_{1c}}}$, ${\overline{k} }_{2c{i}_{\mathrm{k}_{2c}}}$, ${\delta \lambda }^\mathrm{lipid}$, and $\delta {\lambda }_{c}^\mathrm{crs}$ resulted in coupled ${n}_\mathrm{equ}$ nonlinear simultaneous equations where ${n}_\mathrm{equ}={n}_\mathrm{{\Omega }_{d}}+{n}_\mathrm{{\Omega }_{s}}+\sum_{\overline{c }=1}^{{n}_\mathrm{crs}}{n}_{\mathrm{\Omega }_{\mathrm{k}_{1\overline{c}}} }+\sum_{\overline{c }=1}^{{n}_\mathrm{crs}}{n}_{\mathrm{\Omega }_{\mathrm{k}_{2\overline{c}}} }+1+{n}_\mathrm{crs}$.

To use the Newton–Raphson method, the residual vector $\mathcal{G}$ is defined as follows.

28 $$\mathcal{G}=\left({G}_{\left(a1\right)}\right)$$

In addition, the following definitions are further made. ${D}_{1}=X$, ${D}_{2}={X}_{,u}$, ${D}_{3}={X}_{,v}$, ${D}_{4}={X}_{,uu}$, ${D}_{5}={X}_{,vv}$, ${D}_{6}={X}_{,uv}$, ${D}_{7}={X}_{,vu}$, ${D}_{8}=Y$, ${D}_{9}={Y}_{,u}$, ${D}_{10}={Y}_{,v}$, ${D}_{11}={Y}_{,uu}$, ${D}_{12}={Y}_{,vv}$, ${D}_{13}={Y}_{,uv}$, ${D}_{14}={Y}_{,vu}$, ${D}_{15}=Z$, ${D}_{16}={Z}_{,u}$, ${D}_{17}={Z}_{,v}$, ${D}_{18}={Z}_{,uu}$, ${D}_{19}={Z}_{,vv}$, ${D}_{20}={Z}_{,uv}$, ${D}_{21}={Z}_{,vu}$, ${\overline{N} }_{1}={N}_{a}^\mathrm{{\Omega }_{d}}\mathrm{cos}\left({\theta }_{a}^\mathrm{X}\right)$, ${\overline{N} }_{2}={N}_{a,u}^\mathrm{{\Omega }_{d}}\mathrm{cos}\left({\theta }_{a}^\mathrm{X}\right)$, ${\overline{N} }_{3}={N}_{a,v}^\mathrm{{\Omega }_{d}}\mathrm{cos}\left({\theta }_{a}^\mathrm{X}\right)$, ${\overline{N} }_{4}={N}_{a,uu}^\mathrm{{\Omega }_{d}}\mathrm{cos}\left({\theta }_{a}^\mathrm{X}\right)$, ${\overline{N} }_{5}={N}_{a,vv}^\mathrm{{\Omega }_{d}}\mathrm{cos}\left({\theta }_{a}^\mathrm{X}\right)$, ${\overline{N} }_{6}={N}_{a,uv}^\mathrm{{\Omega }_{d}}\mathrm{cos}\left({\theta }_{a}^\mathrm{X}\right)$, ${\overline{N} }_{7}={N}_{a,vu}^\mathrm{{\Omega }_{d}}\mathrm{cos}\left({\theta }_{a}^\mathrm{X}\right)$, ${\overline{N} }_{8}={N}_{a}^\mathrm{{\Omega }_{d}}\mathrm{cos}\left({\theta }_{a}^\mathrm{Y}\right)$, ${\overline{N} }_{9}={N}_{a,u}^\mathrm{{\Omega }_{d}}\mathrm{cos}\left({\theta }_{a}^\mathrm{Y}\right)$, ${\overline{N} }_{10}={N}_{a,v}^\mathrm{{\Omega }_{d}}\mathrm{cos}\left({\theta }_{a}^\mathrm{Y}\right)$, ${\overline{N} }_{11}={N}_{a,uu}^\mathrm{{\Omega }_{d}}\mathrm{cos}\left({\theta }_{a}^\mathrm{Y}\right)$, ${\overline{N} }_{12}={N}_{a,vv}^\mathrm{{\Omega }_{d}}\mathrm{cos}\left({\theta }_{a}^\mathrm{Y}\right)$, ${\overline{N} }_{13}={N}_{a,uv}^\mathrm{{\Omega }_{d}}\mathrm{cos}\left({\theta }_{a}^\mathrm{Y}\right)$, ${\overline{N} }_{14}={N}_{a,vu}^\mathrm{{\Omega }_{d}}\mathrm{cos}\left({\theta }_{a}^\mathrm{Y}\right)$, ${\overline{N} }_{15}={N}_{a}^\mathrm{{\Omega }_{d}}\mathrm{cos}\left({\theta }_{a}^\mathrm{Z}\right)$, ${\overline{N} }_{16}={N}_{a,u}^\mathrm{{\Omega }_{d}}\mathrm{cos}\left({\theta }_{a}^\mathrm{Z}\right)$, ${\overline{N} }_{17}={N}_{a,v}^\mathrm{{\Omega }_{d}}\mathrm{cos}\left({\theta }_{a}^\mathrm{Z}\right)$, ${\overline{N} }_{18}={N}_{a,uu}^\mathrm{{\Omega }_{d}}\mathrm{cos}\left({\theta }_{a}^\mathrm{Z}\right)$, ${\overline{N} }_{19}={N}_{a,vv}^\mathrm{{\Omega }_{d}}\mathrm{cos}\left({\theta }_{a}^\mathrm{Z}\right)$, ${\overline{N} }_{20}={N}_{a,uv}^\mathrm{{\Omega }_{d}}\mathrm{cos}\left({\theta }_{a}^\mathrm{Z}\right)$, ${\overline{N} }_{21}={N}_{a,vu}^\mathrm{{\Omega }_{d}}\mathrm{cos}\left({\theta }_{a}^\mathrm{Z}\right)$. Elements of the vector $\mathcal{G}$ for $a\le {n}_\mathrm{{\Omega }_{d}}$ can then be defined as follows.

29 $${G}_{\left(a1\right)}=\int {\left({{\mathcal{F}}_{,{D}_{m}}^\mathrm{membr-all}}_{\left(m1\right)}\right)}^{T}\left({{\overline{N} }_{m}}_{\left(m1\right)}\right)+\int \sum_{c=1}^{{n}_\mathrm{crs}}{\left({{\mathcal{F}}_{c,{D}_{m}}^\mathrm{crs-all }}_{\left(m1\right)}\right)}^{T}\left({{\overline{N} }_{m}}_{\left(m1\right)}\right)dudv$$

where $1\le \mathrm{m}\le 21$. See Equation S40 in SM for the expansion of Eq. 29.

Elements of $\mathcal{G}$ for ${n}_\mathrm{{\Omega }_{d}}<a\le {n}_\mathrm{{\Omega }_{d}}+{n}_\mathrm{{\Omega }_{s}}$ is

30 $${G}_{\left(a1\right)}=\int {\mathcal{F}}_{,\alpha }^\mathrm{membr-st-cstr }{N}_{a-{n}_\mathrm{{\Omega }_{d}}}^\mathrm{{\Omega }_{s}}dudv$$

Elements of $\mathcal{G}$ for ${n}_\mathrm{{\Omega }_{d}}+{n}_\mathrm{{\Omega }_{s}}+\sum_{\overline{c }=1}^{c-1}{n}_{\mathrm{\Omega }_{\mathrm{k}_{1\overline{c}}} }<a\le {n}_\mathrm{{\Omega }_{d}}+{n}_\mathrm{{\Omega }_{s}}+\sum_{\overline{c }=1}^{c}{n}_{\mathrm{\Omega }_{\mathrm{k}_{1\overline{c}}} }$ is

31 $${G}_{\left(a1\right)}=\int {\mathcal{F}}_{c,{K}_{1c}}^\mathrm{crs-all}{N}_{a-{n}_\mathrm{{\Omega }_{d}}-{n}_\mathrm{{\Omega }_{s}}-\sum_{\overline{c }=1}^{c-1}{n}_{\mathrm{\Omega }_{\mathrm{k}_{1\overline{c}}}} }^{\mathrm{\Omega }_{\mathrm{k}_{1c}}}dudv$$

Elements of $\mathcal{G}$ for ${n}_\mathrm{{\Omega }_{d}}+{n}_\mathrm{{\Omega }_{s}}+\sum_{\overline{c }=1}^{{n}_\mathrm{crs}}{n}_{\mathrm{\Omega }_{\mathrm{k}_{1\overline{c}}} }+\sum_{\overline{c }=1}^{c-1}{n}_{\mathrm{\Omega }_{\mathrm{k}_{2\overline{c}}} }<a\le {n}_\mathrm{{\Omega }_{d}}+{n}_\mathrm{{\Omega }_{s}}+\sum_{\overline{c }=1}^{{n}_\mathrm{crs}}{n}_{\mathrm{\Omega }_{\mathrm{k}_{1\overline{c}}} }+\sum_{\overline{c }=1}^{c}{n}_{\mathrm{\Omega }_{\mathrm{k}_{2\overline{c}}} }$ is

32 $${G}_{\left(a1\right)}=\int {\mathcal{F}}_{c,{K}_{2c}}^\mathrm{crs-all}{N}_{a-{n}_\mathrm{{\Omega }_{d}}-{n}_\mathrm{{\Omega }_{s}}-\sum_{\overline{c }=1}^{{n}_\mathrm{crs}}{n}_{\mathrm{\Omega }_{\mathrm{k}_{1\overline{c}}} }-\sum_{\overline{c }=1}^{c-1}{n}_{\mathrm{\Omega }_{\mathrm{k}_{2\overline{c}}}} }^{\mathrm{\Omega }_{\mathrm{k}_{2c}}}dudv$$

Elements of $\mathcal{G}$ for $a={n}_\mathrm{{\Omega }_{d}}+{n}_\mathrm{{\Omega }_{s}}+\sum_{\overline{c }=1}^{{n}_\mathrm{crs}}{n}_{\mathrm{\Omega }_{\mathrm{k}_{1\overline{c}}} }+\sum_{\overline{c }=1}^{{n}_\mathrm{crs}}{n}_{\mathrm{\Omega }_{\mathrm{k}_{2\overline{c}}} }+1$ is

33 $${G}_{\left(a1\right)}=\int {\mathcal{F}}_{,{\lambda }^\mathrm{lipid}}^\mathrm{lipid-cstr}dudv-{n}_\mathrm{lipid}$$

Elements of $\mathcal{G}$ for $a={n}_\mathrm{{\Omega }_{d}}+{n}_\mathrm{{\Omega }_{s}}+\sum_{\overline{c }=1}^{{n}_\mathrm{crs}}{n}_{\mathrm{\Omega }_{\mathrm{k}_{1\overline{c}}} }+\sum_{\overline{c }=1}^{{n}_\mathrm{crs}}{n}_{\mathrm{\Omega }_{\mathrm{k}_{2\overline{c}}} }+1+c$ is

34 $${G}_{\left(a1\right)}=\int {\mathcal{F}}_{c,{\lambda }_{c}^\mathrm{crs}}^\mathrm{crs-cstr}dudv-{\varphi }_{c}$$

The ${n}_\mathrm{equ}$-by-${n}_\mathrm{equ}$ Jacobian matrix $\mathcal{J}$, that serves as the tangential operator in using the Newton–Raphson method, is defined as follows to solve the nonlinear equations iteratively.

35 $$\mathcal{J}=\left({J}_{\left(ab\right)}\right)=\left({\frac{{\partial G}_{a}}{\partial {d}_{b}^\mathrm{dof}}}_{\left(ab\right)}\right)$$

Here, ${J}_{\left(ab\right)}$ indicates the element in the ${a}$-th row and ${b}$-th column of $\mathcal{J}$, that can be either calculated from expressions in Eqs. 36‒55 or defined to be zero. ${G}_{a}$ is equal to ${G}_{\left(a1\right)}$ in Eq. 28. ${d}_{b}^\mathrm{dof}$ is the unknown for degrees of freedom (DOFs). $1\le m\le 21$ and/or $1\le n\le 21$ hold for Eqs. 36‒42, 45, 48, 51, and 53.

For $$a\le {n}_\mathrm{{\Omega }_{d}}$$ and $$b\le {n}_\mathrm{{\Omega }_{d}}$$,

$${\frac{{\partial G}_{a}}{\partial {d}_{b}^\mathrm{dof}}}_{\left(ab\right)}=\int {\left[\left({{\mathcal{F}}_{,{D}_{m}{D}_{n}}^\mathrm{membr-all }}_{\left(mn\right)}\right)\left({{D}_{n,{d}_{b}^\mathrm{{\Omega }_{d}}}}_{\left(n1\right)}\right)\right]}^{T}\left({{\overline{N} }_{m}}_{\left(m1\right)}\right)dudv$$

36 $$+\int \begin{array}{c}\sum_{c=1}^{{n}_\mathrm{crs}}{\left[\left({{\mathcal{F}}_{c,{D}_{m}{D}_{n}}^\mathrm{crs-all }}_{\left(mn\right)}\right)\left({{D}_{n,{d}_{b}^\mathrm{{\Omega }_{d}}}}_{\left(n1\right)}\right)\right]}^{T}\left({{\overline{N} }_{m}}_{\left(m1\right)}\right)dudv\end{array}$$

See Equation S41 for the expansion of Eq. 36.

For $$a\le {n}_\mathrm{{\Omega }_{d}}$$ and $${n}_\mathrm{{\Omega }_{d}}<b\le {n}_\mathrm{{\Omega }_{d}}+{n}_\mathrm{{\Omega }_{s}}$$,

37 $${\frac{{\partial G}_{a}}{\partial {d}_{b}^\mathrm{dof}}}_{\left(ab\right)}=\int \begin{array}{c}{\left[\left({{\mathcal{F}}_{,{D}_{m}\alpha }^\mathrm{membr-st-cstr }}_{\left(m1\right)}\right){\alpha }_{,{s}_{b-{n}_\mathrm{{\Omega }_{d}}}^\mathrm{{\Omega }_{s}}}\right]}^{T}\left({{\overline{N} }_{m}}_{\left(m1\right)}\right)dudv\end{array}$$

See Equation S42 for the expansion of Eq. 37.

For $$a\le {n}_\mathrm{{\Omega }_{d}}$$ and $${n}_\mathrm{{\Omega }_{d}}+{n}_\mathrm{{\Omega }_{s}}+\sum_{\overline{c }=1}^{c-1}{n}_{\mathrm{\Omega }_{\mathrm{k}_{1\overline{c}}} }<b\le {n}_\mathrm{{\Omega }_{d}}+{n}_\mathrm{{\Omega }_{s}}+\sum_{\overline{c }=1}^{c}{n}_{\mathrm{\Omega }_{\mathrm{k}_{1\overline{c}}} }$$,

38 $${\frac{{\partial G}_{a}}{\partial {d}_{b}^\mathrm{dof}}}_{\left(ab\right)}=\int \begin{array}{c}{\left[\left({{\mathcal{F}}_{c,{D}_{m}{K}_{1c}}^\mathrm{crs-all }}_{\left(m1\right)}\right) {K}_{1c,{k}_{1c\left(b-{n}_\mathrm{{\Omega }_{d}}-{n}_\mathrm{{\Omega }_{s}}-\sum_{\overline{c }=1}^{c-1}{n}_{\mathrm{\Omega }_{\mathrm{k}_{1\overline{c}}} }\right)}^{\mathrm{\Omega }_{\mathrm{k}_{1c}}}}\right]}^{T}\left({{\overline{N} }_{m}}_{\left(m1\right)}\right)dudv\end{array}$$

See Equation S43 for the expansion of Eq. 38.

For $$a\le {n}_\mathrm{{\Omega }_{d}}$$ and $${n}_\mathrm{{\Omega }_{d}}+{n}_\mathrm{{\Omega }_{s}}+\sum_{\overline{c }=1}^{{n}_\mathrm{crs}}{n}_{\mathrm{\Omega }_{\mathrm{k}_{1\overline{c}}} }+\sum_{\overline{c }=1}^{c-1}{n}_{\mathrm{\Omega }_{\mathrm{k}_{2\overline{c}}} }<b\le {n}_\mathrm{{\Omega }_{d}}+{n}_\mathrm{{\Omega }_{s}}+\sum_{\overline{c }=1}^{{n}_\mathrm{crs}}{n}_{\mathrm{\Omega }_{\mathrm{k}_{1\overline{c}}} }+\sum_{\overline{c }=1}^{c}{n}_{\mathrm{\Omega }_{\mathrm{k}_{2\overline{c}}} }$$,

39 $${\frac{{\partial G}_{a}}{\partial {d}_{b}^\mathrm{dof}}}_{\left(ab\right)}=\int \begin{array}{c}{\left[\left({{\mathcal{F}}_{c,{D}_{m}{K}_{2c}}^\mathrm{crs-all}}_{\left(m1\right)}\right) {K}_{2c,{k}_{2c\left(b-{n}_\mathrm{{\Omega }_{d}}-{n}_\mathrm{{\Omega }_{s}}-\sum_{\overline{c }=1}^{{n}_\mathrm{crs}}{n}_{\mathrm{\Omega }_{\mathrm{k}_{1\overline{c}}} }-\sum_{\overline{c }=1}^{c-1}{n}_{\mathrm{\Omega }_{\mathrm{k}_{2\overline{c}}} }\right)}^{\mathrm{\Omega }_{\mathrm{k}_{2c}}}}\right]}^{T}\left({{\overline{N} }_{m}}_{\left(m1\right)}\right)dudv\end{array}$$

See Equation S44 for the expansion of Eq. 39.

For $$a\le {n}_\mathrm{{\Omega }_{d}}$$ and $$b={n}_\mathrm{{\Omega }_{d}}+{n}_\mathrm{{\Omega }_{s}}+\sum_{\overline{c }=1}^{{n}_\mathrm{crs}}{n}_{\mathrm{\Omega }_{\mathrm{k}_{1\overline{c}}} }+\sum_{\overline{c }=1}^{{n}_\mathrm{crs}}{n}_{\mathrm{\Omega }_{\mathrm{k}_{2\overline{c}}} }+1$$,

40 $${\frac{{\partial G}_{a}}{\partial {d}_{b}^\mathrm{dof}}}_{\left(ab\right)}=\int {\left({{\mathcal{F}}_{,{D}_{m}{\lambda }^\mathrm{lipid}}^\mathrm{lipid-cstr}}_{\left(m1\right)}\right)}^{T}\left({{\overline{N} }_{m}}_{\left(m1\right)}\right)\begin{array}{c}dudv\end{array}$$

See Equation S45 for the expansion of Eq. 40.

For $$a\le {n}_\mathrm{{\Omega }_{d}}$$ and $$b={n}_\mathrm{{\Omega }_{d}}+{n}_\mathrm{{\Omega }_{s}}+\sum_{\overline{c }=1}^{{n}_\mathrm{crs}}{n}_{\mathrm{\Omega }_{\mathrm{k}_{1\overline{c}}} }+\sum_{\overline{c }=1}^{{n}_\mathrm{crs}}{n}_{\mathrm{\Omega }_{\mathrm{k}_{2\overline{c}}} }+1+c$$,

41 $${\frac{{\partial G}_{a}}{\partial {d}_{b}^\mathrm{dof}}}_{\left(ab\right)}=\int \begin{array}{c}{\left({{\mathcal{F}}_{c,{D}_{m}{\lambda }_{c}^\mathrm{crs}}^\mathrm{crs-cstr}}_{\left(m1\right)}\right)}^{T}\left({{\overline{N} }_{m}}_{\left(m1\right)}\right)dudv\end{array}$$

See Equation S46 for the expansion of Eq. 41.

For $${n}_\mathrm{{\Omega }_{d}}<a\le {n}_\mathrm{{\Omega }_{d}}+{n}_\mathrm{{\Omega }_{s}}$$ and $$b\le {n}_\mathrm{{\Omega }_{d}}$$

42 $${\frac{{\partial G}_{a}}{\partial {d}_{b}^\mathrm{dof}}}_{\left(ab\right)}=\int \begin{array}{c}\left[\left({{\mathcal{F}}_{,\alpha {D}_{n}}^\mathrm{membr-st-cstr}}_{\left(1n\right)}\right)\left({{D}_{n,{d}_{b}^\mathrm{{\Omega }_{d}}}}_{\left(n1\right)}\right)\right] {N}_{a-{n}_\mathrm{{\Omega }_{d}}}^\mathrm{{\Omega }_{s}}dudv\end{array}$$

See Equation S47 for the expansion of Eq. 42.

For $${n}_\mathrm{{\Omega }_{d}}<a\le {n}_\mathrm{{\Omega }_{d}}+{n}_\mathrm{{\Omega }_{s}}$$ and $${n}_\mathrm{{\Omega }_{d}}<b\le {n}_\mathrm{{\Omega }_{d}}+{n}_\mathrm{{\Omega }_{s}}$$,

43 $${\frac{{\partial G}_{a}}{\partial {d}_{b}^\mathrm{dof}}}_{\left(ab\right)}=\int \begin{array}{c}{\mathcal{F}}_{,\alpha \alpha }^\mathrm{membr-st-cstr} {\alpha }_{,{s}_{b-{n}_\mathrm{{\Omega }_{d}}}^\mathrm{{\Omega }_{s}}} {N}_{a-{n}_\mathrm{{\Omega }_{d}}}^\mathrm{{\Omega }_{s}}dudv\end{array}$$

For $${n}_\mathrm{{\Omega }_{d}}<a\le {n}_\mathrm{{\Omega }_{d}}+{n}_\mathrm{{\Omega }_{s}}$$ and $$b={n}_\mathrm{{\Omega }_{d}}+{n}_\mathrm{{\Omega }_{s}}+\sum_{\overline{c }=1}^{{n}_\mathrm{crs}}{n}_{\mathrm{\Omega }_{\mathrm{k}_{1\overline{c}}} }+\sum_{\overline{c }=1}^{{n}_\mathrm{crs}}{n}_{\mathrm{\Omega }_{\mathrm{k}_{2\overline{c}}} }+1$$,

44 $${\frac{{\partial G}_{a}}{\partial {d}_{b}^\mathrm{dof}}}_{\left(ab\right)}=\int \begin{array}{c}{\mathcal{F}}_{,\alpha {\lambda }^\mathrm{lipid}}^\mathrm{lipid-cstr} {N}_{a-{n}_\mathrm{{\Omega }_{d}}}^\mathrm{{\Omega }_{s}}dudv\end{array}$$

For $${n}_\mathrm{{\Omega }_{d}}+{n}_\mathrm{{\Omega }_{s}}+\sum_{\overline{c }=1}^{c-1}{n}_{\mathrm{\Omega }_{\mathrm{k}_{1\overline{c}}} }<a\le {n}_\mathrm{{\Omega }_{d}}+{n}_\mathrm{{\Omega }_{s}}+\sum_{\overline{c }=1}^{c}{n}_{\mathrm{\Omega }_{\mathrm{k}_{1\overline{c}}} }$$ and $$b\le {n}_\mathrm{{\Omega }_{d}}$$,

45 $${\frac{{\partial G}_{a}}{\partial {d}_{b}^\mathrm{dof}}}_{\left(ab\right)}=\int \begin{array}{c}\left[\left({{\mathcal{F}}_{c,{K}_{1c}{D}_{n}}^\mathrm{crs-all}}_{\left(1n\right)}\right)\left({{D}_{n,{d}_{b}^\mathrm{{\Omega }_{d}}}}_{\left(n1\right)}\right)\right] {N}_{a-{n}_\mathrm{{\Omega }_{d}}-{n}_\mathrm{{\Omega }_{s}}-\sum_{\overline{c }=1}^{c-1}{n}_{\mathrm{\Omega }_{\mathrm{k}_{1\overline{c}}}} }^{\mathrm{\Omega }_{\mathrm{k}_{1c}}}dudv\end{array}$$

See Equation S48 for the expansion of Eq. 45.

For $${n}_\mathrm{{\Omega }_{d}}+{n}_\mathrm{{\Omega }_{s}}+\sum_{\overline{c }=1}^{c-1}{n}_{\mathrm{\Omega }_{\mathrm{k}_{1\overline{c}}} }<a\le {n}_\mathrm{{\Omega }_{d}}+{n}_\mathrm{{\Omega }_{s}}+\sum_{\overline{c }=1}^{c}{n}_{\mathrm{\Omega }_{\mathrm{k}_{1\overline{c}}} }$$ and $${n}_\mathrm{{\Omega }_{d}}+{n}_\mathrm{{\Omega }_{s}}+\sum_{\overline{c }=1}^{c-1}{n}_{\mathrm{\Omega }_{\mathrm{k}_{1\overline{c}}} }<b\le {n}_\mathrm{{\Omega }_{d}}+{n}_\mathrm{{\Omega }_{s}}+\sum_{\overline{c }=1}^{c}{n}_{\mathrm{\Omega }_{\mathrm{k}_{1\overline{c}}} }$$,

46 $${\frac{{\partial G}_{a}}{\partial {d}_{b}^\mathrm{dof}}}_{\left(ab\right)}=\int \begin{array}{c}\left({\mathcal{F}}_{c,{K}_{1c}{K}_{1c}}^\mathrm{crs} {K}_{1c,{k}_{1c\left(b-{n}_\mathrm{{\Omega }_{d}}-{n}_\mathrm{{\Omega }_{s}}-\sum_{\overline{c }=1}^{c-1}{n}_{\mathrm{\Omega }_{\mathrm{k}_{1\overline{c}}} }\right)}^{\mathrm{\Omega }_{\mathrm{k}_{1c}}}} {N}_{a-{n}_\mathrm{{\Omega }_{d}}-{n}_\mathrm{{\Omega }_{s}}-\sum_{\overline{c }=1}^{c-1}{n}_{\mathrm{\Omega }_{\mathrm{k}_{1\overline{c}}}} }^{\mathrm{\Omega }_{\mathrm{k}_{1c}}}\right)dudv\end{array}$$

For $${n}_\mathrm{{\Omega }_{d}}+{n}_\mathrm{{\Omega }_{s}}+\sum_{\overline{c }=1}^{c-1}{n}_{\mathrm{\Omega }_{\mathrm{k}_{1\overline{c}}} }<a\le {n}_\mathrm{{\Omega }_{d}}+{n}_\mathrm{{\Omega }_{s}}+\sum_{\overline{c }=1}^{c}{n}_{\mathrm{\Omega }_{\mathrm{k}_{1\overline{c}}} }$$ and $$b={n}_\mathrm{{\Omega }_{d}}+{n}_\mathrm{{\Omega }_{s}}+\sum_{\overline{c }=1}^{{n}_\mathrm{crs}}{n}_{\mathrm{\Omega }_{\mathrm{k}_{1\overline{c}}} }+\sum_{\overline{c }=1}^{{n}_\mathrm{crs}}{n}_{\mathrm{\Omega }_{\mathrm{k}_{2\overline{c}}} }+1+c$$,

47 $${\frac{{\partial G}_{a}}{\partial {d}_{b}^\mathrm{dof}}}_{\left(ab\right)}=\int \begin{array}{c}\left({\mathcal{F}}_{c,{K}_{1c}{\lambda }_{b-{n}_\mathrm{{\Omega }_{d}}-{n}_\mathrm{{\Omega }_{s}}-\sum_{\overline{c }=1}^{{n}_\mathrm{crs}}{n}_{\mathrm{\Omega }_{\mathrm{k}_{1\overline{c}}} }-\sum_{\overline{c }=1}^{{n}_\mathrm{crs}}{n}_{\mathrm{\Omega }_{\mathrm{k}_{2\overline{c}}} }-1}^\mathrm{crs}}^\mathrm{crs-cstr} {N}_{a-{n}_\mathrm{{\Omega }_{d}}-{n}_\mathrm{{\Omega }_{s}}-\sum_{\overline{c }=1}^{c-1}{n}_{\mathrm{\Omega }_{\mathrm{k}_{1\overline{c}}}} }^{\mathrm{\Omega }_{\mathrm{k}_{1c}}}\right)dudv\end{array}$$

For $${n}_\mathrm{{\Omega }_{d}}+{n}_\mathrm{{\Omega }_{s}}+\sum_{\overline{c }=1}^{{n}_\mathrm{crs}}{n}_{\mathrm{\Omega }_{\mathrm{k}_{1\overline{c}}} }+\sum_{\overline{c }=1}^{c-1}{n}_{\mathrm{\Omega }_{\mathrm{k}_{2\overline{c}}} }<a\le {n}_\mathrm{{\Omega }_{d}}+{n}_\mathrm{{\Omega }_{s}}+\sum_{\overline{c }=1}^{{n}_\mathrm{crs}}{n}_{\mathrm{\Omega }_{\mathrm{k}_{1\overline{c}}} }+\sum_{\overline{c }=1}^{c}{n}_{\mathrm{\Omega }_{\mathrm{k}_{2\overline{c}}} }$$ and $$b\le {n}_\mathrm{{\Omega }_{d}}$$,

48 $${\frac{{\partial G}_{a}}{\partial {d}_{b}^\mathrm{dof}}}_{\left(ab\right)}=\int \left[\left({{\mathcal{F}}_{c,{K}_{2c}{D}_{n}}^\mathrm{crs-all}}_{\left(1n\right)}\right)\left({{D}_{n,{d}_{b}^\mathrm{{\Omega }_{d}}}}_{\left(n1\right)}\right)\right]{N}_{a-{n}_\mathrm{{\Omega }_{d}}-{n}_\mathrm{{\Omega }_{s}}-\sum_{\overline{c }=1}^{{n}_\mathrm{crs}}{n}_{\mathrm{\Omega }_{\mathrm{k}_{1\overline{c}}} }-\sum_{\overline{c }=1}^{c-1}{n}_{\mathrm{\Omega }_{\mathrm{k}_{2\overline{c}}}} }^{\mathrm{\Omega }_{\mathrm{k}_{2c}}}dudv$$

See Equation S49 for the expansion of Eq. 48.

For $${n}_\mathrm{{\Omega }_{d}}+{n}_\mathrm{{\Omega }_{s}}+\sum_{\overline{c }=1}^{{n}_\mathrm{crs}}{n}_{\mathrm{\Omega }_{\mathrm{k}_{1\overline{c}}} }+\sum_{\overline{c }=1}^{c-1}{n}_{\mathrm{\Omega }_{\mathrm{k}_{2\overline{c}}} }<a\le {n}_\mathrm{{\Omega }_{d}}+{n}_\mathrm{{\Omega }_{s}}+\sum_{\overline{c }=1}^{{n}_\mathrm{crs}}{n}_{\mathrm{\Omega }_{\mathrm{k}_{1\overline{c}}} }+\sum_{\overline{c }=1}^{c}{n}_{\mathrm{\Omega }_{\mathrm{k}_{2\overline{c}}} }$$ and $${n}_\mathrm{{\Omega }_{d}}+{n}_\mathrm{{\Omega }_{s}}+\sum_{\overline{c }=1}^{{n}_\mathrm{crs}}{n}_{\mathrm{\Omega }_{\mathrm{k}_{1\overline{c}}} }+\sum_{\overline{c }=1}^{c-1}{n}_{\mathrm{\Omega }_{\mathrm{k}_{2\overline{c}}} }<b\le {n}_\mathrm{{\Omega }_{d}}+{n}_\mathrm{{\Omega }_{s}}+\sum_{\overline{c }=1}^{{n}_\mathrm{crs}}{n}_{\mathrm{\Omega }_{\mathrm{k}_{1\overline{c}}} }+\sum_{\overline{c }=1}^{c}{n}_{\mathrm{\Omega }_{\mathrm{k}_{2\overline{c}}} }$$,

49 $${\frac{{\partial G}_{a}}{\partial {d}_{b}^\mathrm{dof}}}_{\left(ab\right)}=\int \begin{array}{c}\left({\mathcal{F}}_{c,{K}_{2c}{K}_{2c}}^\mathrm{crs} {K}_{2c,{k}_{2c\left(b-{n}_\mathrm{{\Omega }_{d}}-{n}_\mathrm{{\Omega }_{s}}-\sum_{\overline{c }=1}^{{n}_\mathrm{crs}}{n}_{\mathrm{\Omega }_{\mathrm{k}_{1\overline{c}}} }-\sum_{\overline{c }=1}^{c-1}{n}_{\mathrm{\Omega }_{\mathrm{k}_{2\overline{c}}} }\right)}^{\mathrm{\Omega }_{\mathrm{k}_{2c}}}} {N}_{a-{n}_\mathrm{{\Omega }_{d}}-{n}_\mathrm{{\Omega }_{s}}-\sum_{\overline{c }=1}^{{n}_\mathrm{crs}}{n}_{\mathrm{\Omega }_{\mathrm{k}_{1\overline{c}}} }-\sum_{\overline{c }=1}^{c-1}{n}_{\mathrm{\Omega }_{\mathrm{k}_{2\overline{c}}}} }^{\mathrm{\Omega }_{\mathrm{k}_{2c}}}\right)dudv\end{array}$$

For $${n}_\mathrm{{\Omega }_{d}}+{n}_\mathrm{{\Omega }_{s}}+\sum_{\overline{c }=1}^{{n}_\mathrm{crs}}{n}_{\mathrm{\Omega }_{\mathrm{k}_{1\overline{c}}} }+\sum_{\overline{c }=1}^{c-1}{n}_{\mathrm{\Omega }_{\mathrm{k}_{2\overline{c}}} }<a\le {n}_\mathrm{{\Omega }_{d}}+{n}_\mathrm{{\Omega }_{s}}+\sum_{\overline{c }=1}^{{n}_\mathrm{crs}}{n}_{\mathrm{\Omega }_{\mathrm{k}_{1\overline{c}}} }+\sum_{\overline{c }=1}^{c}{n}_{\mathrm{\Omega }_{\mathrm{k}_{2\overline{c}}} }$$ and $$b={n}_\mathrm{{\Omega }_{d}}+{n}_\mathrm{{\Omega }_{s}}+\sum_{\overline{c }=1}^{{n}_\mathrm{crs}}{n}_{\mathrm{\Omega }_{\mathrm{k}_{1\overline{c}}} }+\sum_{\overline{c }=1}^{{n}_\mathrm{crs}}{n}_{\mathrm{\Omega }_{\mathrm{k}_{2\overline{c}}} }+1+c$$,

50 $${\frac{{\partial G}_{a}}{\partial {d}_{b}^\mathrm{dof}}}_{\left(ab\right)}=\int \begin{array}{c}\left({\mathcal{F}}_{c,{K}_{2c}{\lambda }_{b-{n}_\mathrm{{\Omega }_{d}}-{n}_\mathrm{{\Omega }_{s}}-\sum_{\overline{c }=1}^{{n}_\mathrm{crs}}{n}_{\mathrm{\Omega }_{\mathrm{k}_{1\overline{c}}} }-\sum_{\overline{c }=1}^{{n}_\mathrm{crs}}{n}_{\mathrm{\Omega }_{\mathrm{k}_{2\overline{c}}} }-1}^\mathrm{crs}}^\mathrm{crs-cstr} {N}_{a-{n}_\mathrm{{\Omega }_{d}}-{n}_ \mathrm{{\Omega }_{s}}-\sum_{\overline{c }=1}^{{n}_\mathrm{crs}}{n}_{\mathrm{\Omega }_{\mathrm{k}_{1\overline{c}}} }-\sum_{\overline{c }=1}^{c-1}{n}_{\mathrm{\Omega }_{\mathrm{k}_{2\overline{c}}}} }^{\mathrm{\Omega }_{\mathrm{k}_{2c}}}\right)dudv\end{array}$$

For $$a={n}_\mathrm{{\Omega }_{d}}+{n}_\mathrm{{\Omega }_{s}}+\sum_{\overline{c }=1}^{{n}_\mathrm{crs}}{n}_{\mathrm{\Omega }_{\mathrm{k}_{1\overline{c}}} }+\sum_{\overline{c }=1}^{{n}_\mathrm{crs}}{n}_{\mathrm{\Omega }_{\mathrm{k}_{2\overline{c}}} }+1$$ and $$b\le {n}_\mathrm{{\Omega }_{d}}$$

51 $${\frac{{\partial G}_{a}}{\partial {d}_{b}^\mathrm{dof}}}_{\left(ab\right)}=\int \left({{\mathcal{F}}_{,{\lambda }^\mathrm{lipid}{D}_{n}}^\mathrm{lipid-cstr}}_{\left(1n\right)}\right)\begin{array}{c}\left({{D}_{n,{d}_{b}^\mathrm{{\Omega }_{d}}}}_{\left(n1\right)}\right)dudv\end{array}$$

See Equation S50 for the expansion of Eq. 51.

For $$a={n}_\mathrm{{\Omega }_{d}}+{n}_\mathrm{{\Omega }_{s}}+\sum_{\overline{c }=1}^{{n}_\mathrm{crs}}{n}_{\mathrm{\Omega }_{\mathrm{k}_{1\overline{c}}} }+\sum_{\overline{c }=1}^{{n}_\mathrm{crs}}{n}_{\mathrm{\Omega }_{\mathrm{k}_{2\overline{c}}} }+1$$ and $${n}_\mathrm{{\Omega }_{d}}<b\le {n}_\mathrm{{\Omega }_{d}}+{n}_\mathrm{{\Omega }_{s}}$$

52 $${\frac{{\partial G}_{a}}{\partial {d}_{b}^\mathrm{dof}}}_{\left(ab\right)}=\int {\mathcal{F}}_{,{\lambda }^\mathrm{lipid}\alpha }^\mathrm{lipid-cstr } \begin{array}{c}{\alpha }_{,{s}_{b-{n}_\mathrm{{\Omega }_{d}}}^\mathrm{{\Omega }_{s}}}dudv\end{array}$$

For $$a={n}_\mathrm{{\Omega }_{d}}+{n}_\mathrm{{\Omega }_{s}}+\sum_{\overline{c }=1}^{{n}_\mathrm{crs}}{n}_{\mathrm{\Omega }_{\mathrm{k}_{1\overline{c}}} }+\sum_{\overline{c }=1}^{{n}_\mathrm{crs}}{n}_{\mathrm{\Omega }_{\mathrm{k}_{2\overline{c}}} }+1+c$$ and $$b\le {n}_\mathrm{{\Omega }_{d}}$$,

53 $${\frac{{\partial G}_{a}}{\partial {d}_{b}^\mathrm{dof}}}_{\left(ab\right)}=\int \left({{\mathcal{F}}_{c,{\lambda }_{c}^\mathrm{crs}{D}_{n}}^\mathrm{crs-cstr}}_{\left(1n\right)}\right)\left({{D}_{n,{d}_{b}^\mathrm{{\Omega }_{d}}}}_{(n1)}\right)dudv$$

See Equation S51 for the expansion of Eq. 53.

For $$a={n}_\mathrm{{\Omega }_{d}}+{n}_\mathrm{{\Omega }_{s}}+\sum_{\overline{c }=1}^{{n}_\mathrm{crs}}{n}_{\mathrm{\Omega }_{\mathrm{k}_{1\overline{c}}} }+\sum_{\overline{c }=1}^{{n}_\mathrm{crs}}{n}_{\mathrm{\Omega }_{\mathrm{k}_{2\overline{c}}} }+1+c$$ and $${n}_\mathrm{{\Omega }_{d}}+{n}_\mathrm{{\Omega }_{s}}+\sum_{\overline{c }=1}^{c-1}{n}_{\mathrm{\Omega }_{\mathrm{k}_{1\overline{c}}} }<b\le {n}_\mathrm{{\Omega }_{d}}+{n}_\mathrm{{\Omega }_{s}}+\sum_{\overline{c }=1}^{c}{n}_{\mathrm{\Omega }_{\mathrm{k}_{1\overline{c}}} }$$,

54 $${\frac{{\partial G}_{a}}{\partial {d}_{b}^\mathrm{dof}}}_{\left(ab\right)}=\int \begin{array}{c}\left({\mathcal{F}}_{c,{\lambda }_{c}^\mathrm{crs}{K}_{1c}}^\mathrm{crs-cstr} {K}_{1c,{k}_{1c\left(b-{n}_\mathrm{{\Omega }_{d}}-{n}_\mathrm{{\Omega }_{s}}-\sum_{\overline{c }=1}^{c-1}{n}_{\mathrm{\Omega }_{\mathrm{k}_{1\overline{c}}} }\right)}^{\mathrm{\Omega }_{\mathrm{k}_{1c}}}}\right)dudv\end{array}$$

For $$a={n}_\mathrm{{\Omega }_{d}}+{n}_\mathrm{{\Omega }_{s}}+\sum_{\overline{c }=1}^{{n}_\mathrm{crs}}{n}_{\mathrm{\Omega }_{\mathrm{k}_{1\overline{c}}} }+\sum_{\overline{c }=1}^{{n}_\mathrm{crs}}{n}_{\mathrm{\Omega }_{\mathrm{k}_{2\overline{c}}} }+1+c$$ and $${n}_\mathrm{{\Omega }_{d}}+{n}_\mathrm{{\Omega }_{s}}+\sum_{\overline{c }=1}^{{n}_\mathrm{crs}}{n}_{\mathrm{\Omega }_{\mathrm{k}_{1\overline{c}}} }+\sum_{\overline{c }=1}^{c-1}{n}_{\mathrm{\Omega }_{\mathrm{k}_{2\overline{c}}} }<b\le {n}_\mathrm{{\Omega }_{d}}+{n}_\mathrm{{\Omega }_{s}}+\sum_{\overline{c }=1}^{{n}_\mathrm{crs}}{n}_{\mathrm{\Omega }_{\mathrm{k}_{1\overline{c}}} }+\sum_{\overline{c }=1}^{c}{n}_{\mathrm{\Omega }_{\mathrm{k}_{2\overline{c}}} }$$,

55 $${\frac{{\partial G}_{a}}{\partial {d}_{b}^\mathrm{dof}}}_{\left(ab\right)}=\int \begin{array}{c}\left({\mathcal{F}}_{c,{\lambda }_{c}^\mathrm{crs}{K}_{2c}}^\mathrm{crs-cstr} {K}_{2c,{k}_{2c\left(b-{n}_\mathrm{{\Omega }_{d}}-{n}_\mathrm{{\Omega }_{s}}-\sum_{\overline{c }=1}^{{n}_\mathrm{crs}}{n}_{\mathrm{\Omega }_{\mathrm{k}_{1\overline{c}}} }-\sum_{\overline{c }=1}^{c-1}{n}_{\mathrm{\Omega }_{\mathrm{k}_{2\overline{c}}} }\right)}^{\mathrm{\Omega }_{\mathrm{k}_{2c}}}}\right)dudv\end{array}$$

Note that the function evaluation and integration in Eqs. 29‒34 and 36‒55 can only be performed locally for the corresponding elements, since the unknown variable of each element is associated with the element shape functions locally in finite element methods.

### Function evaluation for elements in the residual vector and the Jacobian matrix

To evaluate the residual vector and the Jacobian matrix, the functions ${X}^\mathrm{e}$, ${Y}^\mathrm{e}$, ${Z}^\mathrm{e}$, ${\alpha }^\mathrm{e}$, ${K}_{1c}^\mathrm{e}$, and ${K}_{2c}^\mathrm{e}$ can be defined as given in Eqs. 56‒61 from the element’s point of view.

56 $${X}^\mathrm{e}=\sum_{{i}_\mathrm{e}=1}^{9}{B}_{10-{i}_\mathrm{e}}{d}_{{i}_\mathrm{e}}^\mathrm{e}\mathrm{cos}\left({\theta }_{{i}_\mathrm{e}}^\mathrm{eX}\right)$$

57 $${Y}^\mathrm{e}=\sum_{{i}_\mathrm{e}=1}^{9}{B}_{10-{i}_\mathrm{e}}{d}_{{i}_\mathrm{e}}^\mathrm{e}\mathrm{cos}\left({\theta }_{{i}_\mathrm{e}}^\mathrm{eY}\right)$$

58 $${Z}^\mathrm{e}=\sum_{{i}_\mathrm{e}=1}^{9}{B}_{10-{i}_\mathrm{e}}{d}_{{i}_\mathrm{e}}^\mathrm{e}\mathrm{cos}\left({\theta }_{{i}_\mathrm{e}}^\mathrm{eZ}\right)$$

59 $${\alpha }^\mathrm{e}=\sum_{{i}_\mathrm{e}=1}^{9}{B}_{10-{i}_\mathrm{e}}{s}_{{i}_\mathrm{e}}^\mathrm{e}$$

60 $${K}_{1c}^\mathrm{e}=\sum_{{i}_\mathrm{e}=1}^{9}{B}_{10-{i}_\mathrm{e}}{k}_{{1ci}_\mathrm{e}}^\mathrm{e}$$

and

61 $${K}_{2c}^\mathrm{e}=\sum_{{i}_\mathrm{e}=1}^{9}{B}_{10-{i}_\mathrm{e}}{k}_{{2ci}_\mathrm{e}}^\mathrm{e}$$

*B*_1 _to *B*_9 _are the nine basis B-spline surface functions in the parent domain defined by $\xi \in \left[-\mathrm{1,1}\right]$ and $\eta \in \left[-\mathrm{1,1}\right]$ (see Eqs. S31‒S39 and Fig. S1D). ${d}_{{i}_\mathrm{e}}^\mathrm{e}$ (Fig. S1B), ${s}_{{i}_\mathrm{e}}^\mathrm{e}\text{, }{k}_{{1ci}_\mathrm{e}}^\mathrm{e}\text{, }{k}_{{2ci}_\mathrm{e}}^\mathrm{e}$ are the unknowns. $\mathrm{cos}\left({\theta }_{{i}_\mathrm{e}}^\mathrm{eX}\right)\text{, }\mathrm{cos}\left({\theta }_{{i}_\mathrm{e}}^\mathrm{eY}\right)\text{, }\mathrm{cos}\left({\theta }_{{i}_\mathrm{e}}^\mathrm{eZ}\right)$ can be calculated from $\mathrm{cos}\left({\theta }_{{i}_\mathrm{e}}^\mathrm{eX}\right)=\frac{{\widehat{n}}_\mathrm{X}}{\sqrt{{{\widehat{n}}_\mathrm{X}}^{2}+{{\widehat{n}}_\mathrm{Y}}^{2}+{{\widehat{n}}_\mathrm{Z}}^{2}}}\text{, }\mathrm{cos}\left({\theta }_{{i}_\mathrm{e}}^\mathrm{eY}\right)=\frac{{\widehat{n}}_\mathrm{Y}}{\sqrt{{{\widehat{n}}_\mathrm{X}}^{2}+{{\widehat{n}}_\mathrm{Y}}^{2}+{{\widehat{n}}_\mathrm{Z}}^{2}}}\text{, }\mathrm{cos}\left({\theta }_{{i}_\mathrm{e}}^\mathrm{eZ}\right)=\frac{{\widehat{n}}_\mathrm{Z}}{\sqrt{{{\widehat{n}}_\mathrm{X}}^{2}+{{\widehat{n}}_\mathrm{Y}}^{2}+{{\widehat{n}}_\mathrm{Z}}^{2}}}$, respectively, at the center of the corresponding element. To perform the numerical integration, the $5\times\:5$ Gaussian quadrature rule was used. Therefore, the B-spline functions and their derivatives per element were pre-calculated at twenty-five Gauss quadrature points.

### Iterative calculations

With the given boundary values ${x}_{{j}_\mathrm{d}}^\mathrm{{\Gamma }_{d}}$, ${y}_{{j}_\mathrm{d}}^\mathrm{{\Gamma }_{d}}$, ${z}_{{j}_\mathrm{d}}^\mathrm{{\Gamma }_{d}}$, ${s}_{{j}_\mathrm{d}}^\mathrm{{\Gamma }_{d}}$, ${k}_{1c{j}_{\mathrm{k}_{1c}}}^{\mathrm{\Gamma }_{\mathrm{k}_{1c}}}$, ${k}_{2c{j}_{\mathrm{k}_{2c}}}^{\mathrm{\Gamma }_{\mathrm{k}_{2c}}}$ and the given fixed positions ${\overline{X} }_{c}$, ${\overline{Y} }_{c}$, ${\overline{Z} }_{c}$, ${\overline{X} }_{cp}$, ${\overline{Y} }_{cp}$, ${\overline{Z} }_{cp}$ for crosslinkers, initial guesses for the unknowns ${d}_{{i}_\mathrm{d}}^\mathrm{{\Omega }_{d}}$, ${s}_{{i}_\mathrm{s}}^\mathrm{{\Omega }_{s}}$, ${k}_{1c{i}_{\mathrm{k}_{1c}}}^{\mathrm{\Omega }_{\mathrm{k}_{1c}}}$, ${k}_{2c{i}_{\mathrm{k}_{2c}}}^{\mathrm{\Omega }_{\mathrm{k}_{2c}}}$, ${\lambda }^\mathrm{lipid}$, ${\lambda }_{c}^\mathrm{crs}$ were substituted into the residual vector $\mathcal{G}$ and the Jacobian matrix $\mathcal{J}$. The vector of unknowns ${\mathop{d}\limits^{\rightharpoonup}}_{{i_\mathrm{Newton} }}$ can be defined and calculated from ${\mathop{d}\limits^{\rightharpoonup}}_{{i_\mathrm{Newton}+1}} = {\mathop{d}\limits^{\rightharpoonup}}_{{i_\mathrm{Newton}}} - {\mathcal{J}}\left( {{\mathop{d}\limits^{\rightharpoonup}}_{{i_\mathrm{Newton} }}} \right)^{ - 1} {\mathcal{G}}\left( {{\mathop{d}\limits^{\rightharpoonup}}_{{i_\mathrm{Newton} }}} \right)$, where ${i}_\mathrm{Newton}$ is the iteration index in the Newton–Raphson method. This iterative process continued until the Euclidean norms of the difference between two consecutive solutions for ${d}_{{i}_\mathrm{d}}^\mathrm{{\Omega }_{d}}$, ${s}_{{i}_\mathrm{s}}^\mathrm{{\Omega }_{s}}$, ${k}_{1c{i}_{\mathrm{k}_{1c}}}^{\mathrm{\Omega }_{\mathrm{k}_{1c}}}$, ${k}_{2c{i}_{\mathrm{k}_{2c}}}^{\mathrm{\Omega }_{\mathrm{k}_{2c}}}$, ${\lambda }^\mathrm{lipid}$, ${\lambda }_{c}^\mathrm{crs}$ became smaller than predefined values. With the given variational modulus density theory for the membrane-crosslinker complex, the presented nonlinear finite element method based on the Newton–Raphson method provides stable convergence and a unique solution, even in the presence of a logarithmic singularity and negative force-extension stiffness, as exemplified in Fig. 3A and Fig. 4F.

### Forces applied on the fixed point for crosslinkers

The forces applied on the fixed point (red bead in Fig. 1) of crosslinkers are defined by taking the derivative of the total energy with respect to ${\overline{X} }_{c}$, ${\overline{Y} }_{c}$, and ${\overline{Z} }_{c}$ (or ${\overline{X} }_{cp}$, ${\overline{Y} }_{cp}$, and ${\overline{Z} }_{cp}$). The $X$, $Y$, and $Z$ components of the force on the crosslinker indexed by $c$ are expressed as follows, respectively.

62 $${f}_{{\overline{\mathrm{X}} }_{c}}=\frac{{\partial \Pi }^\mathrm{totE}}{\partial {\overline{X} }_{c}}=-\int {M}_{c}\left(X-{\overline{X} }_{c}\right)\sqrt{{\left(X-{\overline{X} }_{c}\right)}^{2}+{\left(Y-{\overline{Y} }_{c}\right)}^{2}+{\left(Z-{\overline{Z} }_{c}\right)}^{2}}dA$$

63 $${f}_{{\overline{\mathrm{Y}} }_{c}}=\frac{{\partial \Pi }^\mathrm{totE}}{\partial {\overline{Y} }_{c}}=-\int {M}_{c}\left(Y-{\overline{Y} }_{c}\right)\sqrt{{\left(X-{\overline{X} }_{c}\right)}^{2}+{\left(Y-{\overline{Y} }_{c}\right)}^{2}+{\left(Z-{\overline{Z} }_{c}\right)}^{2}}dA$$

64 $${f}_{{\overline{\mathrm{Z}} }_{c}}=\frac{{\partial \Pi }^\mathrm{totE}}{\partial {\overline{Z} }_{c}}=-\int {M}_{c}\left(Z-{\overline{Z} }_{c}\right)\sqrt{{\left(X-{\overline{X} }_{c}\right)}^{2}+{\left(Y-{\overline{Y} }_{c}\right)}^{2}+{\left(Z-{\overline{Z} }_{c}\right)}^{2}}dA$$

where ${\Pi }^{\mathrm{totE}}=\int {E}^{\mathrm{curv}}{dA}+\int {E}^{\mathrm{st}}{dA}+\sum_{{c}=1}^{{n}_{\mathrm{crs}}}\int {E}_{{c}}^{\mathrm{crs}}{dA}$ (see Eqs. 1‒6). The total crosslinker force is given by ${f}_{c}=\sqrt{{{f}_{{\overline{\mathrm{X}} }_{c}}}^{2}+{{f}_{{\overline{\mathrm{Y}} }_{c}}}^{2}+{{f}_{{\overline{\mathrm{Z}} }_{c}}}^{2}}$. The calculated solutions from the finite element model are substituted into Eqs. 62‒64 to compute the force values. The *X*, *Y*, *Z*, and total forces on the crosslinker indexed by $c$ and $p$ can be similarly calculated, respectively, as follows.

65 $${f}_{{\overline{\mathrm{X}} }_{cp}}=-\int {{G}_{cp}^\mathrm{M}M}_{c}\left(X-{\overline{X} }_{cp}\right)\sqrt{{\left(X-{\overline{X} }_{cp}\right)}^{2}+{\left(Y-{\overline{Y} }_{cp}\right)}^{2}+{\left(Z-{\overline{Z} }_{cp}\right)}^{2}}dA$$

66 $${f}_{{\overline{\mathrm{Y}} }_{cp}}=-\int {{G}_{cp}^\mathrm{M}M}_{c}\left(Y-{\overline{Y} }_{cp}\right)\sqrt{{\left(X-{\overline{X} }_{cp}\right)}^{2}+{\left(Y-{\overline{Y} }_{cp}\right)}^{2}+{\left(Z-{\overline{Z} }_{cp}\right)}^{2}}dA$$

67 $${f}_{{\overline{\mathrm{Z}} }_{cp}}=-\int {{G}_{cp}^\mathrm{M}M}_{c}\left(Z-{\overline{Z} }_{cp}\right)\sqrt{{\left(X-{\overline{X} }_{cp}\right)}^{2}+{\left(Y-{\overline{Y} }_{cp}\right)}^{2}+{\left(Z-{\overline{Z} }_{cp}\right)}^{2}}dA$$

68 $${f}_{cp}=\sqrt{{{f}_{{\overline{\mathrm{X}} }_{cp}}}^{2}+{{f}_{{\overline{\mathrm{Y}} }_{cp}}}^{2}+{{f}_{{\overline{\mathrm{Z}} }_{cp}}}^{2}}$$

### Derivation of Eq. 7

From Eq. 5, the elastic energy for talin with two fixed ends can be written as follows:

69 $${\Pi }^\mathrm{fixed-talin}=\frac{1}{3}{M}_\mathrm{fixed-talin}{A}_\mathrm{talin}{{\Delta L}_\mathrm{talin}}^{3}$$

where ${M}_\mathrm{fixed-talin}$ and ${A}_\mathrm{talin}$ are the elastic modulus density of talin and the cross-section area of talin, respectively. This area mimics a point-like interaction, so the relation $\varphi =\int \left({K}_{1}+{K}_{2}\right)dA$ can be rewritten as ${\varphi }_\mathrm{fixed-talin}=\left({K}_{1}+{K}_{2}\right){A}_\mathrm{talin}$. To minimize ${\Pi }^\mathrm{fixed-talin}$ by minimizing ${M}_\mathrm{fixed-talin}$, we can use ${K}_{1}/{K}_{2}=1$. Then, ${\varphi }_\mathrm{fixed-talin}=\left({K}_{1}+{K}_{2}\right){A}_\mathrm{talin}={2{K}_{1}A}_\mathrm{talin}$. Therefore, ${M}_\mathrm{fixed-talin}$ can be written as follows:

70 $${M}_\mathrm{fixed-talin}={{K}_{1}}^{2}+{{K}_{2}}^{2}=2{{K}_{1}}^{2}=\frac{{{\varphi }_\mathrm{fixed-talin}}^{2}}{2{{A}_\mathrm{talin}}^{2}}$$

By substituting Eq. 70 into Eq. 69 and taking the derivative of Eq. 69 with respect to ${\Delta L}_\mathrm{talin}$, the force for the fixed talin can be defined as follows:

71 $${f}_\mathrm{fixed-talin}=\frac{{{\varphi }_\mathrm{fixed-talin}}^{2}{{\Delta L}_\mathrm{talin}}^{2}}{2{A}_\mathrm{talin}}$$

### Justification of constant ${G}_{cp}^\mathrm{M}$

As similarly done for deriving Eq. 7, the justification for the constant ${G}_{cp}^\mathrm{M}$ can be established by introducing an intrinsic constant ${\varphi }_\mathrm{elastic-ratio}$. The underlying assumption is that the elastic property ratio (i.e., ${R}_\mathrm{elastic-ratio}$) between two linked crosslinkers during confined diffusion is governed by the intrinsic constant (i.e., ${\varphi }_\mathrm{elastic-ratio}={K}_{1}+{K}_{2}$ . The ratio ${R}_\mathrm{elastic-ratio}={{K}_{1}}^{2}+{{K}_{2}}^{2}$ is minimum and a fixed constant, when

${K}_{1}/{K}_{2}$ = 1. Since the two crosslinkers are connected during confined diffusion, ${G}_{cp}^\mathrm{M}$ can be also considered a constant.

### Programing and simulation environments

The model was implemented using MATLAB R2022a, along with its Curve Fitting Toolbox and Symbolic Math Toolbox. The code was parallelized using MATLAB’s Parallel Computing Toolbox. For data analyses, the function central_diff.m (developed by Robert A. Canfield) from the MATLAB Center File Exchange was also employed. The computations were carried out on a Windows 11 Home system with an Intel Core i9-10980XE processor (18 cores) and 128 GB of usable memory.

## Code availability

MATLAB codes for the finite element model and data analysis are available from 10.6084/m9.figshare.28089794.
