# Publisher Correction: The variational modulus density theory explains mechanical responses of cell membranes and membrane crosslinkers

**DOI:** 10.1038/s41598-025-30700-3

**Published:** 2026-01-08

**Authors:** Jichul Kim

**Affiliations:** Department of Biomedical Engineering, Dongguk University, Seoul, 04620 Republic of Korea

Correction to: *Scientific Reports* 10.1038/s41598-025-17573-2, published online 16 October 2025

The original version of this Article contained errors in the Results and Appendix.

In the Results’ section, under the subheading ‘The correlation of the variational modulus density theory to current theories in classical elasticity’,

“Accordingly, as the best way, ${\varphi }_{\text{Fixed}-\text{talin}}$ might be enough to maximize the size of the set of possible values (see APPENDIX for the derivation of Eq. 7).”

now reads:

“Accordingly, as the best way, ${\varphi }_{\text{Fixed}-\text{talin}}$ is decomposed to generate multiple variations of the modulus density value. As similarly done for the mobile crosslinker, using two variables to decompose ${\varphi }_{\text{Fixed}-\text{talin}}$ might be enough to maximize the size of the set of possible values (see APPENDIX for the derivation of Eq. 7).”

In the Appendix, under the subheading ‘Justification of constant ${G}_{cp}^{\text{M}}$,

“As similarly done for deriving Eq. 7, the justification for the constant ${G}_{cp}^{\text{M}}$ can be established by introducing an intrinsic constant ${\varphi }_{\text{elasticratio}}$.”

now reads:

“As similarly done for deriving Eq. 7, the justification for the constant ${G}_{cp}^{\text{M}}$ can be established by introducing an intrinsic constant ${\varphi }_{\text{elastic}-\text{ratio}}$.”

The original Article has been corrected.

