# Supplementary material for: The variational modulus density theory explains mechanical responses of cell membranes and membrane crosslinkers
Source: Sci Rep. 2025 Oct 16;15:36154. doi: 10.1038/s41598-025-17573-2 (PMC12533249; doi:10.1038/s41598-025-17573-2)
Supplement: Supplementary file 1 — Supplementary Information 1. [file 41598_2025_17573_MOESM1_ESM.pdf]

Supplementary Material (SM) for

The variational modulus density theory explains mechanical responses of cell membranes and membrane crosslinkers

Jichul Kim[1,*] (ORCID: 0000-0001-9714-3048)

[1]Department of Biomedical Engineering, Dongguk University, Seoul 04620, Republic of Korea

*Contact: jichul0kim@gmail.com

Supplementary Equation

Parametric derivatives

Parametric derivatives with respect to the parametric coordinates u and v are summarized below. The comma symbol denotes partial derivatives.

$$X_{,Y} = \frac{1}{2}\left(\frac{X_{,u}}{Y_{,u}} + \frac{X_{,v}}{Y_{,v}}\right) \quad \text{(S1)}$$

$$X_{,Z} = \frac{1}{2}\left(\frac{X_{,u}}{Z_{,u}} + \frac{X_{,v}}{Z_{,v}}\right) \quad \text{(S2)}$$

$$Y_{,X} = \frac{1}{2}\left(\frac{Y_{,u}}{X_{,u}} + \frac{Y_{,v}}{X_{,v}}\right) \quad \text{(S3)}$$

$$Y_{,Z} = \frac{1}{2}\left(\frac{Y_{,u}}{Z_{,u}} + \frac{Y_{,v}}{Z_{,v}}\right) \quad \text{(S4)}$$

$$Z_{,X} = \frac{1}{2}\left(\frac{Z_{,u}}{X_{,u}} + \frac{Z_{,v}}{X_{,v}}\right) \quad \text{(S5)}$$

$$Z_{,Y} = \frac{1}{2}\left(\frac{Z_{,u}}{Y_{,u}} + \frac{Z_{,v}}{Y_{,v}}\right) \quad \text{(S6)}$$

$$X_{,Yu} = \frac{0.5\left(X_{,uu}Y_{,u} - X_{,u}Y_{,uu}\right)}{{Y_{,u}}^2} + \frac{0.5\left(X_{,vu}Y_{,v} - X_{,v}Y_{,vu}\right)}{{Y_{,v}}^2} \quad \text{(S7)}$$

$$X_{,Yv} = \frac{0.5\left(X_{,uv}Y_{,u} - X_{,u}Y_{,uv}\right)}{{Y_{,u}}^2} + \frac{0.5\left(X_{,vv}Y_{,v} - X_{,v}Y_{,vv}\right)}{{Y_{,v}}^2} \quad \text{(S8)}$$

$$X_{,Zu} = \frac{0.5\left(X_{,uu}Z_{,u} - X_{,u}Z_{,uu}\right)}{{Z_{,u}}^2} + \frac{0.5\left(X_{,vu}Z_{,v} - X_{,v}Z_{,vu}\right)}{{Z_{,v}}^2} \quad \text{(S9)}$$

$$X_{,Zv} = \frac{0.5\left(X_{,uv}Z_{,u} - X_{,u}Z_{,uv}\right)}{{Z_{,u}}^2} + \frac{0.5\left(X_{,vv}Z_{,v} - X_{,v}Z_{,vv}\right)}{{Z_{,v}}^2} \quad \text{(S10)}$$

$$Y_{,Zu} = \frac{0.5\left(Y_{,uu}Z_{,u} - Y_{,u}Z_{,uu}\right)}{{Z_{,u}}^2} + \frac{0.5\left(Y_{,vu}Z_{,v} - Y_{,v}Z_{,vu}\right)}{{Z_{,v}}^2} \quad \text{(S11)}$$

$$Y_{,Zv} = \frac{0.5\left(Y_{,uv}Z_{,u} - Y_{,u}Z_{,uv}\right)}{{Z_{,u}}^2} + \frac{0.5\left(Y_{,vv}Z_{,v} - Y_{,v}Z_{,vv}\right)}{{Z_{,v}}^2} \quad \text{(S12)}$$

$$Y_{,Xu} = \frac{0.5\left(Y_{,uu}X_{,u} - Y_{,u}X_{,uu}\right)}{X_{,u}{}^{2}} + \frac{0.5\left(Y_{,vu}X_{,v} - Y_{,v}X_{,vu}\right)}{X_{,v}{}^{2}} \quad \text{(S13)}$$

$$Y_{,Xv} = \frac{0.5\left(Y_{,uv}X_{,u} - Y_{,u}X_{,uv}\right)}{X_{,u}{}^{2}} + \frac{0.5\left(Y_{,vv}X_{,v} - Y_{,v}X_{,vv}\right)}{X_{,v}{}^{2}} \quad \text{(S14)}$$

$$Z_{,Xu} = \frac{0.5\left(Z_{,uu}X_{,u} - Z_{,u}X_{,uu}\right)}{X_{,u}{}^{2}} + \frac{0.5\left(Z_{,vu}X_{,v} - Z_{,v}X_{,vu}\right)}{X_{,v}{}^{2}} \quad \text{(S15)}$$

$$Z_{,Xv} = \frac{0.5\left(Z_{,uv}X_{,u} - Z_{,u}X_{,uv}\right)}{X_{,u}{}^{2}} + \frac{0.5\left(Z_{,vv}X_{,v} - Z_{,v}X_{,vv}\right)}{X_{,v}{}^{2}} \quad \text{(S16)}$$

$$Z_{,Yu} = \frac{0.5\left(Z_{,uu}Y_{,u} - Z_{,u}Y_{,uu}\right)}{Y_{,u}{}^{2}} + \frac{0.5\left(Z_{,vu}Y_{,v} - Z_{,v}Y_{,vu}\right)}{Y_{,v}{}^{2}} \quad \text{(S17)}$$

$$Z_{,Yv} = \frac{0.5\left(Z_{,uv}Y_{,u} - Z_{,u}Y_{,uv}\right)}{Y_{,u}{}^{2}} + \frac{0.5\left(Z_{,vv}Y_{,v} - Z_{,v}Y_{,vv}\right)}{Y_{,v}{}^{2}} \quad \text{(S18)}$$

$$X_{,YY} = \frac{1}{2}\left(\frac{X_{,Yu}}{Y_{,u}} + \frac{X_{,Yv}}{Y_{,v}}\right) \quad \text{(S19)}$$

$$X_{,ZZ} = \frac{1}{2}\left(\frac{X_{,Zu}}{Z_{,u}} + \frac{X_{,Zv}}{Z_{,v}}\right) \quad \text{(S20)}$$

$$Y_{,XX} = \frac{1}{2}\left(\frac{Y_{,Xu}}{X_{,u}} + \frac{Y_{,Xv}}{X_{,v}}\right) \quad \text{(S21)}$$

$$Y_{,ZZ} = \frac{1}{2}\left(\frac{Y_{,Zu}}{Z_{,u}} + \frac{Y_{,Zv}}{Z_{,v}}\right) \quad \text{(S22)}$$

$$Z_{,XX} = \frac{1}{2}\left(\frac{Z_{,Xu}}{X_{,u}} + \frac{Z_{,Xv}}{X_{,v}}\right) \quad \text{(S23)}$$

$$Z_{,YY} = \frac{1}{2}\left(\frac{Z_{,Yu}}{Y_{,u}} + \frac{Z_{,Yv}}{Y_{,v}}\right) \quad \text{(S24)}$$

$$Z_{,XY} = \frac{1}{2}\left(\frac{Z_{,Xu}}{Y_{,u}} + \frac{Z_{,Xv}}{Y_{,v}}\right) \quad \text{(S25)}$$

$$Z_{,YX} = \frac{1}{2}\left(\frac{Z_{,Yu}}{X_{,u}} + \frac{Z_{,Yv}}{X_{,v}}\right) \quad \text{(S26)}$$

$$X_{,YZ} = \frac{1}{2}\left(\frac{X_{,Yu}}{Z_{,u}} + \frac{X_{,Yv}}{Z_{,v}}\right) \quad \text{(S27)}$$

$$X_{,ZY} = \frac{1}{2}\left(\frac{X_{,Zu}}{Y_{,u}} + \frac{X_{,Zv}}{Y_{,v}}\right) \quad \text{(S28)}$$

$$Y_{,XZ} = \frac{1}{2}\left(\frac{Y_{,Xu}}{Z_{,u}} + \frac{Y_{,Xv}}{Z_{,v}}\right) \quad \text{(S29)}$$

$$Y_{,ZX} = \frac{1}{2}\left(\frac{Y_{,Zu}}{X_{,u}} + \frac{Y_{,Zv}}{X_{,v}}\right) \quad \text{(S30)}$$

B-spline surface functions used in this work for prior function evaluation at the Gauss quadrature points

$$B(\xi,\eta)_1 = \frac{(\xi-\xi_1)^2}{2(\xi_2-\xi_1)^2} \times \frac{(\eta-\eta_1)^2}{2(\eta_2-\eta_1)^2} \quad \text{(S31)}$$

where $(\xi_1, \xi_2) = (-1, 1)$ and $(\eta_1, \eta_2) = (-1, 1)$.

$$B(\xi,\eta)_2 = \frac{(\xi-\xi_1)^2 - 3(\xi-\xi_2)^2}{2(\xi_3-\xi_2)^2} \times \frac{(\eta-\eta_1)^2}{2(\eta_2-\eta_1)^2} \quad \text{(S32)}$$

where $(\xi_1, \xi_2, \xi_3) = (-3, -1, 1)$ and $(\eta_1, \eta_2) = (-1, 1)$.

$$B(\xi,\eta)_3 = \frac{(\xi-\xi_1)^2 - 3(\xi-\xi_2)^2 + 3(\xi-\xi_3)^2}{2(\xi_4-\xi_3)^2} \times \frac{(\eta-\eta_1)^2}{2(\eta_2-\eta_1)^2} \quad \text{(S33)}$$

where $(\xi_1, \xi_2, \xi_3, \xi_4) = (-5, -3, -1, 1)$ and $(\eta_1, \eta_2) = (-1, 1)$.

$$B(\xi,\eta)_4 = \frac{(\xi-\xi_1)^2}{2(\xi_2-\xi_1)^2} \times \frac{(\eta-\eta_1)^2 - 3(\eta-\eta_2)^2}{2(\eta_3-\eta_2)^2} \quad \text{(S34)}$$

where $(\xi_1, \xi_2) = (-1, 1)$ and $(\eta_1, \eta_2, \eta_3) = (-3, -1, 1)$.

$$B(\xi,\eta)_5 = \frac{(\xi-\xi_1)^2 - 3(\xi-\xi_2)^2}{2(\xi_3-\xi_2)^2} \times \frac{(\eta-\eta_1)^2 - 3(\eta-\eta_2)^2}{2(\eta_3-\eta_2)^2} \quad \text{(S35)}$$

where $(\xi_1, \xi_2, \xi_3) = (-3, -1, 1)$ and $(\eta_1, \eta_2, \eta_3) = (-3, -1, 1)$.

$$B(\xi,\eta)_6 = \frac{(\xi-\xi_1)^2 - 3(\xi-\xi_2)^2 + 3(\xi-\xi_3)^2}{2(\xi_4-\xi_3)^2} \times \frac{(\eta-\eta_1)^2 - 3(\eta-\eta_2)^2}{2(\eta_3-\eta_2)^2} \quad \text{(S36)}$$

where $(\xi_1, \xi_2, \xi_3, \xi_4) = (-5, -3, -1, 1)$ and $(\eta_1, \eta_2, \eta_3) = (-3, -1, 1)$.

$$B(\xi, \eta)_7 = \frac{(\xi - \xi_1)^2}{2(\xi_2 - \xi_1)^2} \times \frac{(\eta - \eta_1)^2 - 3(\eta - \eta_2)^2 + 3(\eta - \eta_3)^2}{2(\eta_4 - \eta_3)^2} \quad \text{(S37)}$$

where $(\xi_1, \xi_2) = (-1, 1)$ and $(\eta_1, \eta_2, \eta_3, \eta_4) = (-5, -3, -1, 1)$.

$$B(\xi, \eta)_8 = \frac{(\xi - \xi_1)^2 - 3(\xi - \xi_2)^2}{2(\xi_3 - \xi_2)^2} \times \frac{(\eta - \eta_1)^2 - 3(\eta - \eta_2)^2 + 3(\eta - \eta_3)^2}{2(\eta_4 - \eta_3)^2} \quad \text{(S38)}$$

where $(\xi_1, \xi_2, \xi_3) = (-3, -1, 1)$ and $(\eta_1, \eta_2, \eta_3, \eta_4) = (-5, -3, -1, 1)$.

$$B(\xi, \eta)_9 = \frac{(\xi - \xi_1)^2 - 3(\xi - \xi_2)^2 + 3(\xi - \xi_3)^2}{2(\xi_4 - \xi_3)^2} \times \frac{(\eta - \eta_1)^2 - 3(\eta - \eta_2)^2 + 3(\eta - \eta_3)^2}{2(\eta_4 - \eta_3)^2} \quad \text{(S39)}$$

where $(\xi_1, \xi_2, \xi_3, \xi_4) = (-5, -3, -1, 1)$ and $(\eta_1, \eta_2, \eta_3, \eta_4) = (-5, -3 - 1, 1)$.

Expansion for the element of the residual force vector

Equation 29 can be expanded as follows.

$$G_{(a1)} = \int \left(\mathcal{F}^{\mathrm{membr-all}}_{,D_m}{}_{(m1)}\right)^T \left(\overline{N}_{m(m1)}\right) + \int \sum_{c=1}^{n_{\mathrm{crs}}} \left(\mathcal{F}^{\mathrm{crs-all}}_{c,D_m}{}_{(m1)}\right)^T \left(\overline{N}_{m(m1)}\right) dudv =$$

$$\int \begin{bmatrix} \mathcal{F}^{\text{membr-all}}_{,X,u} \\ \mathcal{F}^{\text{membr-all}}_{,X,v} \\ \mathcal{F}^{\text{curv}}_{,X,uu} \\ \mathcal{F}^{\text{curv}}_{,X,vv} \\ \mathcal{F}^{\text{curv}}_{,X,uv} \\ \mathcal{F}^{\text{curv}}_{,X,vu} \\ \mathcal{F}^{\text{membr-all}}_{,Y,u} \\ \mathcal{F}^{\text{membr-all}}_{,Y,v} \\ \mathcal{F}^{\text{curv}}_{,Y,uu} \\ \mathcal{F}^{\text{curv}}_{,Y,vv} \\ \mathcal{F}^{\text{curv}}_{,Y,uv} \\ \mathcal{F}^{\text{curv}}_{,Y,vu} \\ \mathcal{F}^{\text{membr-all}}_{,Z,u} \\ \mathcal{F}^{\text{membr-all}}_{,Z,v} \\ \mathcal{F}^{\text{curv}}_{,Z,uu} \\ \mathcal{F}^{\text{curv}}_{,Z,vv} \\ \mathcal{F}^{\text{curv}}_{,Z,uv} \\ \mathcal{F}^{\text{curv}}_{,Z,vu} \end{bmatrix}^T \begin{bmatrix} N^{\Omega_\text{d}}_{a,u} \cos\theta^\text{X}_a \\ N^{\Omega_\text{d}}_{a,v} \cos\theta^\text{X}_a \\ N^{\Omega_\text{d}}_{a,uu} \cos\theta^\text{X}_a \\ N^{\Omega_\text{d}}_{a,vv} \cos\theta^\text{X}_a \\ N^{\Omega_\text{d}}_{a,uv} \cos\theta^\text{X}_a \\ N^{\Omega_\text{d}}_{a,vu} \cos\theta^\text{X}_a \\ N^{\Omega_\text{d}}_{a,u} \cos\theta^\text{Y}_a \\ N^{\Omega_\text{d}}_{a,v} \cos\theta^\text{Y}_a \\ N^{\Omega_\text{d}}_{a,uu} \cos\theta^\text{Y}_a \\ N^{\Omega_\text{d}}_{a,vv} \cos\theta^\text{Y}_a \\ N^{\Omega_\text{d}}_{a,uv} \cos\theta^\text{Y}_a \\ N^{\Omega_\text{d}}_{a,vu} \cos\theta^\text{Y}_a \\ N^{\Omega_\text{d}}_{a,u} \cos\theta^\text{Z}_a \\ N^{\Omega_\text{d}}_{a,v} \cos\theta^\text{Z}_a \\ N^{\Omega_\text{d}}_{a,uu} \cos\theta^\text{Z}_a \\ N^{\Omega_\text{d}}_{a,vv} \cos\theta^\text{Z}_a \\ N^{\Omega_\text{d}}_{a,uv} \cos\theta^\text{Z}_a \\ N^{\Omega_\text{d}}_{a,vu} \cos\theta^\text{Z}_a \end{bmatrix} dudv + \int \sum_{c=1}^{n_\text{crs}} \begin{bmatrix} \mathcal{F}^{\text{crs}}_{c,X} \\ \mathcal{F}^{\text{crs-all}}_{c,X,u} \\ \mathcal{F}^{\text{crs-all}}_{c,X,v} \\ \mathcal{F}^{crs}_{c,Y} \\ \mathcal{F}^{\text{crs-all}}_{c,Y,u} \\ \mathcal{F}^{\text{crs-all}}_{c,Y,v} \\ \mathcal{F}^{\text{crs}}_{c,Z} \\ \mathcal{F}^{\text{crs-all}}_{c,Z,u} \\ \mathcal{F}^{\text{crs-all}}_{c,Z,v} \end{bmatrix}^T \begin{bmatrix} N^{\Omega_\text{d}}_{a} \cos\theta^\text{X}_a \\ N^{\Omega_\text{d}}_{a,u} \cos\theta^\text{X}_a \\ N^{\Omega_\text{d}}_{a,v} \cos\theta^\text{X}_a \\ N^{\Omega_\text{d}}_{a} \cos\theta^\text{Y}_a \\ N^{\Omega_\text{d}}_{a,u} \cos\theta^\text{Y}_a \\ N^{\Omega_\text{d}}_{a,v} \cos\theta^\text{Y}_a \\ N^{\Omega_\text{d}}_{a} \cos\theta^\text{Z}_a \\ N^{\Omega_\text{d}}_{a,u} \cos\theta^\text{Z}_a \\ N^{\Omega_\text{d}}_{a,v} \cos\theta^\text{Z}_a \end{bmatrix} dudv \quad \text{(S40)}$$

Expansion for the element of the Jacobian matrix

Equation 36 can be expanded as follows.

$$\frac{\partial G_a}{\partial d^{\text{dof}}_b}_{(ab)} = \int \left[\left(\mathcal{F}^{\text{membr-all}}_{,D_m D_n}{}_{(mn)} \right) \left(D_{n,d^{\Omega_\text{d}}_b}{}_{(n1)} \right) \right]^T \left(\bar{N}_{m(m1)} \right) dudv +$$

$$\int \sum_{c=1}^{n_\text{crs}} \left[\left(\mathcal{F}^{\text{crs-all}}_{c,D_m D_n}{}_{(mn)} \right) \left(D_{n,d^{\Omega_\text{d}}_b}{}_{(n1)} \right) \right]^T \left(\bar{N}_{m(m1)} \right) dudv =$$

$$\int \left[\begin{bmatrix} \mathcal{F}^{\mathrm{membr-all}}_{,X_{,u}X_{,u}} & \cdots & \mathcal{F}^{\mathrm{membr-all}}_{,X_{,u}Z_{,vu}} \\ \mathcal{F}^{\mathrm{membr-all}}_{,X_{,v}X_{,u}} & \cdots & \mathcal{F}^{\mathrm{membr-all}}_{,X_{,v}Z_{,vu}} \\ \mathcal{F}^{\mathrm{membr-all}}_{,X_{,uu}X_{,u}} & \cdots & \mathcal{F}^{\mathrm{membr-all}}_{,X_{,uu}Z_{,vu}} \\ \mathcal{F}^{\mathrm{membr-all}}_{,X_{,vv}X_{,u}} & \cdots & \mathcal{F}^{\mathrm{membr-all}}_{,X_{,vv}Z_{,vu}} \\ \mathcal{F}^{\mathrm{membr-all}}_{,X_{,uv}X_{,u}} & \cdots & \mathcal{F}^{\mathrm{membr-all}}_{,X_{,uv}Z_{,vu}} \\ \mathcal{F}^{\mathrm{membr-all}}_{,X_{,vu}X_{,u}} & \cdots & \mathcal{F}^{\mathrm{membr-all}}_{,X_{,vu}Z_{,vu}} \\ \mathcal{F}^{\mathrm{membr-all}}_{,Y_{,u}X_{,u}} & \cdots & \mathcal{F}^{\mathrm{membr-all}}_{,Y_{,u}Z_{,vu}} \\ \mathcal{F}^{\mathrm{membr-all}}_{,Y_{,v}X_{,u}} & \cdots & \mathcal{F}^{\mathrm{membr-all}}_{,Y_{,v}Z_{,vu}} \\ \mathcal{F}^{\mathrm{membr-all}}_{,Y_{,uu}X_{,u}} & \cdots & \mathcal{F}^{\mathrm{membr-all}}_{,Y_{,uu}Z_{,vu}} \\ \mathcal{F}^{\mathrm{membr-all}}_{,Y_{,vv}X_{,u}} & \cdots & \mathcal{F}^{\mathrm{membr-all}}_{,Y_{,vv}Z_{,vu}} \\ \mathcal{F}^{\mathrm{membr-all}}_{,Y_{,uv}X_{,u}} & \cdots & \mathcal{F}^{\mathrm{membr-all}}_{,Y_{,uv}Z_{,vu}} \\ \mathcal{F}^{\mathrm{membr-all}}_{,Y_{,vu}X_{,u}} & \cdots & \mathcal{F}^{\mathrm{membr-all}}_{,Y_{,vu}Z_{,vu}} \\ \mathcal{F}^{\mathrm{membr-all}}_{,Z_{,u}X_{,u}} & \cdots & \mathcal{F}^{\mathrm{membr-all}}_{,Z_{,u}Z_{,vu}} \\ \mathcal{F}^{\mathrm{membr-all}}_{,Z_{,v}X_{,u}} & \cdots & \mathcal{F}^{\mathrm{membr-all}}_{,Z_{,v}Z_{,vu}} \\ \mathcal{F}^{\mathrm{membr-all}}_{,Z_{,uu}X_{,u}} & \cdots & \mathcal{F}^{\mathrm{membr-all}}_{,Z_{,uu}Z_{,vu}} \\ \mathcal{F}^{\mathrm{membr-all}}_{,Z_{,vv}X_{,u}} & \cdots & \mathcal{F}^{\mathrm{membr-all}}_{,Z_{,vv}Z_{,vu}} \\ \mathcal{F}^{\mathrm{membr-all}}_{,Z_{,uv}X_{,u}} & \cdots & \mathcal{F}^{\mathrm{membr-all}}_{,Z_{,uv}Z_{,vu}} \\ \mathcal{F}^{\mathrm{membr-all}}_{,Z_{,vu}X_{,u}} & \cdots & \mathcal{F}^{\mathrm{membr-all}}_{,Z_{,vu}Z_{,vu}} \end{bmatrix} \begin{bmatrix} X_{,ud_b^{\Omega_\mathrm{d}}} \\ X_{,vd_b^{\Omega_\mathrm{d}}} \\ X_{,uud_b^{\Omega_\mathrm{d}}} \\ X_{,vvd_b^{\Omega_\mathrm{d}}} \\ X_{,uvd_b^{\Omega_\mathrm{d}}} \\ X_{,vud_b^{\Omega_\mathrm{d}}} \\ Y_{,ud_b^{\Omega_\mathrm{d}}} \\ Y_{,vd_b^{\Omega_\mathrm{d}}} \\ Y_{,uud_b^{\Omega_\mathrm{d}}} \\ Y_{,vvd_b^{\Omega_\mathrm{d}}} \\ Y_{,uvd_b^{\Omega_\mathrm{d}}} \\ Y_{,vud_b^{\Omega_\mathrm{d}}} \\ Z_{,ud_b^{\Omega_\mathrm{d}}} \\ Z_{,vd_b^{\Omega_\mathrm{d}}} \\ Z_{,uud_b^{\Omega_\mathrm{d}}} \\ Z_{,vvd_b^{\Omega_\mathrm{d}}} \\ Z_{,uvd_b^{\Omega_\mathrm{d}}} \\ Z_{,vud_b^{\Omega_\mathrm{d}}} \end{bmatrix} \right]^T \begin{bmatrix} N_{a,u}^{\Omega_\mathrm{d}} \cos\theta_a^{\mathrm{X}} \\ N_{a,v}^{\Omega_\mathrm{d}} \cos\theta_a^{\mathrm{X}} \\ N_{a,uu}^{\Omega_\mathrm{d}} \cos\theta_a^{\mathrm{X}} \\ N_{a,vv}^{\Omega_\mathrm{d}} \cos\theta_a^{\mathrm{X}} \\ N_{a,uv}^{\Omega_\mathrm{d}} \cos\theta_a^{\mathrm{X}} \\ N_{a,vu}^{\Omega_\mathrm{d}} \cos\theta_a^{\mathrm{X}} \\ N_{a,u}^{\Omega_\mathrm{d}} \cos\theta_a^{\mathrm{Y}} \\ N_{a,v}^{\Omega_\mathrm{d}} \cos\theta_a^{\mathrm{Y}} \\ N_{a,uu}^{\Omega_\mathrm{d}} \cos\theta_a^{\mathrm{Y}} \\ N_{a,vv}^{\Omega_\mathrm{d}} \cos\theta_a^{\mathrm{Y}} \\ N_{a,uv}^{\Omega_\mathrm{d}} \cos\theta_a^{\mathrm{Y}} \\ N_{a,vu}^{\Omega_\mathrm{d}} \cos\theta_a^{\mathrm{Y}} \\ N_{a,u}^{\Omega_\mathrm{d}} \cos\theta_a^{\mathrm{Z}} \\ N_{a,v}^{\Omega_\mathrm{d}} \cos\theta_a^{\mathrm{Z}} \\ N_{a,uu}^{\Omega_\mathrm{d}} \cos\theta_a^{\mathrm{Z}} \\ N_{a,vv}^{\Omega_\mathrm{d}} \cos\theta_a^{\mathrm{Z}} \\ N_{a,uv}^{\Omega_\mathrm{d}} \cos\theta_a^{\mathrm{Z}} \\ N_{a,vu}^{\Omega_\mathrm{d}} \cos\theta_a^{\mathrm{Z}} \end{bmatrix} dudv +$$

$$\int \sum_{c=1}^{n_{\mathrm{crs}}} \left[\begin{bmatrix} \mathcal{F}^{\mathrm{crs-all}}_{c,XX} & \cdots & \mathcal{F}^{\mathrm{crs-all}}_{c,XZ_{,v}} \\ \mathcal{F}^{\mathrm{crs-all}}_{c,X_{,u}X} & \cdots & \mathcal{F}^{\mathrm{crs-all}}_{c,X_{,u}Z_{,v}} \\ \mathcal{F}^{\mathrm{crs-all}}_{c,X_{,v}X} & \cdots & \mathcal{F}^{\mathrm{crs-all}}_{c,X_{,v}Z_{,v}} \\ \mathcal{F}^{\mathrm{crs-all}}_{c,YX} & \cdots & \mathcal{F}^{\mathrm{crs-all}}_{c,YZ_{,v}} \\ \mathcal{F}^{\mathrm{crs-all}}_{c,Y_{,u}X} & \cdots & \mathcal{F}^{\mathrm{crs-all}}_{c,Y_{,u}Z_{,v}} \\ \mathcal{F}^{\mathrm{crs-all}}_{c,Y_{,v}X} & \cdots & \mathcal{F}^{\mathrm{crs-all}}_{c,Y_{,v}Z_{,v}} \\ \mathcal{F}^{\mathrm{crs-all}}_{c,ZX} & \cdots & \mathcal{F}^{\mathrm{crs-all}}_{c,ZZ_{,v}} \\ \mathcal{F}^{\mathrm{crs-all}}_{c,Z_{,u}X} & \cdots & \mathcal{F}^{\mathrm{crs-all}}_{c,Z_{,u}Z_{,v}} \\ \mathcal{F}^{\mathrm{crs-all}}_{c,Z_{,v}X} & \cdots & \mathcal{F}^{\mathrm{crs-all}}_{c,Z_{,v}Z_{,v}} \end{bmatrix} \begin{bmatrix} X_{,d_b^{\Omega_\mathrm{d}}} \\ X_{,ud_b^{\Omega_\mathrm{d}}} \\ X_{,vd_b^{\Omega_\mathrm{d}}} \\ Y_{,d_b^{\Omega_\mathrm{d}}} \\ Y_{,ud_b^{\Omega_\mathrm{d}}} \\ Y_{,vd_b^{\Omega_\mathrm{d}}} \\ Z_{,d_b^{\Omega_\mathrm{d}}} \\ Z_{,ud_b^{\Omega_\mathrm{d}}} \\ Z_{,vd_b^{\Omega_\mathrm{d}}} \end{bmatrix} \right]^T \begin{bmatrix} N_a^{\Omega_\mathrm{d}} \cos\theta_a^{\mathrm{X}} \\ N_{a,u}^{\Omega_\mathrm{d}} \cos\theta_a^{\mathrm{X}} \\ N_{a,v}^{\Omega_\mathrm{d}} \cos\theta_a^{\mathrm{X}} \\ N_a^{\Omega_\mathrm{d}} \cos\theta_a^{\mathrm{Y}} \\ N_{a,u}^{\Omega_\mathrm{d}} \cos\theta_a^{\mathrm{Y}} \\ N_{a,v}^{\Omega_\mathrm{d}} \cos\theta_a^{\mathrm{Y}} \\ N_a^{\Omega_\mathrm{d}} \cos\theta_a^{\mathrm{Z}} \\ N_{a,u}^{\Omega_\mathrm{d}} \cos\theta_a^{\mathrm{Z}} \\ N_{a,v}^{\Omega_\mathrm{d}} \cos\theta_a^{\mathrm{Z}} \end{bmatrix} dudv \quad \text{(S41)}$$

Equation 37 can be expanded as follows.

$$\frac{\partial G_a}{\partial d_b^{\text{dof}}}_{(ab)} = \int \left[\left(\mathcal{F}^{\text{membr-st-cstr}}_{,D_m\alpha\ (m1)} \right) \alpha_{,s^{\Omega_\text{s}}_{b-n_{\Omega_\text{d}}}} \right]^T \left(\bar{N}_{m(m1)} \right) dudv =$$

$$\int \left[\begin{bmatrix} \mathcal{F}^{\text{membr-st-cstr}}_{,X,_u\alpha} \\ \mathcal{F}^{\text{membr-st-cstr}}_{,X,_v\alpha} \\ \mathcal{F}^{\text{membr-st-cstr}}_{,Y,_u\alpha} \\ \mathcal{F}^{\text{membr-st-cstr}}_{,Y,_v\alpha} \\ \mathcal{F}^{\text{membr-st-cstr}}_{,Z,_u\alpha} \\ \mathcal{F}^{\text{membr-st-cstr}}_{,Z,_v\alpha} \end{bmatrix} \alpha_{,s^{\Omega_\text{s}}_{b-n_{\Omega_\text{d}}}} \right]^T \begin{bmatrix} N^{\Omega_\text{d}}_{a,u} \cos\theta^\text{X}_a \\ N^{\Omega_\text{d}}_{a,v} \cos\theta^\text{X}_a \\ N^{\Omega_\text{d}}_{a,u} \cos\theta^\text{Y}_a \\ N^{\Omega_\text{d}}_{a,v} \cos\theta^\text{Y}_a \\ N^{\Omega_\text{d}}_{a,u} \cos\theta^\text{Z}_a \\ N^{\Omega_\text{d}}_{a,v} \cos\theta^\text{Z}_a \end{bmatrix} dudv \quad \text{(S42)}$$

Equation 38 can be further expanded as follows.

$$\frac{\partial G_a}{\partial d_b^{\text{dof}}}_{(ab)} = \int \left[\left(\mathcal{F}^{\text{crs-all}}_{c,D_m K_{1c}(m1)} \right) K_{1c,k^{\Omega_{\text{k}_{1c}}}_{1c\left(b-n_{\Omega_\text{d}}-n_{\Omega_\text{s}}-\sum_{\bar{c}=1}^{c-1} n_{\Omega_{\text{k}_{1\bar{c}}}}\right)}} \right]^T \left(\bar{N}_{m(m1)} \right) dudv =$$

$$\int \left[\begin{bmatrix} \mathcal{F}^{\text{crs}}_{c,XK_{1c}} \\ \mathcal{F}^{\text{crs-all}}_{c,X,_uK_{1c}} \\ \mathcal{F}^{\text{crs-all}}_{c,X,_vK_{1c}} \\ \mathcal{F}^{\text{crs}}_{c,YK_{1c}} \\ \mathcal{F}^{\text{crs-all}}_{c,Y,_uK_{1c}} \\ \mathcal{F}^{\text{crs-all}}_{c,Y,_vK_{1c}} \\ \mathcal{F}^{\text{crs}}_{c,ZK_{1c}} \\ \mathcal{F}^{\text{crs-all}}_{c,Z,_uK_{1c}} \\ \mathcal{F}^{\text{crs-all}}_{c,Z,_vK_{1c}} \end{bmatrix} \left[K_{1c,k^{\Omega_{\text{k}_{1c}}}_{1c\left(b-n_{\Omega_\text{d}}-n_{\Omega_\text{s}}-\sum_{\bar{c}=1}^{c-1} n_{\Omega_{\text{k}_{1\bar{c}}}}\right)}} \right] \right]^T \begin{bmatrix} N^{\Omega_\text{d}}_{a} \cos\theta^\text{X}_a \\ N^{\Omega_\text{d}}_{a,u} \cos\theta^\text{X}_a \\ N^{\Omega_\text{d}}_{a,v} \cos\theta^\text{X}_a \\ N^{\Omega_\text{d}}_{a} \cos\theta^\text{Y}_a \\ N^{\Omega_\text{d}}_{a,u} \cos\theta^\text{Y}_a \\ N^{\Omega_\text{d}}_{a,v} \cos\theta^\text{Y}_a \\ N^{\Omega_\text{d}}_{a} \cos\theta^\text{Z}_a \\ N^{\Omega_\text{d}}_{a,u} \cos\theta^\text{Z}_a \\ N^{\Omega_\text{d}}_{a,v} \cos\theta^\text{Z}_a \end{bmatrix} dudv \quad \text{(S43)}$$

Equation 39 can be further expanded as follows.

$$\frac{\partial G_a}{\partial d_b^{\text{dof}}}_{(ab)} = \int \left[\left(\mathcal{F}^{\text{crs-all}}_{c,D_m K_{2c}(m1)} \right) K_{2c,k^{\Omega_{\text{k}_{2c}}}_{2c\left(b-n_{\Omega_\text{d}}-n_{\Omega_\text{s}}-\sum_{\bar{c}=1}^{n^{\text{crs}}} n_{\Omega_{\text{k}_{1\bar{c}}}}-\sum_{\bar{c}=1}^{c-1} n_{\Omega_{\text{k}_{2\bar{c}}}}\right)}} \right]^T \left(\bar{N}_{m(m1)} \right) dudv =$$

$$\int \left[\begin{bmatrix} \mathcal{F}^{\mathrm{crs}}_{c,XK_{2c}} \\ \mathcal{F}^{\mathrm{crs-all}}_{c,X_{,u}K_{2c}} \\ \mathcal{F}^{\mathrm{crs-all}}_{c,X_{,v}K_{2c}} \\ \mathcal{F}^{\mathrm{crs}}_{c,YK_{2c}} \\ \mathcal{F}^{\mathrm{crs-all}}_{c,Y_{,u}K_{2c}} \\ \mathcal{F}^{\mathrm{crs-all}}_{c,Y_{,v}K_{2c}} \\ \mathcal{F}^{\mathrm{crs}}_{c,ZK_{2c}} \\ \mathcal{F}^{\mathrm{crs-all}}_{c,Z_{,u}K_{2c}} \\ \mathcal{F}^{\mathrm{crs-all}}_{c,Z_{,v}K_{2c}} \end{bmatrix} \left[K_{2c,k_{2c\left(b-n_{\Omega_{\mathrm{d}}}-n_{\Omega_{\mathrm{S}}}-\sum_{\bar{c}=1}^{n^{\mathrm{crs}}} n_{\Omega_{\mathrm{k}_{1\bar{c}}}}-\sum_{\bar{c}=1}^{c-1} n_{\Omega_{\mathrm{k}_{2\bar{c}}}}\right)}}^{\Omega_{\mathrm{k}_{2c}}} \right] \right]^T \begin{bmatrix} N_a^{\Omega_{\mathrm{d}}} \cos\theta_a^{\mathrm{X}} \\ N_{a,u}^{\Omega_{\mathrm{d}}} \cos\theta_a^{\mathrm{X}} \\ N_{a,v}^{\Omega_{\mathrm{d}}} \cos\theta_a^{\mathrm{X}} \\ N_a^{\Omega_{\mathrm{d}}} \cos\theta_a^{\mathrm{Y}} \\ N_{a,u}^{\Omega_{\mathrm{d}}} \cos\theta_a^{\mathrm{Y}} \\ N_{a,v}^{\Omega_{\mathrm{d}}} \cos\theta_a^{\mathrm{Y}} \\ N_a^{\Omega_{\mathrm{d}}} \cos\theta_a^{\mathrm{Z}} \\ N_{a,u}^{\Omega_{\mathrm{d}}} \cos\theta_a^{\mathrm{Z}} \\ N_{a,v}^{\Omega_{\mathrm{d}}} \cos\theta_a^{\mathrm{Z}} \end{bmatrix} dudv \quad \text{(S44)}$$

Equation 40 can be expanded as follows.

$$\frac{\partial G_a}{\partial d_b^{\mathrm{dof}}}_{(ab)} = \int \left(\mathcal{F}^{\mathrm{lipid-cstr}}_{,D_m\lambda^{\mathrm{lipid}}}{}_{(m1)} \right)^T \left(\bar{N}_{m(m1)} \right) dudv = \int \begin{bmatrix} \mathcal{F}^{\mathrm{lipid-cstr}}_{,X_{,u}\lambda^{\mathrm{lipid}}} \\ \mathcal{F}^{\mathrm{lipid-cstr}}_{,X_{,v}\lambda^{\mathrm{lipid}}} \\ \mathcal{F}^{\mathrm{lipid-cstr}}_{,Y_{,u}\lambda^{\mathrm{lipid}}} \\ \mathcal{F}^{\mathrm{lipid-cstr}}_{,Y_{,v}\lambda^{\mathrm{lipid}}} \\ \mathcal{F}^{\mathrm{lipid-cstr}}_{,Z_{,u}\lambda^{\mathrm{lipid}}} \\ \mathcal{F}^{\mathrm{lipid-cstr}}_{,Z_{,v}\lambda^{\mathrm{lipid}}} \end{bmatrix}^T \begin{bmatrix} N_{a,u}^{\Omega_{\mathrm{d}}} \cos\theta_a^{\mathrm{X}} \\ N_{a,v}^{\Omega_{\mathrm{d}}} \cos\theta_a^{\mathrm{X}} \\ N_{a,u}^{\Omega_{\mathrm{d}}} \cos\theta_a^{\mathrm{Y}} \\ N_{a,v}^{\Omega_{\mathrm{d}}} \cos\theta_a^{\mathrm{Y}} \\ N_{a,u}^{\Omega_{\mathrm{d}}} \cos\theta_a^{\mathrm{Z}} \\ N_{a,v}^{\Omega_{\mathrm{d}}} \cos\theta_a^{\mathrm{Z}} \end{bmatrix} dudv \quad \text{(S45)}$$

Equation 41 can be expanded as follows.

$$\frac{\partial G_a}{\partial d_b^{\mathrm{dof}}}_{(ab)} = \int \left(\mathcal{F}^{\mathrm{crs-cstr}}_{c,D_m\lambda_c^{\mathrm{crs}}}{}_{(m1)} \right)^T \left(\bar{N}_{m(m1)} \right) dudv = \int \begin{bmatrix} \mathcal{F}^{\mathrm{crs-cstr}}_{c,X_{,u}\lambda_c^{\mathrm{crs}}} \\ \mathcal{F}^{\mathrm{crs-cstr}}_{c,X_{,v}\lambda_c^{\mathrm{crs}}} \\ \mathcal{F}^{\mathrm{crs-cstr}}_{c,Y_{,u}\lambda_c^{\mathrm{crs}}} \\ \mathcal{F}^{\mathrm{crs-cstr}}_{c,Y_{,v}\lambda_c^{\mathrm{crs}}} \\ \mathcal{F}^{\mathrm{crs-cstr}}_{c,Z_{,u}\lambda_c^{\mathrm{crs}}} \\ \mathcal{F}^{\mathrm{crs-cstr}}_{c,Z_{,v}\lambda_c^{\mathrm{crs}}} \end{bmatrix}^T \begin{bmatrix} N_{a,u}^{\Omega_{\mathrm{d}}} \cos\theta_a^{\mathrm{X}} \\ N_{a,v}^{\Omega_{\mathrm{d}}} \cos\theta_a^{\mathrm{X}} \\ N_{a,u}^{\Omega_{\mathrm{d}}} \cos\theta_a^{\mathrm{Y}} \\ N_{a,v}^{\Omega_{\mathrm{d}}} \cos\theta_a^{\mathrm{Y}} \\ N_{a,u}^{\Omega_{\mathrm{d}}} \cos\theta_a^{\mathrm{Z}} \\ N_{a,v}^{\Omega_{\mathrm{d}}} \cos\theta_a^{\mathrm{Z}} \end{bmatrix} dudv \quad \text{(S46)}$$

Equation 42 can be expanded as follows.

$$\frac{\partial G_a}{\partial d_b^{\mathrm{dof}}}_{(ab)} = \int \left[\left(\mathcal{F}_{,\alpha D_n}^{\mathrm{membr-st-cstr}}{}_{(1n)} \right) \left(D_{n,d_b^{\Omega_\mathrm{d}}}{}_{(n1)} \right) \right] N_{a-n_{\Omega_\mathrm{d}}}^{\Omega_\mathrm{s}} dudv =$$

$$\int \left[\begin{bmatrix} \mathcal{F}_{,\alpha X_{,u}}^{\mathrm{membr-st-cstr}} \\ \mathcal{F}_{,\alpha X_{,v}}^{\mathrm{membr-st-cstr}} \\ \mathcal{F}_{,\alpha Y_{,u}}^{\mathrm{membr-st-cstr}} \\ \mathcal{F}_{,\alpha Y_{,v}}^{\mathrm{membr-st-cstr}} \\ \mathcal{F}_{,\alpha Z_{,u}}^{\mathrm{membr-st-cstr}} \\ \mathcal{F}_{,\alpha Z_{,v}}^{\mathrm{membr-st-cstr}} \end{bmatrix}^T \begin{bmatrix} X_{,ud_b^{\Omega_\mathrm{d}}} \\ X_{,vd_b^{\Omega_\mathrm{d}}} \\ Y_{,ud_b^{\Omega_\mathrm{d}}} \\ Y_{,vd_b^{\Omega_\mathrm{d}}} \\ Z_{,ud_b^{\Omega_\mathrm{d}}} \\ Z_{,vd_b^{\Omega_\mathrm{d}}} \end{bmatrix} \right] N_{a-n_{\Omega_\mathrm{d}}}^{\Omega_\mathrm{s}} dudv \quad \text{(S47)}$$

Equation 45 can be expanded as follows.

$$\frac{\partial G_a}{\partial d_b^{\mathrm{dof}}}_{(ab)} = \int \left[\left(\mathcal{F}_{c,K_{1c}D_n}^{\mathrm{crs-all}}{}_{(1n)} \right) \left(D_{n,d_b^{\Omega_\mathrm{d}}}{}_{(n1)} \right) \right] N_{a-n_{\Omega_\mathrm{d}}-n_{\Omega_\mathrm{s}}-\sum_{\bar{c}=1}^{c-1} n_{\Omega_{\mathrm{k}_{1\bar{c}}}}}^{\Omega_{\mathrm{k}_{1c}}} dudv =$$

$$\int \left[\begin{bmatrix} \mathcal{F}_{c,K_{1c}X}^{\mathrm{crs-all}} & \cdots & \mathcal{F}_{c,K_{1c}Z_v}^{\mathrm{crs-all}} \end{bmatrix} \begin{bmatrix} X_{,d_b^{\Omega_\mathrm{d}}} \\ X_{,ud_b^{\Omega_\mathrm{d}}} \\ X_{,vd_b^{\Omega_\mathrm{d}}} \\ Y_{,d_b^{\Omega_\mathrm{d}}} \\ Y_{,ud_b^{\Omega_\mathrm{d}}} \\ Y_{,vd_b^{\Omega_\mathrm{d}}} \\ Z_{,d_b^{\Omega_\mathrm{d}}} \\ Z_{,ud_b^{\Omega_\mathrm{d}}} \\ Z_{,vd_b^{\Omega_\mathrm{d}}} \end{bmatrix} \right] N_{a-n_{\Omega_\mathrm{d}}-n_{\Omega_\mathrm{s}}-\sum_{\bar{c}=1}^{c-1} n_{\Omega_{\mathrm{k}_{1\bar{c}}}}}^{\Omega_{\mathrm{k}_{1c}}} dudv \quad \text{(S48)}$$

Equation 48 can be expanded as follows.

$$\frac{\partial G_a}{\partial d_b^{\mathrm{dof}}}_{(ab)} = \int \left[\left(\mathcal{F}_{c,K_{2c}D_n}^{\mathrm{crs-all}}{}_{(1n)} \right) \left(D_{n,d_b^{\Omega_\mathrm{d}}}{}_{(n1)} \right) \right] N_{a-n_{\Omega_\mathrm{d}}-n_{\Omega_\mathrm{s}}-\sum_{\bar{c}=1}^{n_{\mathrm{crs}}} n_{\Omega_{\mathrm{k}_{1\bar{c}}}}-\sum_{\bar{c}=1}^{c-1} n_{\Omega_{\mathrm{k}_{2\bar{c}}}}}^{\Omega_{\mathrm{k}_{2c}}} dudv =$$

$$\int \left[\begin{bmatrix} \mathcal{F}^{\mathrm{crs-all}}_{c,K_{2c}X} & \cdots & \mathcal{F}^{\mathrm{crs-all}}_{c,K_{2c}Z_v} \end{bmatrix} \begin{bmatrix} X_{,d_b^{\Omega_\mathrm{d}}} \\ X_{,ud_b^{\Omega_\mathrm{d}}} \\ X_{,vd_b^{\Omega_\mathrm{d}}} \\ Y_{,d_b^{\Omega_\mathrm{d}}} \\ Y_{,ud_b^{\Omega_\mathrm{d}}} \\ Y_{,vd_b^{\Omega_\mathrm{d}}} \\ Z_{,d_b^{\Omega_\mathrm{d}}} \\ Z_{,ud_b^{\Omega_\mathrm{d}}} \\ Z_{,vd_b^{\Omega_\mathrm{d}}} \end{bmatrix} \right] N^{\Omega_{\mathrm{k}_{2c}}}_{a-n_{\Omega_\mathrm{d}}-n_{\Omega_\mathrm{s}}-\sum_{\bar{c}=1}^{n_\mathrm{crs}} n_{\Omega_{\mathrm{k}_{1\bar{c}}}}-\sum_{\bar{c}=1}^{c-1} n_{\Omega_{\mathrm{k}_{2\bar{c}}}}} dudv \quad \text{(S49)}$$

Equation 51 can be expanded as follows.

$$\frac{\partial G_a}{\partial d_b^{\mathrm{dof}}}_{(ab)} = \left(\mathcal{F}^{\mathrm{lipid-cstr}}_{,\lambda^{\mathrm{lipid}} D_n}{}_{(1n)} \right) \left(D_{n,d_b^{\Omega_\mathrm{d}}}{}_{(n1)} \right) dudv = \int \begin{bmatrix} \mathcal{F}^{\mathrm{lipid-cstr}}_{,\lambda^{\mathrm{lipid}} X_{,u}} & \cdots & \mathcal{F}^{\mathrm{lipid-cstr}}_{,\lambda^{\mathrm{lipid}} Z_{,v}} \end{bmatrix} \begin{bmatrix} X_{,ud_b^{\Omega_\mathrm{d}}} \\ X_{,vd_b^{\Omega_\mathrm{d}}} \\ Y_{,ud_b^{\Omega_\mathrm{d}}} \\ Y_{,vd_b^{\Omega_\mathrm{d}}} \\ Z_{,ud_b^{\Omega_\mathrm{d}}} \\ Z_{,vd_b^{\Omega_\mathrm{d}}} \end{bmatrix} dudv$$

(S50)

Equation 53 can be expanded as follows.

$$\frac{\partial G_a}{\partial d_b^{\mathrm{dof}}}_{(ab)} = \left(\mathcal{F}^{\mathrm{crs-cstr}}_{c,\lambda_c^{\mathrm{crs}} D_n}{}_{(1n)} \right) \left(D_{n,d_b^{\Omega_\mathrm{d}}}{}_{(n1)} \right) dudv = \int \begin{bmatrix} \mathcal{F}^{\mathrm{crs-cstr}}_{c,\lambda_c^{\mathrm{crs}} X_{,u}} & \cdots & \mathcal{F}^{\mathrm{crs-cstr}}_{c,\lambda_c^{\mathrm{crs}} Z_{,v}} \end{bmatrix} \begin{bmatrix} X_{,ud_b^{\Omega_\mathrm{d}}} \\ X_{,vd_b^{\Omega_\mathrm{d}}} \\ Y_{,ud_b^{\Omega_\mathrm{d}}} \\ Y_{,vd_b^{\Omega_\mathrm{d}}} \\ Z_{,ud_b^{\Omega_\mathrm{d}}} \\ Z_{,vd_b^{\Omega_\mathrm{d}}} \end{bmatrix} dudv$$

(S51)

Supplementary Notes

Lipid sorting simulation

According to previous works, a variety of different lipid species and classes can collectively form cellular membranes. For example, a lipidomic study performed on human islets detected a total of 329 lipid species across eight major lipid classes [51]. A recent study on single-cell lipid profiling identified 81 species from six major lipid classes [52]. Therefore, eight lipids with different bending moduli were considered for the calculations in Fig. 2.

Regularly defined nine crosslinkers in a $1.547 \times 1.547\ \mu m^2$ planar membrane were anti-parallelly stimulated, as shown in Fig. 2A and B. The displacement of elements in the boundary region of the square membrane was also prescribed, as shown in Fig. 2A, by sampling calculated values from the interior region (see Fig. S14 for the boundary region).

To implement lipid sorting in the membrane, the combined membrane energy density value (using mean curvatures and lateral strain values) for all elements was calculated at the end of each calculation performed by prescribing the displacement. The Gaussian curvature energy density was not considered in this process based on the Gauss-Bonnet theorem. With the given energy densities, a lower bending modulus was assigned to the element with a higher energy density value. By counting the cumulative number of lipids assigned to elements, the process was repeated and continued until all elements of the membrane were assigned a bending modulus value. This set of processes was repeated for each step in which the displacement was prescribed.

The described lipid sorting process was identically applied to the calculations in Fig. 4.

Segmentation algorithm to define lipid nanodomains for calculations in Fig. 2.

A segmentation algorithm was implemented to define lipid nanodomains in the finite element model framework after lipid sorting. For a given quadrilateral element in the membrane, four adjacent elements that share an edge with the given center element were evaluated with respect to whether their bending moduli are the same as the bending modulus of the center element. This process was repeated with the growth of a nanodomain and completed when no adjacent element was found with the same bending modulus. Here, elements with a shared vertex (without a shared edge) were not included in the same lipid nanodomain. Note that the nanodomains identified with the most outer boundary elements of the square membrane were not considered in calculating mean areas in Fig. 2O because of the possibility that the nanodomains could continue beyond the boundary. MATLAB codes are available for the segmentation analysis.

The half max M_c area and the confined integrin diffusion area in the reference[38]

To estimate the area for the confined Brownian motion of crosslinkers, the area defined by half of the maximum M_c was calculated. The physical domain of the membrane made by $(n_{\text{grid\_u}} - 2) \times (n_{\text{grid\_v}} - 2)$ elements (regions except the most outer boundary elements in Fig. S1A) was considered, where $n_{\text{grid\_u}}$ and $n_{\text{grid\_v}}$ are the number of regularly defined grid regions on the parametric u and v coordinates, respectively. Then, M_c values at $4(n_{\text{grid\_u}} - 2)(n_{\text{grid\_v}} - 2) + 2(n_{\text{grid\_u}} - 2) + 2(n_{\text{grid\_v}} - 2) + 1$ regularly defined points in the membrane were tested with respect to whether they are equal or greater than the half max M_c value. From the sampled points, the area was calculated by using the alphaShape (with the default alpha radius) and surfaceArea functions in MATLAB.

To estimate the confined integrin diffusion area in Fig. 2A in the reference[38], WebPlotDigitizer

(https://automeris.io/, which is also used to extract experimental data in Fig. 5) was used to extract data points. Then, the area was calculated using alphaShape (with the default alpha radius) and area functions in MATLAB.

Remeshing process for the finite element model

For all calculations presented in this article, unless mentioned otherwise, the regulation of mesh grids was conducted for the solution of the previous step, before using that as the initial guess of the given step in the Newton-Raphson method. A simple remeshing algorithm was implemented as follows. First, by using the calculated membrane-crosslinker solution, all membrane arclengths on the parametric mesh grid in the direction of u were uniformly discretized. Second, this process was repeated for the arclengths on the mesh grid in the direction of v. Third, the control point values i.e., the weights of the B-spline function, for the displacement functions *X*, *Y*, and *Z* were adjusted until the corresponding membrane mesh became close enough to the uniformly regulated membrane mesh. This was implemented by comparing the adjusted *X*, *Y*, and *Z* values at the parametric center of the adjusted elements with the center values from the uniformly regulated elements. By doing this, $x_{i_{\mathrm{d}}}^{\mathrm{ref}}$, $y_{i_{\mathrm{d}}}^{\mathrm{ref}}$, and $z_{i_{\mathrm{d}}}^{\mathrm{ref}}$ in Equations 16–18 can be determined from the adjusted control point values i.e., the weights. The same process was repeated for α, $K_{1\mathrm{c}}$, and $K_{2\mathrm{c}}$.

For all data in this work, the first three displacement steps were calculated without the remeshing procedure. Additionally, data between the 34th point and the 61st point in Fig. 4F blue trace were obtained without the remeshing process because the Newton-Raphson method did not provide a converged solution, perhaps due to poor initial guesses after the remeshing process. Also, the remeshing was omitted as it was not relevant for all calculations in Fig. S2.

Calculations for the lipid number strain α around membrane-inserted proteins.

A set of calculations was performed to test whether non-negligible gradients of lipid number strain α can be formed around membrane-inserted proteins. The strain α was calculated for a rectangular membrane where eight membrane proteins are considered, as shown in Fig. S2. To define the protein insertion points, two-by-two elements were selected to generate the zero-strain condition at their shared vertex (see Fig. S2C). To increase the strain in the rectangular region, the total number of lipids (n_{lipid} in Equation 1) was reduced. The strain values for the boundary of the rectangular membrane were also prescribed (see Fig. S2C).

Two different inter-protein distances were tested. The distance was about 0.9 nm and 0.9 Å in Fig. S2A and B, respectively. The number of elements was the same for both cases. A steeper gradient was generated with the reduced inter-protein distance. According to data in Fig. S2B, the length scale of the gradient can be smaller than 1 Å. The results in Fig. S2 allowed us to assume that the strain gradient around the membrane-inserted protein can be negligible.

Discussion for the kinetic energy of the inclusion-crosslinker complex under confined diffusion.

As a quasi-static problem, the functional used in applying the principle of stationary total potential energy for the membrane-crosslinker complex can be mainly defined with the elastic energy as in Equation 1 (without the kinetic energy term). In the stationary configuration, there might still be a kinetic energy contribution due to the confined diffusion motion of the inclusion-crosslinker complex. However, its magnitude might be negligible. For example, the confined diffusion kinetic energy of a single integrin with a talin monomer can be approximated as follows.

$$\frac{1}{2}m_{\text{itc}}{v_{\text{eff}}}^2 \leq 2m_{\text{itc}}D_{\text{itc}}/t = 2.67\times10^{-29}\text{ J} \quad \text{(S52)}$$

where the effective velocity of the integrin-talin complex i.e., $v_{\text{eff}}=\sqrt{4D_{\text{itc}}/t}$, can be derived from

its root mean square displacement i.e., $\mathrm{RMSD} = \sqrt{4D_{\mathrm{itc}}t}$. $m_{\mathrm{itc}} = 8.9 \times 10^{-19}\mathrm{gram}$ is the approximated mass of the complex of an integrin heterodimer (266 kDa[53]) and a talin monomer (270 kDa[54]); $D_{\mathrm{itc}} = 0.3 \times 10^{-12}\,\mathrm{m}^2/s$ is the observed confined diffusion constant of the integrin[37] expected to interact with talin[38]; and $t = 20\,\mathrm{msec}$ is the measurement time step for the diffusion constant[37]. The kinetic energy value in Equation S52 is far smaller than the elastic potential energy $(2.16 \times 10^{-17} - 8.07 \times 10^{-16}$ J) for the crosslinker with $c = 1$ in Fig. 3.

Supplementary Figures

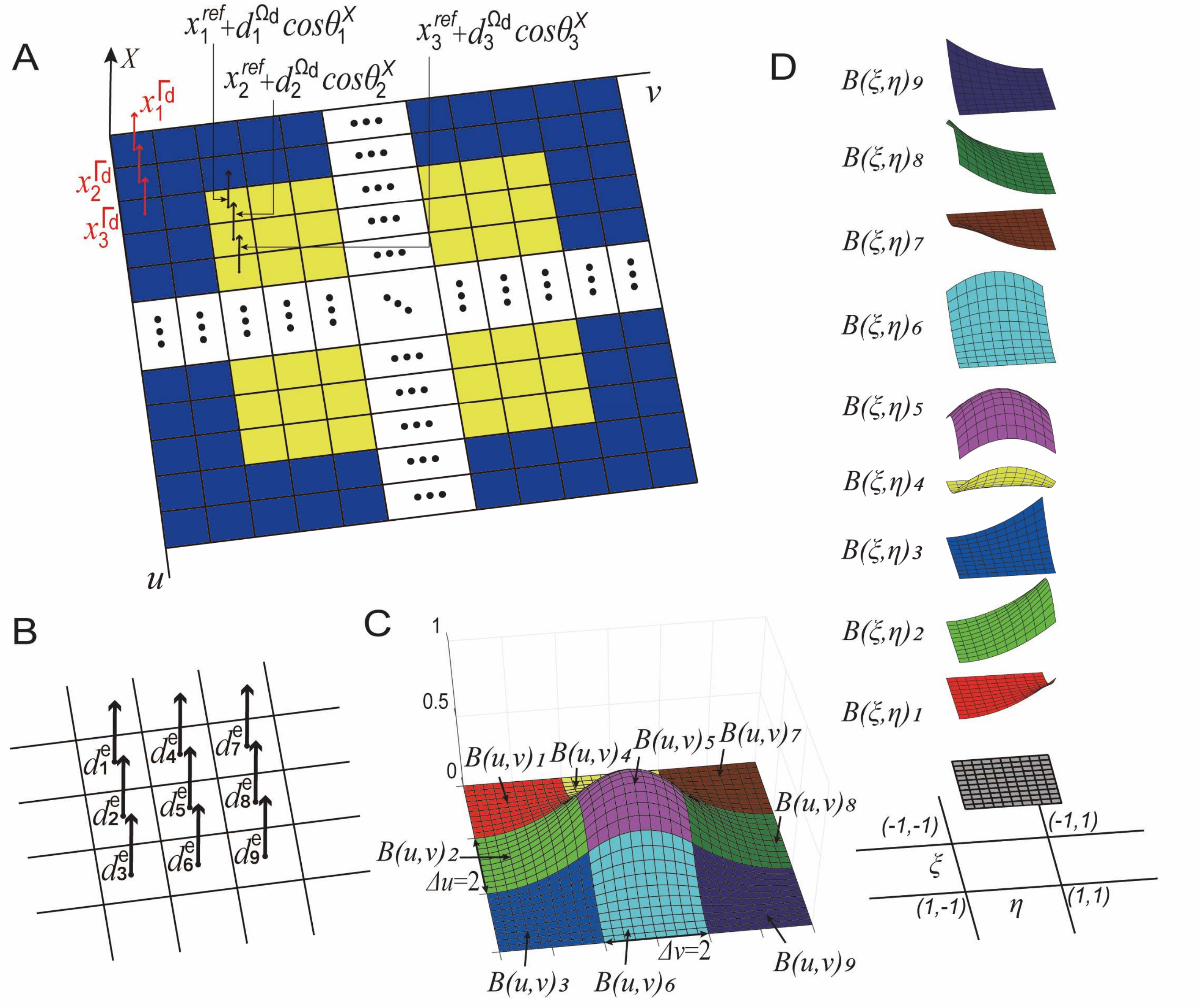

Fig. S1. Description of the finite element model. (**A**) Parametric coordinates u and v defined for the displacement function X. See Equation 16 for how weights shown with black and red arrows are applied to the B-spline shape functions. The domain for the fixed boundary values (Γ_d) is shown in navy blue. The domain for the unknown variables (Ω_d) is shown in yellow. The displacement functions Y and Z; the function for the lateral strain α; and the functions K_{1c} and K_{2c} can be defined similarly. (**B**) Nine unknowns for X, Y, and Z of an element are shown from the element point of view. The B-spline functions used in this work require nine control point weights to define the local function values for X, Y, Z, α, K_{1c}, and K_{2c}. (**C**) The B-spline basis functions used in this model. (**D**) The nine basis functions in the parent domain defined by $\xi \in [-1,1]$ and $\eta \in [-1,1]$ (See Equations S31–S39).

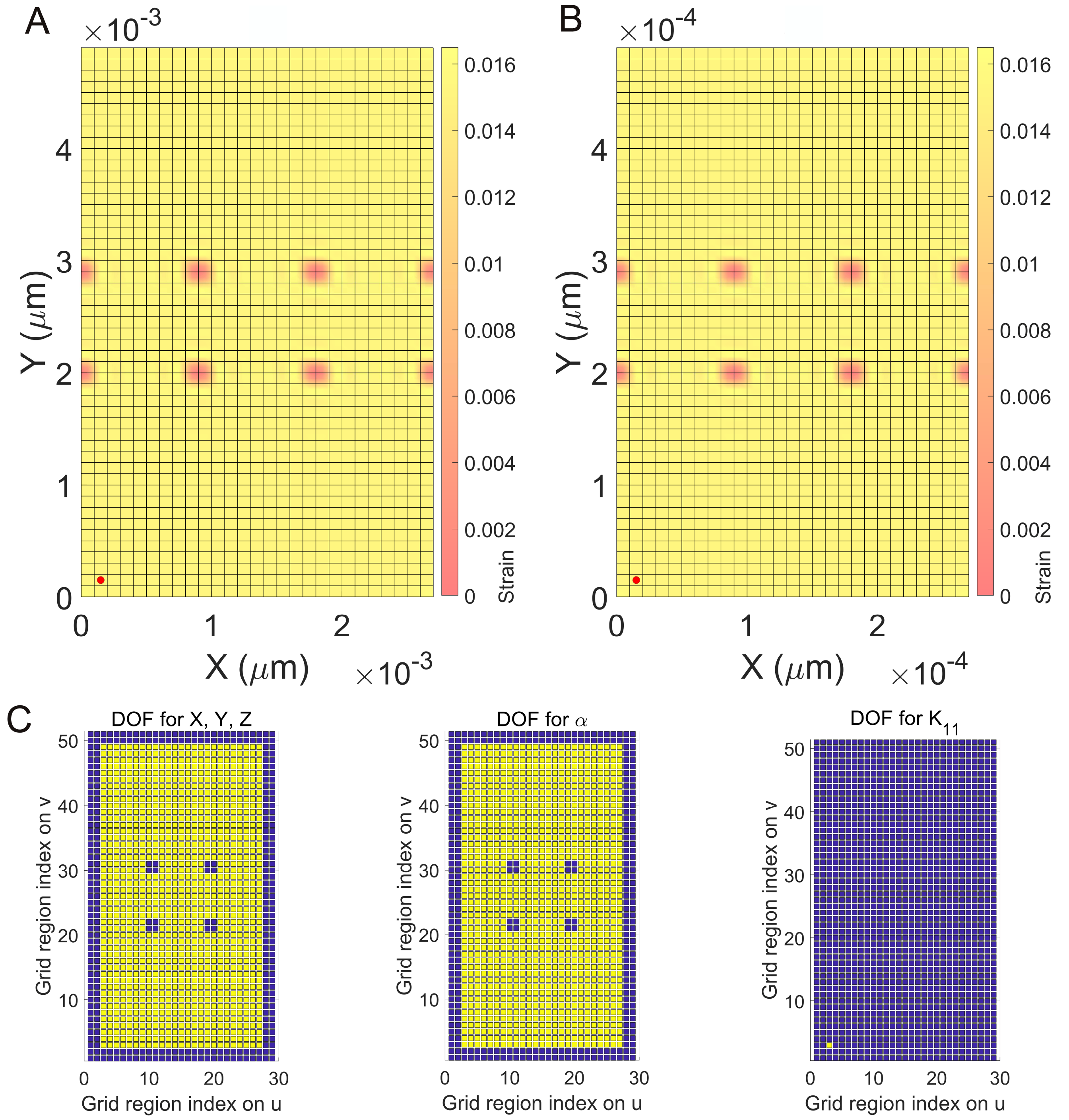

Fig. S2. The profile of the lipid number strain around membrane-inserted proteins. Eight points (vertices) were assumed for the membrane-inserted proteins where the lateral strain α is zero. Calculations were performed by reducing the n_{lipid} value. (**A**) Calculation for the lateral strain profile when the protein-protein distance is about 0.9 nm. (**B**) Another calculation for the strain profile when the protein-protein distance is about 0.9 Å. The number of total elements is the same for (A) and (B). A crosslinker with one element for degrees of freedom (DOFs) and a negligible φ value ($\varphi = 2 \times 10^{-25}\sqrt{\text{N}}$) was considered at the bottom left corner due to a programming issue. (**C**) DOFs for each element. Yellow: regions with unknowns. Navy blue: regions with fixed boundary values. The plot for K_{21} is identical to the plot for K_{11} in (C, right).

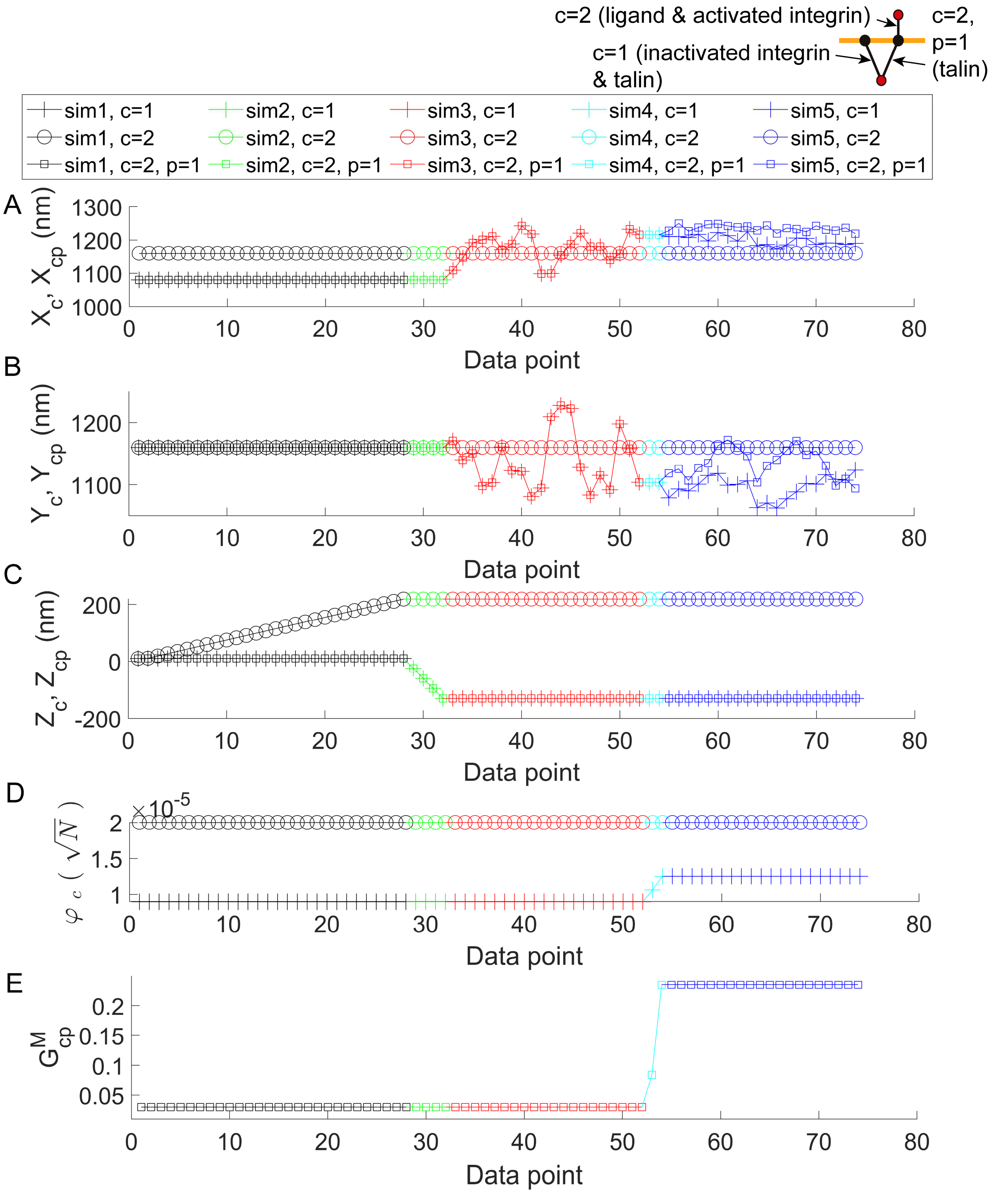

Fig. S3. Inputs for calculations in Fig. 3. (**A–E**) Inputs for a set of five simulations shown in Fig. 3. Different simulations are plotted using different colors.

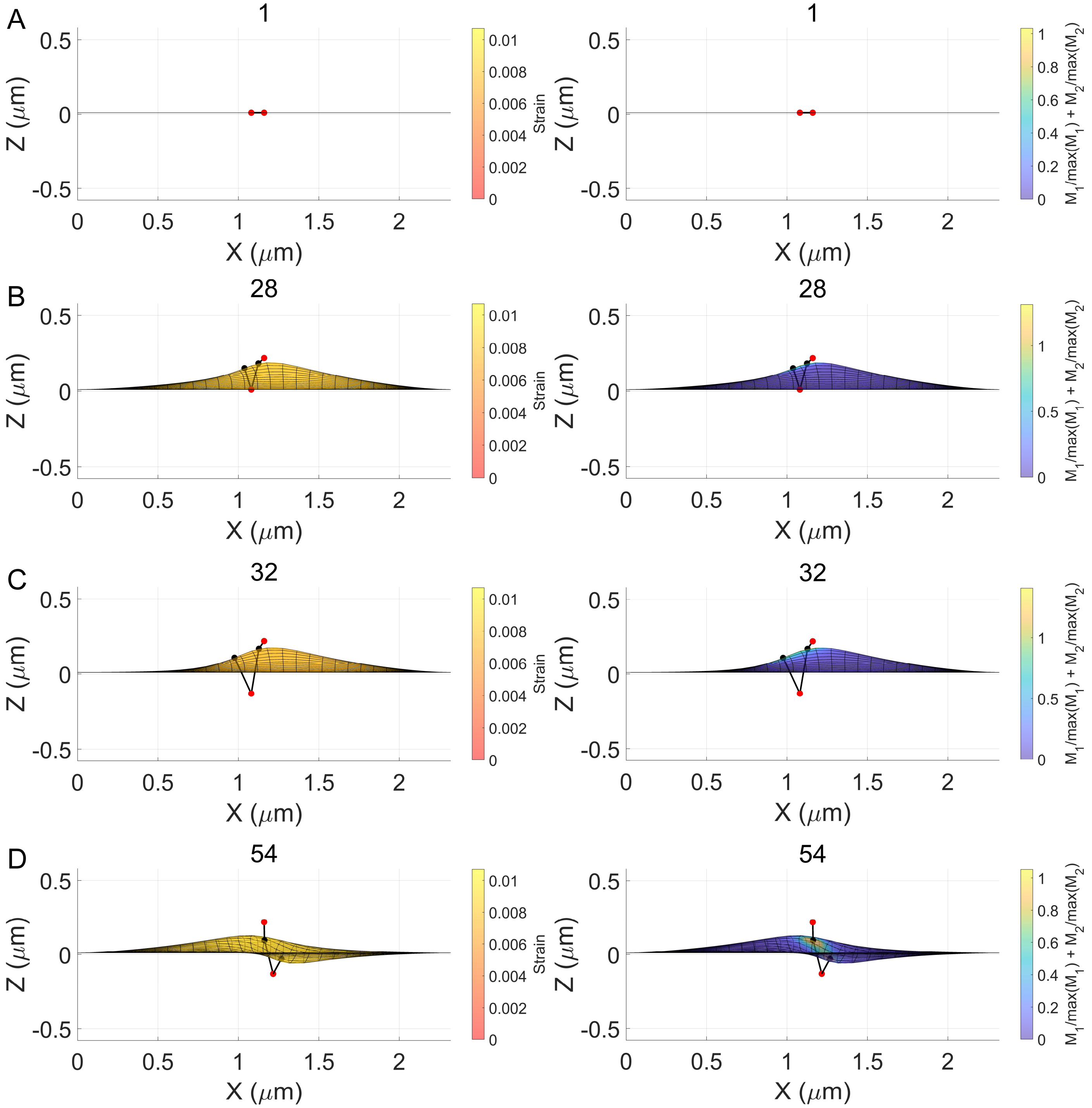

Fig. S4. Side view of the membrane-crosslinker complex in Fig. 3. The number above each plot represents the corresponding data point index.

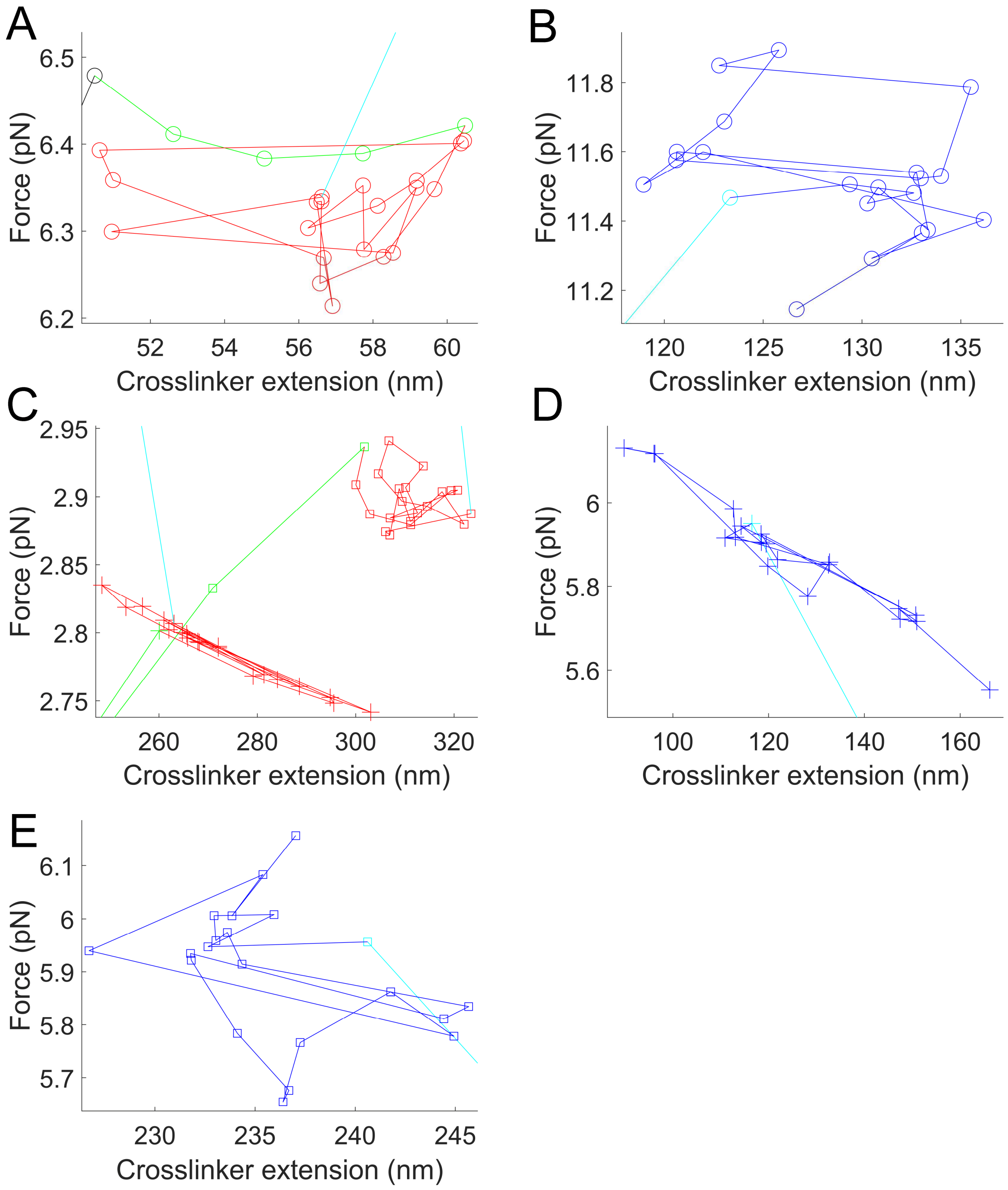

Fig. S5. Expanded view of the regions marked in Fig. 3A.

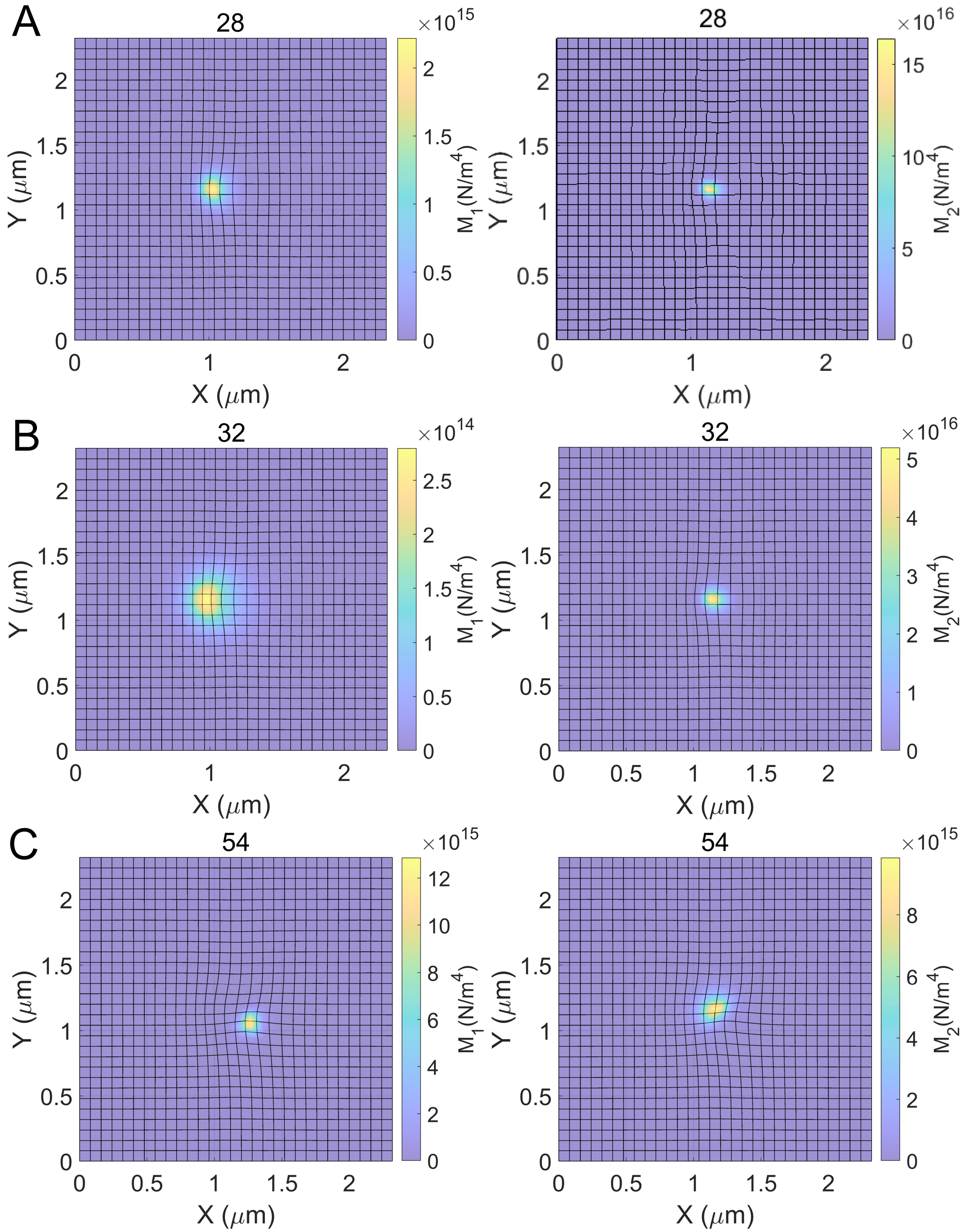

Fig. S6. Top view of the membrane-crosslinker complex in Fig. 3 with the surface map of the elastic modulus density. The number on top of each plot indicates the index for data in Fig. 3. The elastic modulus density for $(c, p) = (2,1)$ can be simply written as $G_{21}^{\mathrm{M}} M_2$.

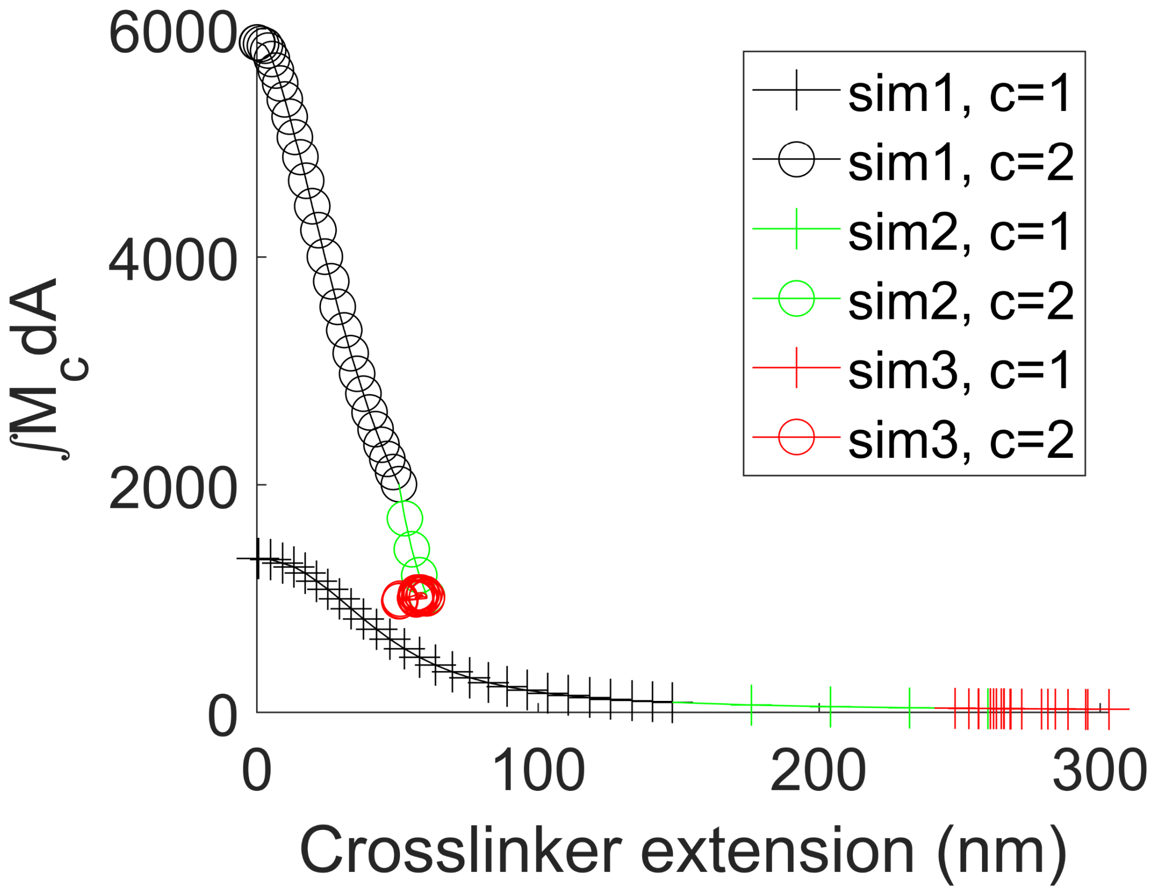

Fig. S7. Total elastic modulus density $\int M_c dA$ for sim1–3 in Fig. 3. Here, $\varphi_1 = 9 \times 10^{-6}\sqrt{\mathrm{N}}$, $\varphi_2 = 2 \times 10^{-5}\sqrt{\mathrm{N}}$, and $G_{21}^{\mathrm{M}} = 0.03$.

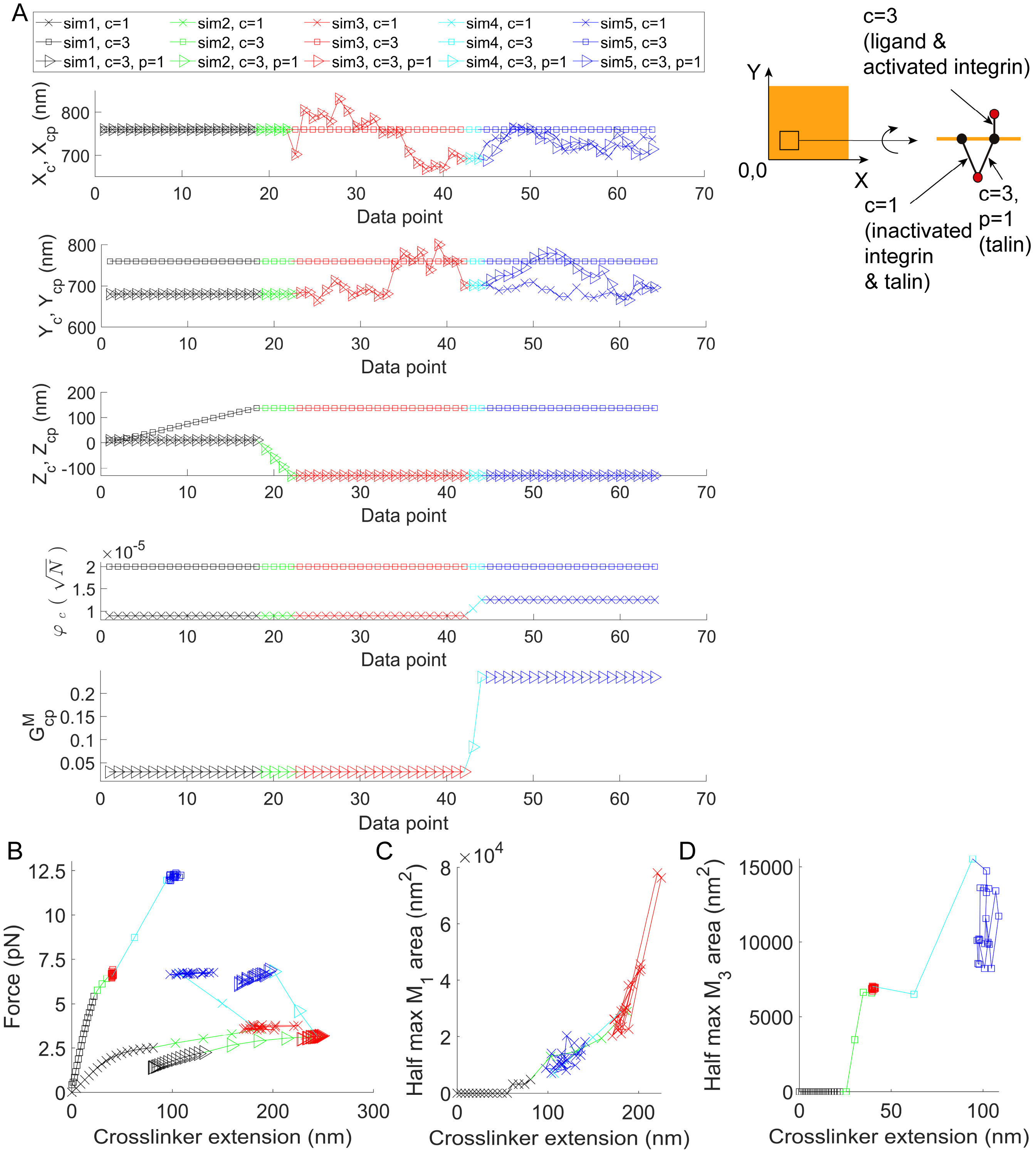

Fig. S8. Responses of crosslinkers considering four integrin-adhesion sites. See Movie S3 for all membrane-crosslinker configurations. The initial size of the membrane, as well as parameter values for the membrane and crosslinkers, are the same as those used in Fig. 3. Responses of crosslinkers at the bottom left corner of the membrane are presented (see inset and Movie S3). (**A–D**) Inputs (A) and responses (B–D) of crosslinkers with $c = 1$ (a complex of an inactivated integrin and a talin monomer), $c = 3$ (integrin-ligand complex), and $(c, p) = (3,1)$ (talin). See Fig. S10 for DOFs.

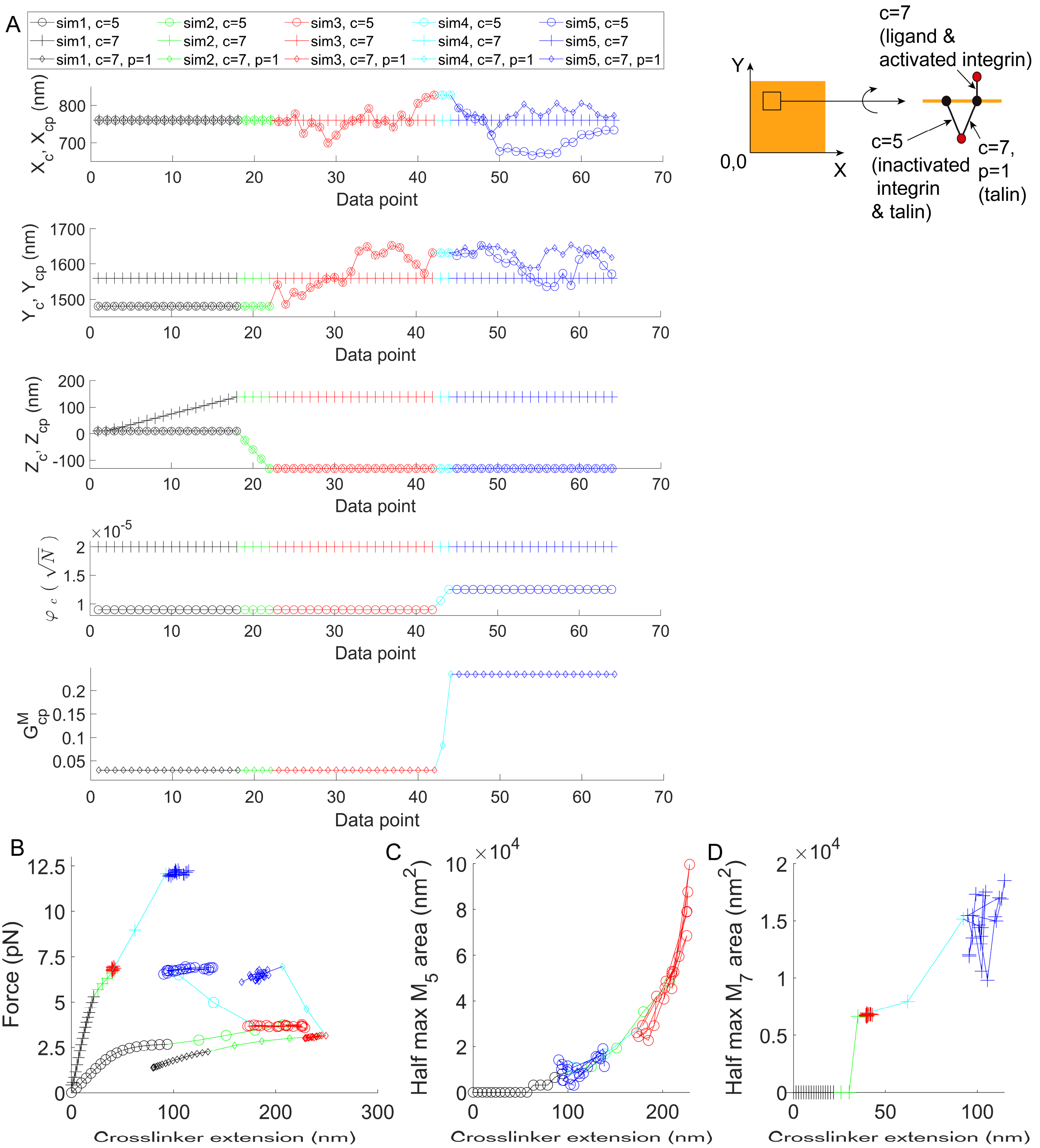

Fig. S9. Responses of other crosslinkers in the membrane-crosslinker complex for Fig. S8 and Movie S3. As a continuation of the analyses in Fig. S8, responses of crosslinkers at the top-left corner of the membrane are presented here (see inset and Movie S3). (**A–D**) Inputs (A) and responses (B–D) of crosslinkers with $c = 5$ (a complex of an inactivated integrin and a talin monomer), $c = 7$ (an integrin-ligand complex), and $(c, p) = (7,1)$ (talin).

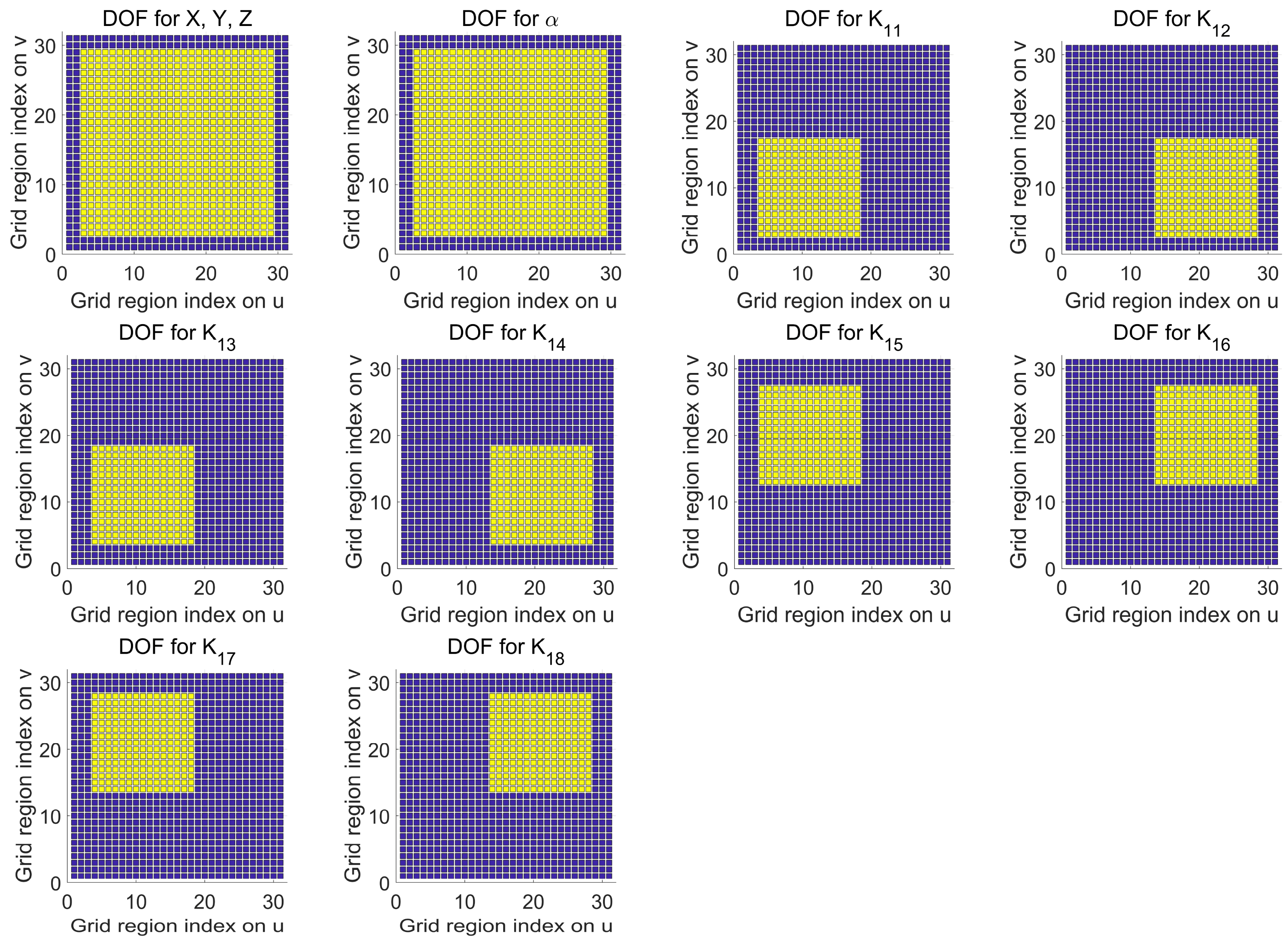

Fig. S10. Degrees of freedom (DOFs) for the membrane-crosslinker complex in Figs. S8 and S9. Yellow: regions with unknowns. Navy blue: regions with fixed boundary values. The plot for K_{2c} is identical to the plot for K_{1c}.

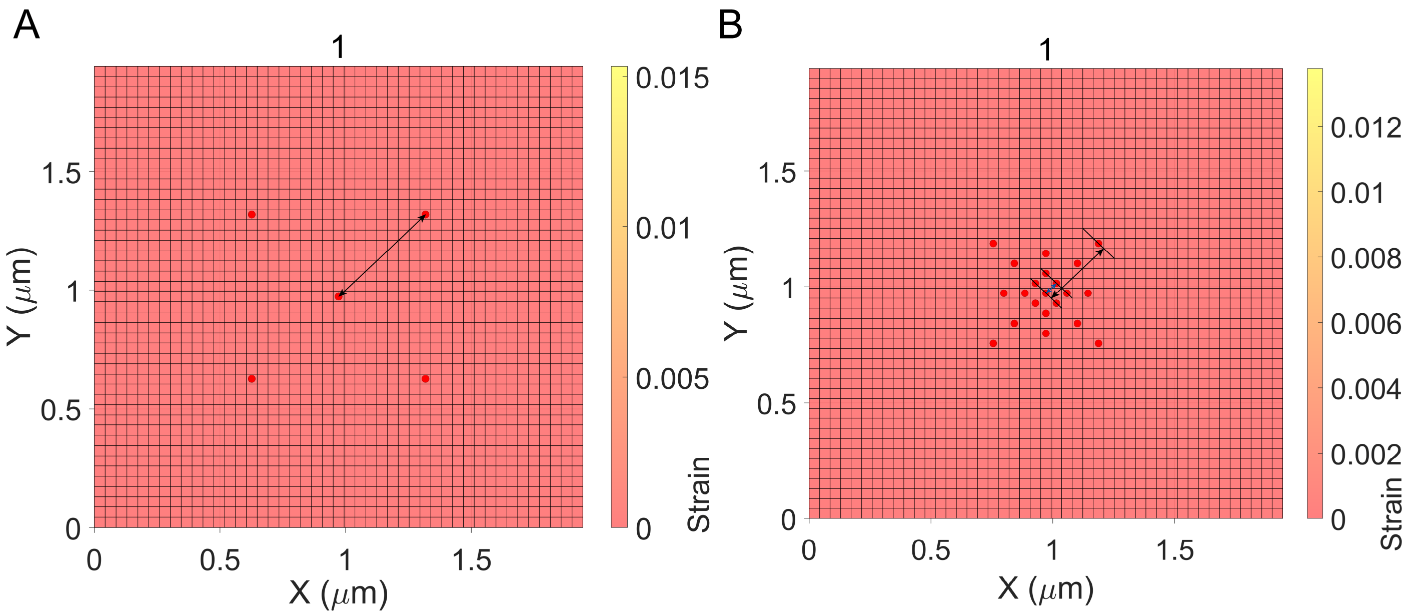

Fig. S11. Initial membrane-crosslinker configurations for Fig. 4. (**A, B**) Top view of the initial configuration for membrane-crosslinker complexes in Fig. 4. (A) and (B) correspond to Fig. 4A and Fig. 4D, respectively. The distances indicated with arrows are 488.8 nm for (A); 61.1 nm for (B blue); and 305.5 nm for (B black).

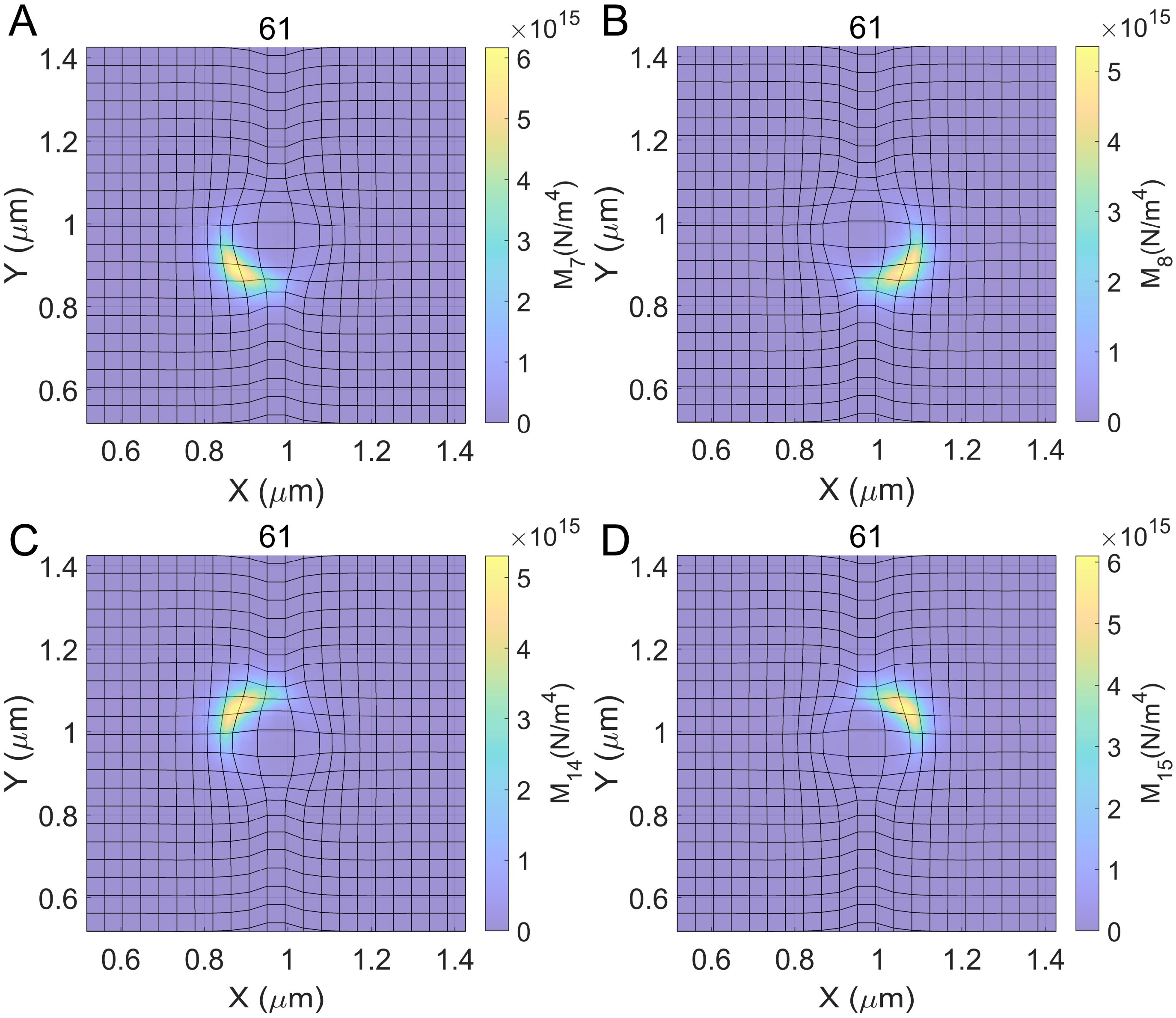

Fig. S12. Expanded top view of the surface elastic modulus density for the membrane-crosslinker complex in Fig. 4D. The density profiles for crosslinkers with $c = 7, 8, 14,$ and 15 are plotted.

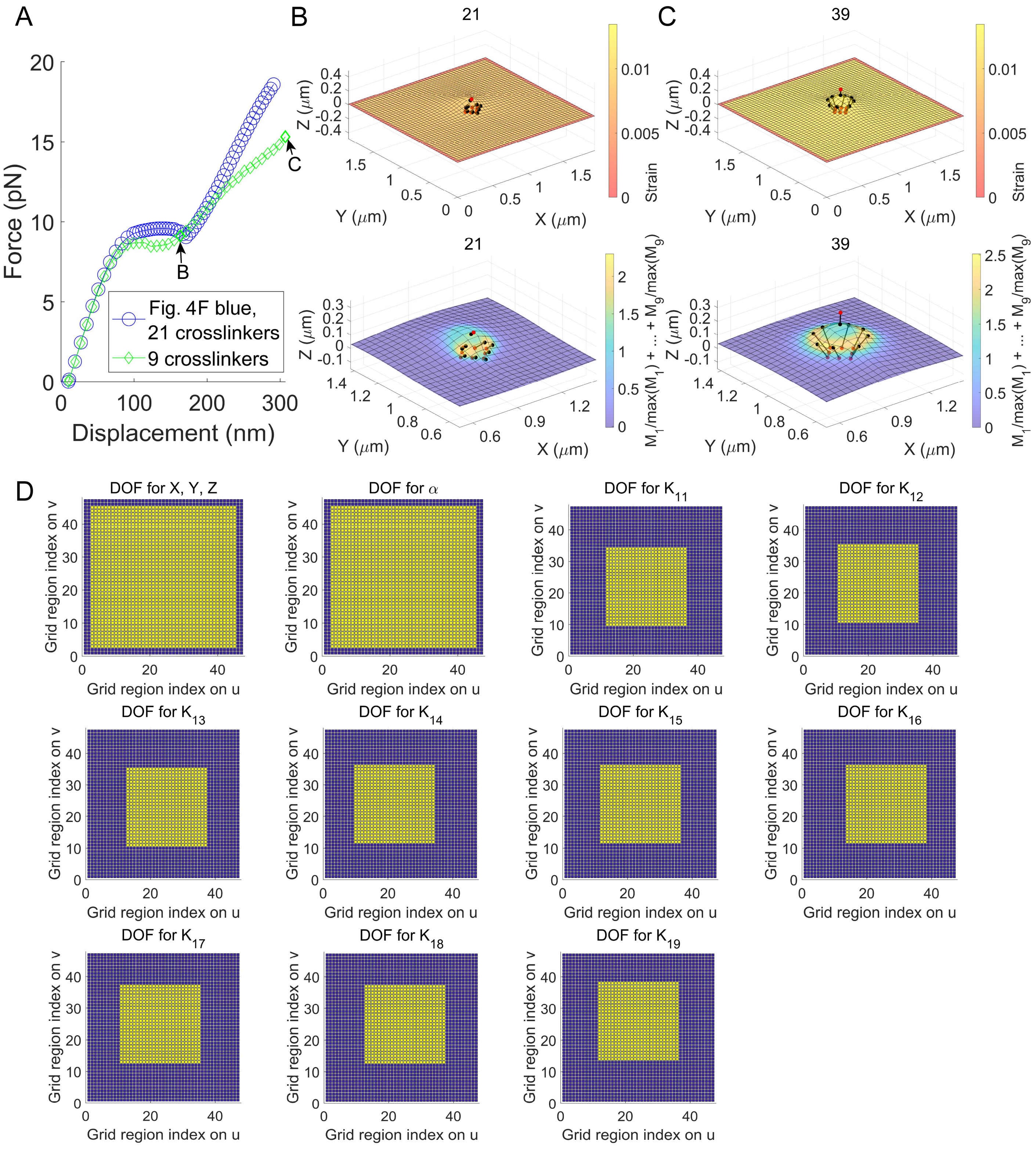

Fig. S13. Demonstration of broken deformation symmetry. The symmetry for the membrane deformation can be broken when the total number of membrane-cytoskeleton crosslinkers clustered within a small area is insufficient. (**A**) Comparison between force vs. displacement responses with twenty-one (Fig. 4F blue) and nine crosslinkers. The crosslinker located at the center of the membrane is displaced by varying $\bar{Z}_c$. (**B, C**) Calculated membrane-crosslinker configurations for the green diamonds in (A). See Movie S8 for all calculated membrane-crosslinker configurations. (**D**) Degrees of freedom (DOFs) for data shown with green

diamonds in (A). Yellow: regions with unknowns. Navy blue: regions with fixed boundary values. The plot for K_{2c} is identical to the plot for K_{1c}.

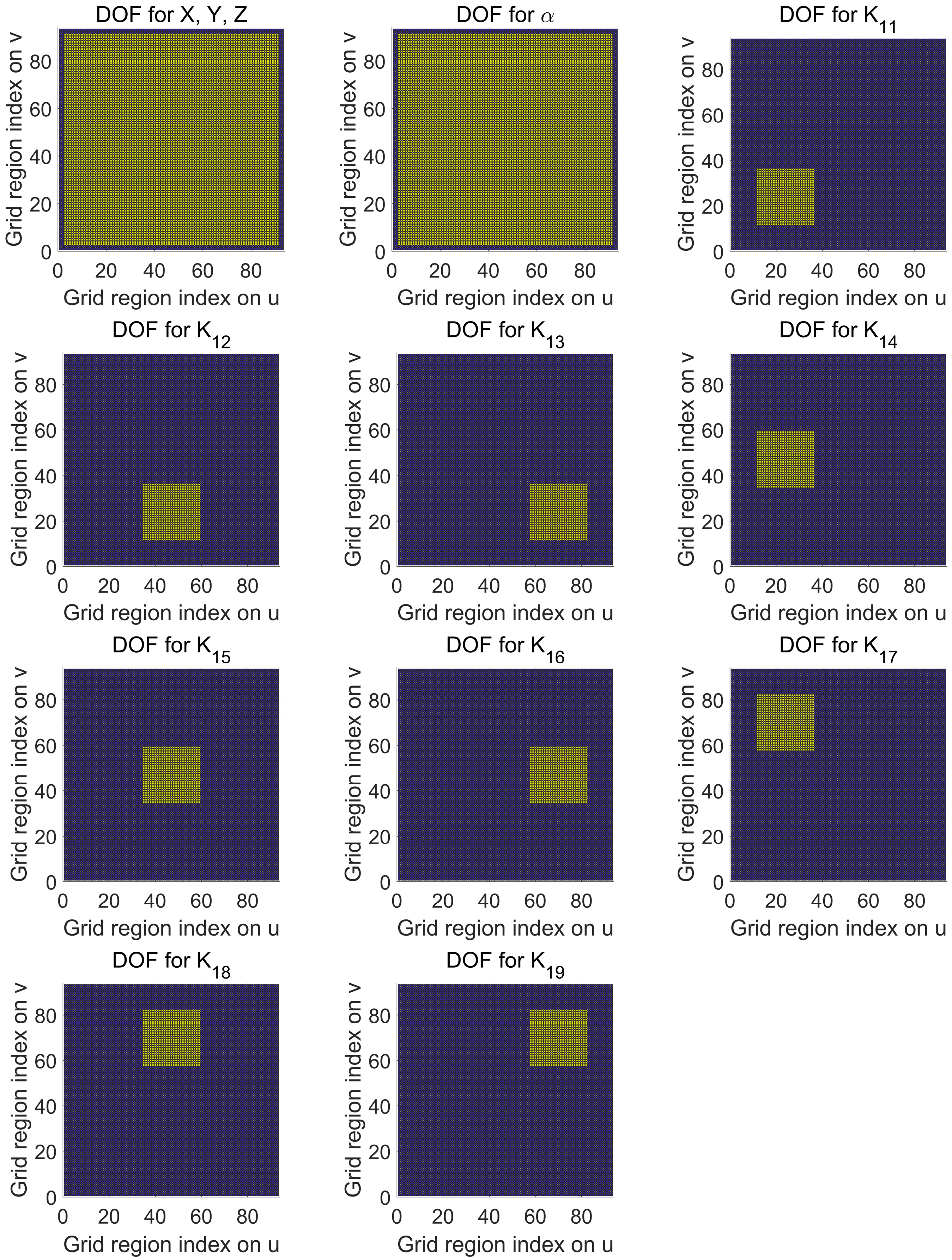

Fig. S14. Degrees of freedom (DOFs) for Fig. 2. Yellow: regions with unknowns. Navy blue: regions with fixed boundary values. The plot for K_{2c} is identical to the plot for K_{1c}.

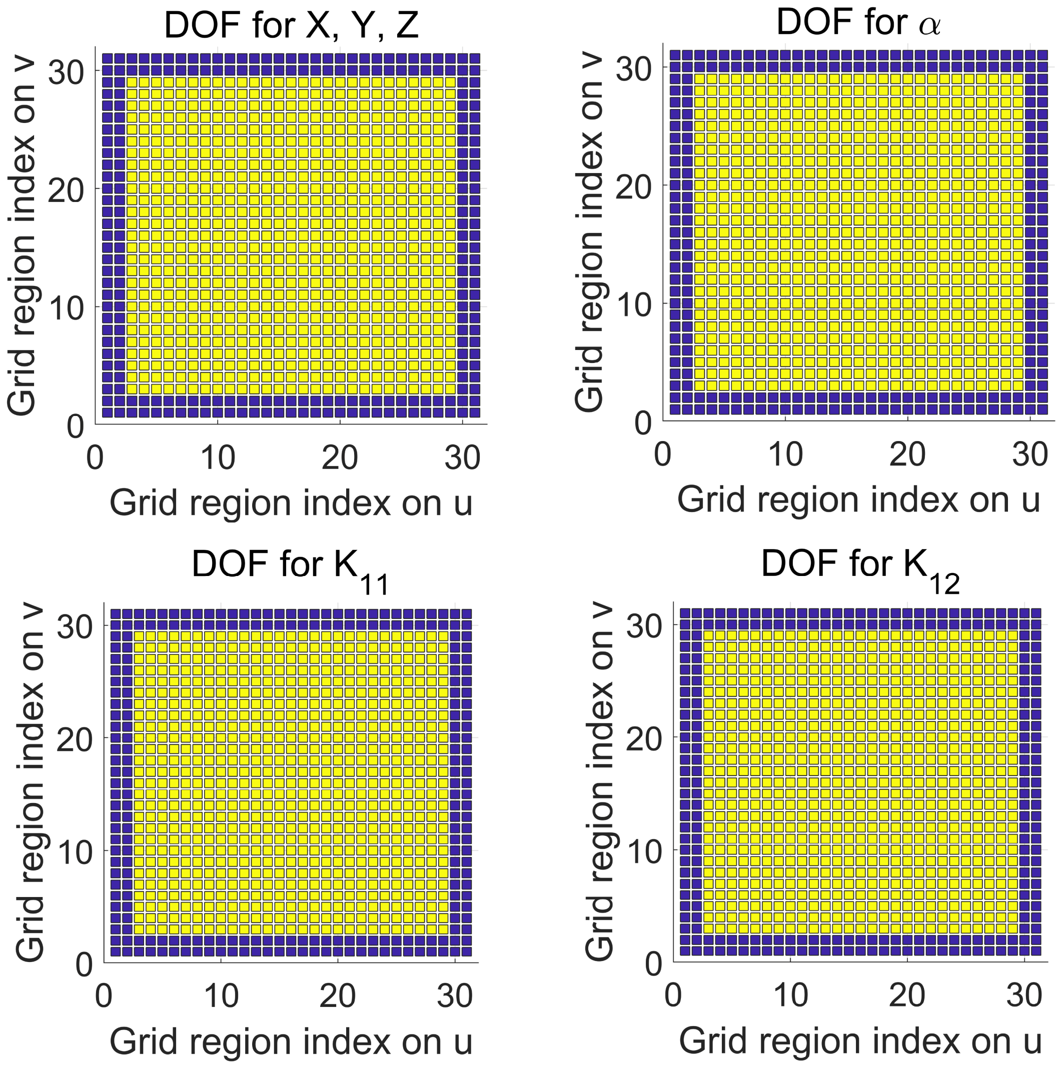

Fig. S15. Degrees of freedom (DOFs) for Fig. 3. Yellow: regions with unknowns. Navy blue: regions with fixed boundary values. The plot for K_{2c} is identical to the plot for K_{1c}.

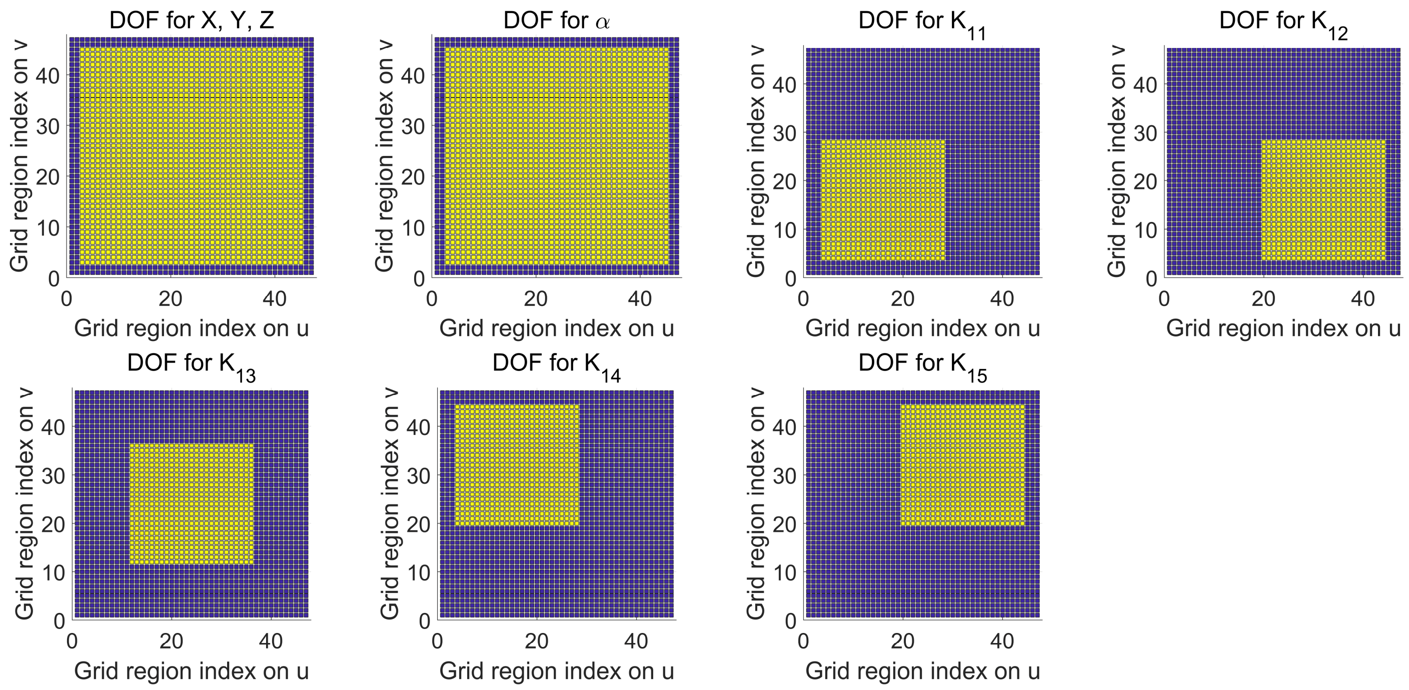

Fig. S16. Degrees of freedom (DOFs) for Fig. 4A–C. Yellow: regions with unknowns. Navy blue: regions with fixed boundary values. The plot for K_{2c} is identical to the plot for K_{1c}.

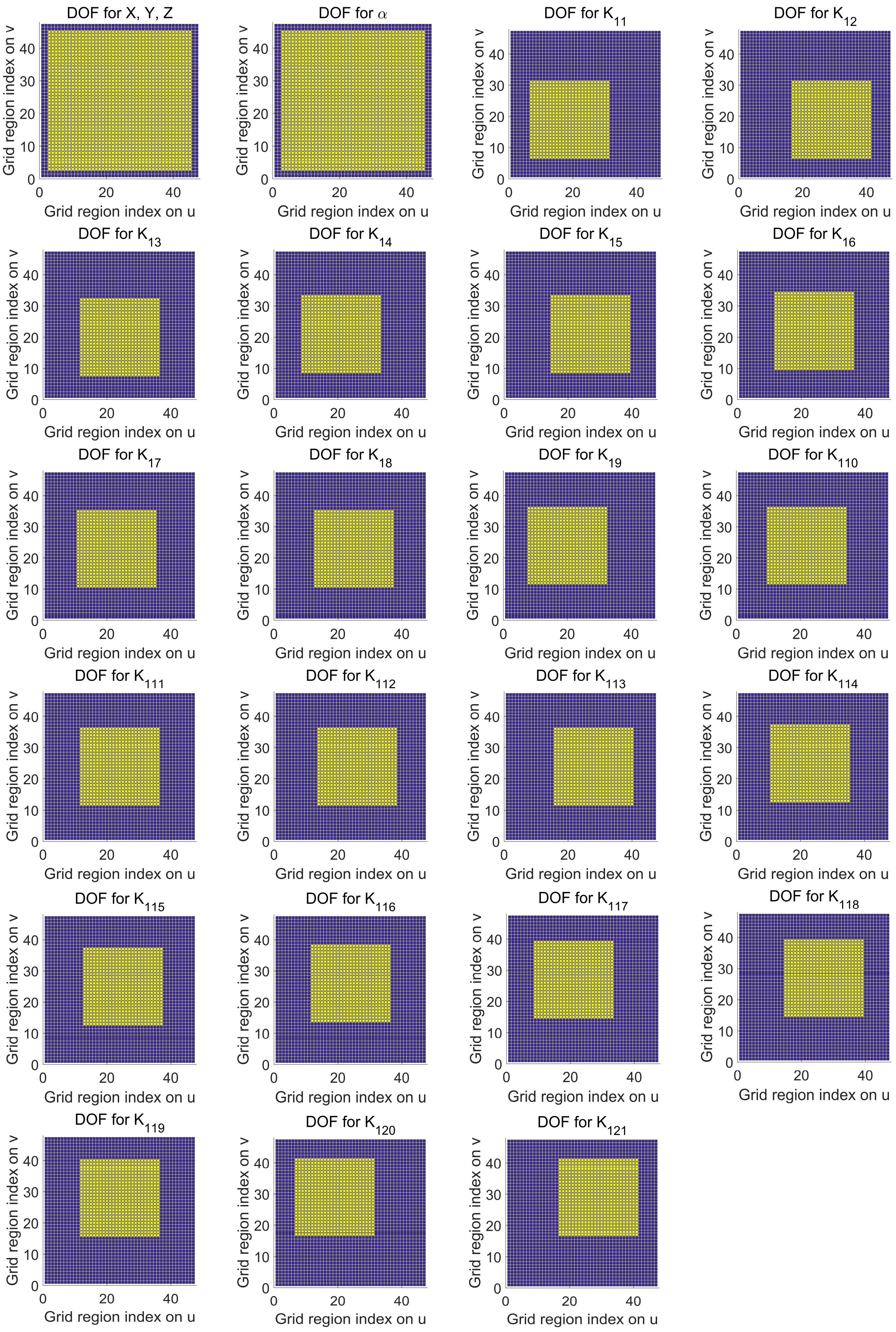
DOF for X, Y, Z
DOF for α
DOF for K_{11}
DOF for K_{12}
DOF for K_{13}
DOF for K_{14}
DOF for K_{15}
DOF for K_{16}
DOF for K_{17}
DOF for K_{18}
DOF for K_{19}
DOF for K_{110}
DOF for K_{111}
DOF for K_{112}
DOF for K_{113}
DOF for K_{114}
DOF for K_{115}
DOF for K_{116}
DOF for K_{117}
DOF for K_{118}
DOF for K_{119}
DOF for K_{120}
DOF for K_{121}
Grid region index on u
Grid region index on v

Fig. S17. Degrees of freedom (DOFs) for Fig. 4D–F. Yellow: regions with unknowns. Navy blue: regions with fixed boundary values. The plot for K_{2c} is identical to the plot for K_{1c}.

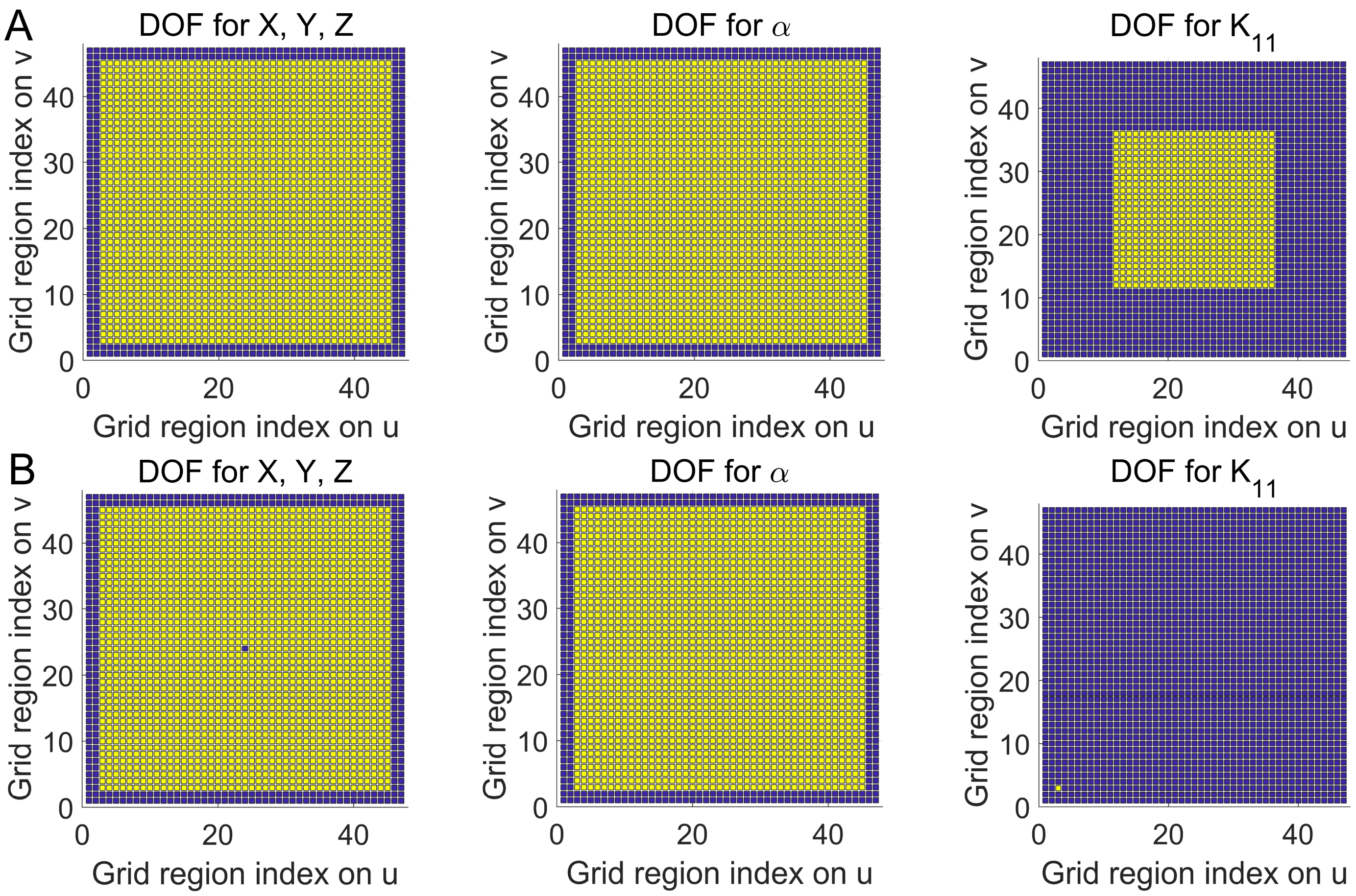

Fig. S18. Degrees of freedom (DOFs) for data in Fig. 6. (**A**) DOFs for the calculations with the center crosslinker (crs force in Fig. 6). (**B**) DOFs for the calculation without the center crosslinker (membrane force in Fig. 6, black empty squares). A crosslinker with one element for DOFs and a negligible φ value ($\varphi = 1 \times 10^{-35}\sqrt{\mathrm{N}}$) was assumed at the bottom left corner due to a programming issue in (B). Yellow: regions with unknowns. Navy blue: regions with fixed boundary values. The plot for K_{2c} is identical to the plot for K_{1c}.

Supplementary Table

**Parameters (description, references, note)**	**Used values (Figure)**
κ (bending modulus of lipid membranes, [50], A)	$10k_\mathrm{b}T$, $15.7k_\mathrm{b}T$, $21.4k_\mathrm{b}T$, $27.1k_\mathrm{b}T$, $32.9k_\mathrm{b}T$, $38.6k_\mathrm{b}T$, $44.3k_\mathrm{b}T$, $50k_\mathrm{b}T$ (Fig. 2); $20k_\mathrm{b}T$ (Figs. 3, 6, S2, S8, S9, and S13); $20k_\mathrm{b}T$ and $50k_\mathrm{b}T$ (Fig. 4)
σ_0 (membrane surface tension at zero strain, [11,40])	$\exp(-10) \times 10^{-3}$ (all)
K_app (apparent area stretching modulus, [50])	$K_\mathrm{app} = \left\{0.1675\left(\frac{\kappa}{k_\mathrm{b}T}\right)^2 - 1.972\left(\frac{\kappa}{k_\mathrm{b}T}\right) + 166.1\right\}10^{-3}$ (all)
ϕ_0 (reference lipid number density at zero strain, [55], B)	$\frac{1000}{629} \times 10^{18}$ (all)

Table S1. Summary of membrane parameters used in this work. (**A**) $k_\mathrm{b} = 1.3806488 \times 10^{-23}$ J/K. $T = 300$ K. (**B**) n_lipid was calculated from $n_\mathrm{lipid} = (1 - 10^{-10})\phi_0 A_\mathrm{initial}$ for Fig. 2, Fig. 3, and Figs. S8 and S9. $n_\mathrm{lipid} = \phi_0 A_\mathrm{initial}$ was used for the other calculations. A_initial is the initial membrane area.

Supplementary Movies

Movie S1. Calculated membrane-crosslinker configurations for the analyses in Fig. 2.

Movie S2. Calculated membrane-crosslinker configurations for the analyses in Fig. 3.

Movie S3. Calculated membrane-crosslinker configurations for the analyses in Figs. S8 and S9.

Movie S4. Calculated membrane-crosslinker configurations for the data indicated by blue circles in Fig. 4C.

Movie S5. Calculated membrane-crosslinker configurations for the data indicated by red diamonds in Fig. 4C.

Movie S6. Calculated membrane-crosslinker configurations for the data indicated by blue circles in Fig. 4F.

Movie S7. Calculated membrane-crosslinker configurations for the data indicated by red diamonds in Fig. 4F.

Movie S8. Calculated membrane-crosslinker configurations for the data indicated by green diamonds in Fig. S13A.

Additional references

51 Petrenko, V. *et al.* Type 2 diabetes disrupts circadian orchestration of lipid metabolism and membrane fluidity in human pancreatic islets. *PLoS Biology* **20**, e3001725 (2022).

52 Snowden, S. G. *et al.* Development and application of high-throughput single cell lipid profiling: a study of SNCA-A53T human dopamine neurons. *Iscience* **23** (2020).

53 Dedhar, S., Jewell, K., Rojiani, M. & Gray, V. The receptor for the basement membrane glycoprotein entactin is the integrin alpha 3/beta 1. *Journal of Biological Chemistry* **267**, 18908-18914 (1992).

54 Calderwood, D. Talin controls integrin activation. *Biochemical Society Transactions* **32**, 434-437 (2004).

55 Petrache, H. I., Dodd, S. W. & Brown, M. F. Area per lipid and acyl length distributions in fluid phosphatidylcholines determined by 2H NMR spectroscopy. *Biophysical journal* **79**, 3172-3192 (2000).
